# BOLERO-5: a phase II study of everolimus and exemestane combination in Chinese post-menopausal women with ER + /HER2- advanced breast cancer

**DOI:** 10.1007/s12672-024-01027-8

**Published:** 2024-06-21

**Authors:** Zhi-Ming Shao, Li Cai, Shusen Wang, Xichun Hu, Kunwei Shen, Haibo Wang, Huiping Li, Jifeng Feng, Qiang Liu, Jing Cheng, Xinhong Wu, Xiaojia Wang, Hongyuan Li, Ting Luo, Jinping Liu, Khalid Amin, Khemaies Slimane, Yongping Qiao, Yongmin Liu, Zhongsheng Tong

**Affiliations:** 1 Fudan University Shanghai Cancer Center, Shanghai, China; 2 Harbin Medical University Cancer Hospital, Harbin, China; 3 Sun Yat-Sen University Cancer Center, Guangzhou, China; 4 Ruijin Hospital Shanghai Jiao Tong University School of Medicine, Shanghai, China; 5 The Affiliated Hospital of Qingdao University, Qingdao, China; 6 Key Laboratory of Carcinogenesis and Translational Research (Ministry of Education), Department of Breast Oncology, Peking University Cancer Hospital and Institute, Beijing, China; 7 Jiang Su Cancer Hospital, Nanjing, China; 8 Second Affiliated Hospital of Sun Yat-Sen University, Guangzhou, China; 9 Wuhan Union Hospital, Wuhan, China; 10 Hubei Cancer Hospital, Tongji Medical College, Huazhong University of Science and Technology and Hubei Provincial Clinical Research Center for Breast Cancer, Wuhan, China; 11 Zhejiang Cancer Hospital, Hangzhou, China; 12 West China Hospital, Sichuan University, Chengdu, China; 13 Sichuan Provincial People’s Hospital, Chengdu, China; 14 Novartis Pharma AG, Basel, Switzerland; 15 China Novartis Institutes for BioMedical Research, Beijing, China; 16 Tianjin Medical University Cancer Institute and Hospital, Tianjin, China

## Abstract

**Background:**

The global BOLERO-2 trial established the efficacy and safety of combination everolimus (EVE) and exemestane (EXE) in the treatment of estrogen receptor positive (ER +), HER2-, advanced breast cancer (ABC). BOLERO-5 investigated this combination in a Chinese population (NCT03312738).

**Methods:**

BOLERO-5 is a randomized, double-blind, multicenter, placebo controlled, phase II trial comparing EVE (10 mg/day) or placebo (PBO) in combination with EXE (25 mg/day). The primary endpoint was progression-free survival (PFS) per investigator assessment. Secondary endpoints included PFS per blinded independent review committee (BIRC), overall survival (OS), overall response rate (ORR), clinical benefit rate (CBR), pharmacokinetics, and safety.

**Results:**

A total of 159 patients were randomized to EVE + EXE (n = 80) or PBO + EXE (n = 79). By investigator assessment, treatment with EVE + EXE prolonged median PFS by 5.4 months (HR 0.52; 90% CI 0.38, 0.71), from 2.0 months (PBO + EXE; 90% CI 1.9, 3.6) to 7.4 months (EVE + EXE; 90% CI 5.5, 9.0). Similar results were observed following assessment by BIRC, with median PFS prolonged by 4.3 months. Treatment with EVE + EXE was also associated with improvements in ORR and CBR. No new safety signals were identified in BOLERO-5, with the incidence of adverse events in Chinese patients consistent with the safety profile of both drugs.

**Conclusion:**

The efficacy and safety results of BOLERO-5 validate the findings from BOLERO-2, and further support the use of EVE + EXE in Chinese post-menopausal women with ER + , HER2- ABC. NCT03312738, registered 18 October 2017.

**Supplementary Information:**

The online version contains supplementary material available at 10.1007/s12672-024-01027-8.

## Introduction

Breast cancer is the most common malignancy in women and one of the leading causes of cancer deaths worldwide [1]. The incidence of breast cancer in China has increased rapidly over the last number of decades, and currently increases by 3–5% annually [2, 3]. Despite evidence suggesting that China demonstrates a lower mortality rate compared to other countries, the size of the population and the current increase in breast cancer incidence reinforce the need for safe and effective treatment options [2].

Approximately 70% of breast cancers are estrogen receptor positive (ER +) and are treated with endocrine therapy (such as nonsteroidal aromatase inhibitors [NSAIs, anastrozole or letrozole] or steroidal aromatase inhibitors [exemestane, EXE]) in combination with CDK4/6 inhibitors in the first-line setting [4–6]. Although these treatments are effective, both de novo and acquired resistance are common, with the majority of patients receiving endocrine therapy developing progressive disease [7, 8]. Many patients retain sensitivity to hormonal agents, even upon disease progression, but sequential endocrine monotherapy achieves only moderate clinical benefits [8, 9]. As such, combination approaches with mammalian target of rapamycin (mTOR) inhibitors have been developed as a second-line treatment option [8, 10]. The PI3K/Akt/mTOR pathway plays a central role in breast cancer cell proliferation and progression [11, 12], providing a strong rationale for combining endocrine therapy with mTOR inhibition [13]. The PI3K/Akt/mTOR pathway is also implicated in the development of resistance to endocrine therapy, further supporting the rationale for mTOR inhibition in the treatment of ER + breast cancer [14, 15]. Everolimus (EVE), an mTOR inhibitor, has demonstrated efficacy in enhancing the effects of endocrine therapy in both pre-clinical models and clinical trials in breast cancer [12, 16, 17].

The global, pivotal, phase III Breast Cancer Trials of OraL EveROlimus-2 (BOLERO-2) trial investigated a combination strategy of EVE and EXE for the treatment of postmenopausal women with locally advanced/metastatic, hormone receptor positive (HR +) disease progressing after anastrozole or letrozole [18]. This study established the efficacy and safety of the EVE and EXE treatment combination, with significantly improved progression-free survival (PFS) observed in patients treated with EVE + EXE versus placebo (PBO) + EXE, and no serious toxicity reported [14, 18]. By investigator assessment, treatment with EVE + EXE prolonged PFS from 3.2 months to 7.8 months (HR, 0.45; 95% CI 0.38, 0.54; *p* < 0.0001) [18]. Similar results were observed following central assessment, with PFS prolonged from 4.1 months to 11.0 months (HR, 0.38; 95% CI 0.31, 0.48; p < 0.0001) [18].

The results of the BOLERO-2 trial led the FDA and EMA to approve combination EVE + EXE for the treatment of HR + , HER-2- advanced breast cancer (ABC) in 2012. This treatment combination is also recommended by both the National Comprehensive Cancer Network (NCCN) and the ESO-ESMO (European School of Oncology—European Society for Medical Oncology) guidelines for postmenopausal women with HR + , HER2- ABC with progressive disease on NSAIs [4, 5].

Ethnicity is well-known to impact treatment efficacy and safety, supporting the opinion that potential inter-ethnic differences in anti-cancer drug effect should be considered [19, 20]. A *post-hoc* analysis of the BOLERO-2 study was therefore conducted to assess the combination of EVE + EXE in Asian versus non-Asian patients [21]. The results of these analyses were consistent with the primary results of BOLERO-2, which demonstrated improved PFS with EVE + EXE in both Asian and non-Asian patients, with no significant differences in the incidence of most adverse events (AEs) observed between Asian and non-Asian patients. However, some AEs such as stomatitis, nasopharyngitis, pneumonitis, and interstitial lung disease were reported more frequently in Asian patients [21]. Combination EVE + EXE provided substantial clinical benefit in both an Asian and non-Asian patient population, representing an improvement in the management of postmenopausal women with HR + , HER2- ABC progressing on NSAIs, regardless of ethnicity [18, 21].

The China-specific BOLERO-5 study aimed to confirm the efficacy and safety of combination treatment with EVE + EXE (seen in BOLERO-2) in postmenopausal Chinese women with locally advanced/metastatic, ER + , HER2- disease following recurrence or progression on anastrozole or letrozole.

## Methods

### Study design and participants

**Fig. 1:**
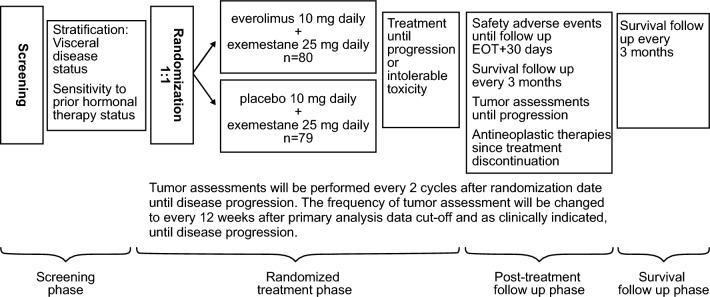
BOLERO-5 study design. *EOT,* end of treatment

BOLERO-5 is a double-blind, randomized, phase II study evaluating combination EVE + EXE in a Chinese population of postmenopausal women, and was conducted at 15 clinical sites across China. Patients were randomized 1:1 to receive either EVE + EXE (10 mg/day and 25 mg/day, respectively) or PBO + EXE (25 mg/day) as oral tablets (Fig. 1). EVE and placebo were identical in packaging, labeling, schedule of administration and in appearance. Patients were registered into Interactive Response Technology (IRT), which assigned a randomization number to the patient used to link the patient to a treatment arm. Randomization was stratified by the presence of visceral disease (lung, liver, brain, pleural and peritoneal involvement) and sensitivity to prior hormonal therapy. Sensitivity to prior hormonal therapy was defined as either A) documented clinical benefit (complete response, partial response, stable disease ≥ 24 weeks) to at least one prior hormonal therapy in the advanced setting, or B)  ≥ 24 months of adjuvant hormonal therapy prior to recurrence.

This study (ClinicalTrials.gov: NCT03312738) was conducted in accordance with the Declaration of Helsinki and the ICH E6 Guidelines for Good Clinical Practice. The study protocol and all amendments were approved by the Independent Ethics Committee or Institutional Review Board of each investigative site. Written informed consent was obtained from each patient before any study-specific procedure was performed. The study was designed and sponsored by Novartis Pharmaceuticals, who collected and analyzed the data in conjunction with the authors.

Postmenopausal women (≥18 years) with histological/cytological confirmation of ER + , HER2- breast cancer and recurrence/progression on prior NSAI (letrozole or anastrozole) were included is this study. These were defined as recurrence while on or within one year of end of adjuvant NSAI treatment, or progression while on or within one month of end of prior NSAI treatment in the advanced or metastatic setting. Key exclusion criteria included HER2-overexpression, previous treatment with more than one line of chemotherapy for ABC, prior treatment with EXE, mTOR inhibitors, PI3K inhibitors, or AKT inhibitors, and known hypersensitivity to mTOR inhibitors.

Treatment continued until disease progression (assessed by Response Evaluation Criteria In Solid Tumors [RECIST] 1.1), unacceptable toxicity, death or treatment discontinuation for any other reason. Patients were permitted to withdraw from the study at any time. Everolimus dose adjustments were also permitted to allow patients to continue study treatment. Only two dose reductions were permitted per patient. Patients with therapy interruptions for more than four weeks were permanently discontinued from the study. To mitigate the increased risk of stomatitis associated with EVE, all patients were advised to practice good oral hygiene and were recommended to use a preventive dexamethasone mouthwash.

### Endpoints

The primary endpoint of the study was PFS by local assessment (per RECIST 1.1 criteria), with PFS per blinded independent review committee (BIRC) as a secondary endpoint. PFS was defined as time from the date of randomization to the date of first documented progression or death due to any cause, whichever occurred first. Overall survival (OS), overall response rate (ORR), clinical benefit rate (CBR), time to response, and duration of response (DOR) were all secondary endpoints, and were assessed both locally and by BIRC. Secondary endpoints also included time to deterioration in Eastern Cooperative Oncology Group (ECOG) performance status, AEs, and laboratory abnormalities. The pharmacokinetics of combination EVE + EXE were also assessed.

### Study assessments

Tumor evaluation (based on computed tomography or magnetic resonance imaging) was carried out at baseline (within 21 days of treatment initiation) and every 8 weeks (± 7 days) post-randomization until radiological progression, per local assessment. Objective tumor response and disease progression were assessed per RECIST version 1.1. All patients were followed for survival at least every three months post-randomized treatment or post-treatment follow-up, unless they discontinued due to death, withdrawal of consent or were lost to follow-up.

Safety assessments included AEs, serious AEs (SAEs), laboratory analyses (hematology, serum chemistry, coagulation), vital signs and physical assessments, and were carried out within 7 days of tumor assessment. Information on patient deaths was also collected. All patients were followed-up for safety up to 30 days following the last dose of treatment. AEs were graded according to the NCI Common Terminology Criteria for Adverse Events (CTCAE) v4.03. An AE was defined as the appearance of (or worsening of any pre-existing) undesirable sign(s), symptom(s), or medical condition(s) that occur after patient’s signed informed consent has been obtained.

### Statistical analysis

The primary analysis was planned when approximately 110 PFS events were documented, based on local assessment. As this is a bridging study, an estimation strategy was used over formal hypothesis testing to estimate sample size. Approximately 160 patients were estimated as being needed for randomization to the two treatment arms to observe the 110 PFS events at approximately 22 months post-randomization of the first study patient.

PFS was estimated by Kaplan–Meier analysis using the intention-to-treat (ITT) principle, with median PFS and 90% confidence interval (CI) presented by treatment arm. A Cox regression model (stratified by randomization stratification factors) was used to estimate the hazard ratio (HR) and associated 90% CI for PFS, OS, ORR, and CBR. No hypothesis testing was performed as this was an estimation-based approach. Baseline demographic and disease characteristics data were listed and summarized descriptively by treatment arm. Duration of study treatment exposure, dose intensity, and safety were also summarized by treatment.

### Role of the funding source

Novartis Pharmaceuticals Corporation sponsored the study, designed the study and analyzed the data. All authors had full access to the study data and had the final responsibility for the decision to submit this manuscript for publication. The corresponding author had access to all study data and had final responsibility to submit this manuscript for publication.

## Results

### Patients

**Fig. 2:**
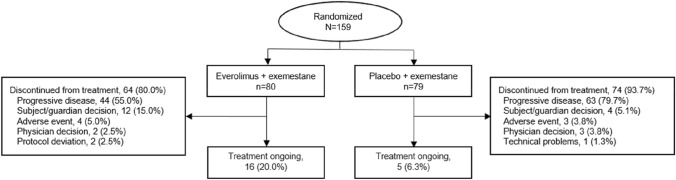
Study profile [Consort diagram]

A total of 159 postmenopausal women were enrolled across multiple centers in China between 15 September 2017 and 31 March 2020 and were randomized to receive either EVE + EXE (n = 80) or PBO + EXE (n = 79) (Fig. 2). Baseline demographic characteristics were well matched between treatment arms (Table 1); median age (range) at baseline was 56.0 years (36–81) and most patients had an ECOG performance score of 1 (EVE + EXE, 61.3%; PBO + EXE, 68.4%). Baseline disease characteristics were also similar between arms, with most patients initiating study treatment within 3 months of last disease progression (EVE + EXE, 91.3%; PBO + EXE, 97.5%). All patients had stage IV disease at study entry and baseline tumor burden was similar between treatment arms. Overall, 71.1% of patients had visceral involvement and 44.7% of patients had bone metastases; most patients had metastases in other sites besides the central nervous system (CNS), bone, lung, liver, and visceral sites (EVE + EXE, 75.0%; PBO + EXE, 81.0%) (Table 1).

**Table 1:** Baseline patient demographics and disease characteristics

	EVE + EXE n = 80	PBO + EXE n = 79	All patients N = 159
Demographic/characteristic			
Age (years), mean (SD)	56.6 (9.1)	56.3 (8.4)	56.4 (8.8)
Age category, n (%)			
18‒ < 65 years	65 (81.3)	68 (86.1)	133 (83.6)
≥ 65 years	15 (18.8)	11 (13.9)	26 (16.4)
Sex, n (%)			
Female	80 (100.0)	79 (100.0)	159 (100.0)
ECOG performance status, n (%)			
0	27 (33.8)	23 (29.1)	50 (31.4)
1	49 (61.3)	54 (68.4)	103 (64.8)
2	4 (5.0)	2 (2.5)	6 (3.8)
Measurable disease at baseline, n (%)			
Yes	6 (7.5)	8 (10.1)	14 (8.8)
No	14 (17.5)	11 (13.9)	25 (15.7)
Combination*	60 (75.0)	60 (75.9)	120 (75.5)
Time between initial diagnosis and first recurrence/metastasis, n (%)			
< 3 months	3 (3.8)	0	3 (1.9)
≥ 3‒ < 6 months	2 (2.5)	2 (2.5)	4 (2.5)
≥ 6 months	75 (93.8)	77 (97.5)	152 (95.6)
Metastatic cancer sites, n (%)			
CNS	2 (2.5)	5 (6.3)	7 (4.4)
Bone	38 (47.5)	33 (41.8)	71 (44.7)
Bone only	4 (5.0)	2 (2.5)	6 (3.8)
Visceral (excluding CNS)	56 (70.0)	57 (72.2)	113 (71.1)
Lung	38 (47.5)	37 (46.8)	75 (47.2)
Liver	29 (36.3)	27 (34.2)	56 (35.2)
Lung and liver	13 (16.3)	12 (15.2)	25 (15.7)
Others	60 (75.0)	64 (81.0)	124 (78.0)
Number of metastatic sites, n (%)			
1	16 (20.0)	11 (13.9)	27 (17.0)
2	29 (36.3)	24 (30.4)	53 (33.3)
3	15 (18.8)	24 (30.4)	39 (24.5)
> 3	20 (25.0)	20 (25.3)	40 (25.2)
Previous surgery, n (%)			
Yes	73 (91.3)	75 (94.9)	148 (93.1)
No	7 (8.8)	4 (5.1)	11 (6.9)
Previous radiotherapy, n (%)			
Yes	46 (57.5)	43 (54.4)	89 (56.0)
No	34 (42.5)	36 (45.6)	70 (44.0)
Previous NSAI, n (%)			
Letrozole	57 (71.3)	50 (63.3)	107 (67.3)
Anastrozole	21 (26.3)	22 (27.8)	43 (27.0)
Letrozole and Anastrozole	2 (2.5)	7 (8.9)	9 (5.7)
Previous hormonal therapy (other than NSAI), n (%)			
Antiestrogen	45 (56.3)	32 (40.5)	77 (48.4)
Tamoxifen	26 (32.5)	16 (20.3)	42 (26.4)
Fulvestrant	12 (15.0)	13 (16.5)	25 (15.7)
Tamoxifen and Fulvestrant	7 (8.8)	3 (3.8)	10 (6.3)
Other	7 (8.8)	5 (6.3)	12 (7.5)
Purpose of most recent treatment, n (%)			
Adjuvant therapy	22 (27.5)	24 (30.4)	46 (28.9)
Advanced/metastatic disease	46 (57.5)	37 (46.8)	83 (52.2)
Palliative	10 (12.5)	14 (17.7)	24 (15.1)
Not applicable	2 (2.5)	4 (5.1)	6 (3.8)
Number of previous therapies in any setting, n (%)			
1	3 (3.8)	2 (2.5)	5 (3.1)
2	2 (2.5)	2 (2.5)	4 (2.5)
3	2 (2.5)	3 (3.8)	5 (3.1)
> 3	73 (91.3)	72 (91.1)	145 (91.2)

BL, baseline; cm, centimeter; CNS, central nervous system, ECOG, Eastern Cooperative Oncology Group; EVE, everolimus; EXE, exemestane; kg, kilogram; n, number of patients; N, total number of patients; NSAI, non-steroidal aromatase inhibitor; PBO, placebo; SD, standard deviation

^*^Patients with measurable target lesions plus any type of non-measurable disease

All patients were heavily pre-treated, with most patients receiving > 3 therapies prior to enrollment (EVE + EXE, 91.3%; PBO + EXE, 91.1%). As required by the study protocol, all patients were treated with at least one NSAI regimen prior to study entry. Other common prior therapies included tamoxifen (EVE + EXE, 32.5%; PBO + EXE, 20.3%) and fulvestrant (EVE + EXE, 15.0%; PBO + EXE, 16.5%). For the majority of patients, the most recent prior therapy was received in the metastatic setting (EVE + EXE, 57.5%; PBO + EXE, 46.8%) and 30.2% of patients had received chemotherapy in a metastatic setting (EVE + EXE, 36.3%; PBO + EXE, 24.1%).

A total of 116 PFS events occurred by the data cutoff of 19 May 2020, with a median follow-up of 14.5 months. Most patients (n = 138; 86.8%) had discontinued randomized treatment by data cutoff, with disease progression the primary reason for discontinuation (EVE + EXE, 55.0%; PBO + EXE, 79.7%). At data cutoff, median duration (range) of EVE therapy in the EVE + EXE arm was 16.1 weeks (2.4‒95.0), while median duration (range) of EXE therapy was 17.1 weeks (range: 2.4–95.1). For the PBO + EXE arm, median duration of EXE therapy was 8.4 weeks (range: 2.1–96.1); the same median duration and range were observed for PBO therapy. Both treatment arms achieved a median relative dose intensity of 100%, with a median dose intensity of 10 mg/day for PBO and EVE, and 25 mg/day for EXE. Mean (SD) dose intensity in the EVE + EXE arm was 9.1 mg/day (1.45) for EXE and 25.0 mg/day (0.00) for EVE, whereas in the PBO + EXE arm it was 10.0 mg/day (0.14) for PBO and 25.0 mg/day (0.00) for EXE. In the EVE + EXE arm, 23 patients (28.8%) were exposed to EVE and 28 patients (35.0%) were exposed to EXE for a period of ≥ 32 weeks; in the PBO + EXE arm, just 13 patients (16.5%) received treatment for ≥ 32 weeks.

Most patients (62.5%) in the EXE + EVE arm had at least one dose adjustment (reduction and/or temporary interruption) of EVE, while 17.7% of patients in the PBO + EXE arm had at least one dose adjustment of PBO. At least one EVE dose reduction was required in 32.5% of patients in the EVE + EXE arm compared to 2.5% of patients with at least one reduction of PBO in the PBO + EXE arm. At least one dose interruption in EVE was required in 56.3% of patients in the EVE + EXE arm versus 17.7% of patients with at least one interruption in PBO in the PBO + EXE arm. In the EVE + EXE arm, the primary reason for EVE dose adjustments or interruptions was AEs (responsible for 21.3% of reductions and 47.5% of interruptions). More patients had at least one dose interruption of EXE in the EVE + EXE arm (40.0%) vs the PBO + EXE arm (16.5%); no patient in either arm required an EXE dose reduction.

### Progression-free survival

**Fig. 3:**
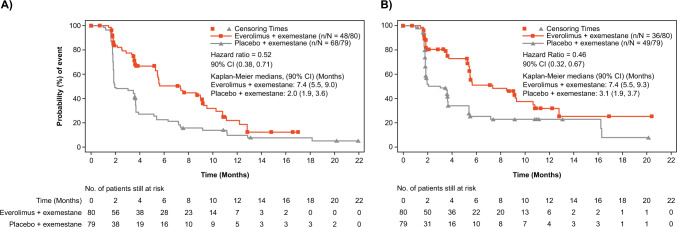
Progression-free survival based on investigator assessment (**A**) and based on BIRC assessment (**B**). *BIRC,* Blinded Independent Central Review; *CI,* confidence interval

By investigator assessment, treatment with EVE + EXE provided a clinically meaningful PFS benefit. Treatment with EVE + EXE reduced the risk of disease progression or death by 48% (HR 0.52; 90% CI, 0.38, 0.71), and prolonged median PFS by 5.4 months (from 2.0 months with PBO + EXE [90% CI 1.9, 3.6] to 7.4 months with EVE + EXE [90% CI 5.5, 9.0]) (Fig. 3A). Similar results were observed following BIRC assessment; treatment with EVE + EXE reduced the risk of disease progression or death by 54% (HR 0.46; 90% CI 0.32, 0.67) and prolonged median PFS by 4.3 months (from 3.1 months with PBO + EXE [90% CI 1.9, 3.7] to 7.4 months with EVE + EXE [90% CI 5.5, 9.3]) (Fig. 3B). The PFS rate at 12 months was 25.5% in the EVE + EXE arm versus 7.6% in the PBO + EXE arm per investigator assessment.

### Overall survival

The OS analysis was considered immature at data cutoff due to the small number of deaths reported (EVE + EXE, n = 22, 27.5%; PBO + EXE, n = 21, 26, 6%).

### Overall response rate and clinical benefit rate

ORR (complete response + partial response) as assessed by the investigator and based on RECIST 1.1 criteria was 8.8% (90% CI 4.2, 15.8) in patients receiving EVE + EXE versus 1.3% (90% CI 0.1, 5.9) in those receiving PBO + EXE (Table 2). CBR (complete response + partial response + stable disease ≥ 24 weeks + non-complete response/non-progressive disease) per investigator assessment was higher in patients treated with EVE + EXE (35.0%; 90% CI 26.1, 44.7) than in those treated with PBO + EXE (16.5%, 90% CI 10.0, 24.9) (Table 2). Similar results were observed following BIRC assessment of ORR and CBR (Table 2). Progressive disease was the best overall response for 16.3% of patients receiving EVE + EXE and 49.4% of patients receiving PBO + EXE.

**Table 2:** Overall response rate and clinical benefit rate

	EVE + EXE n = 80	PBO + EXE n = 79
Per investigator assessment		
ORR, % (90% CI)	8.8 (4.2, 15.8)	1.3 (0.1, 5.9)
CBR, % (90% CI)	35.0 (26.1, 44.7)	16.5 (10.0, 24.9)
Per BIRC assessment		
ORR, % (90% CI)	8.8 (4.2, 15.8)	2.5 (0.5, 7.8)
CBR, % (90% CI)	31.3 (22.7, 40.8)	11.4 (6.1, 19.0)

ORR is defined as the proportion of patients with the best overall response of completed response or partial response per RECIST 1.1. CBR is defined as the proportion of patients with best overall response of CR, PR, or overall lesion response of stable disease (SD for measurable disease and non-CR/non-PD for non-measurable disease) ≥ 24 weeks. DOR is defined as the time from first documented response (PR or CR) to the date of first documented disease progression or death due to any cause

CBR, clinical benefit rate; CI, confidence interval; CR, complete response; DOR, duration of response; EVE, everolimus; EXE, exemestane; PBO, placebo; PD, progressive disease; PR, partial response; RECIST, Response Evaluation Criteria In Solid Tumors; SD, stable disease

### Time to response, duration of response and ECOG performance status

Analyses of the Kaplan–Meier estimate of the time to response were not performed due to the low number of responses in both arms (8 partial responses per investigator assessment; one complete response and 8 partial responses per BIRC assessment). Time to response and duration of response data are shown in Table S1. Deterioration in ECOG performance status by ≥ 1 point was noted in few patients (8/80) in the EVE + EXE arm and in no patients in the PBO + EXE arm (Figure S1).

### ﻿Safety

The majority of patients reported at least one AE during the study (EVE + EXE, 98.8%; PBO + EXE, 84.8%), many of which were Grade 1 or 2 in severity (EVE + EXE, 46.2%; PBO + EXE, 70.9%). The most common AEs associated with EVE + EXE included increased aspartate aminotransferase (45%), hyperglycemia (43.8%), stomatitis (33.8%), increased alanine aminotransferase (31.3%) and anemia (31.3%). Reported rates of grade ≥ 3 AEs were higher overall in the EVE + EXE arm versus the PBO + EXE arm (53.8% versus 29.1%). The incidence of treatment-related adverse events (TRAEs) in the BOLERO-5 study was consistent with the known safety profile of both drugs, with no new safety signals identified in Chinese patients (Table 3). Most TRAEs were Grade 1 or Grade 2, but the incidence of Grade 3 TRAEs was higher in the EVE + EXE arm (45.0%) compared to the PBO + EXE arm (11.4%). The most common Grade ≥ 3 TRAEs in the EVE + EXE arm versus the PBO + EXE arm were hyperglycemia (10.0% versus 0%), hypokalemia (6.3% versus 0%), hypophosphatemia (6.3% versus 0%), stomatitis (7.5% versus 1.3%) and pneumonia (5% versus 0).

**Table 3:** Treatment-related adverse events by preferred term (occurring in ≥ 20% of patients in either treatment arm)

Treatment-related adverse events	EVE + EXE n = 80		PBO + EXE n = 79	
	All grades, n (%)	Grade ≥ 3, n (%)	All grades, n (%)	Grade ≥ 3, n (%)
≥ 1 Treatment-related AE	79 (98.8)	36 (45.0)	46 (58.2)	9 (11.4)
Hyperglycemia*	34 (42.5)	8 (10.0)	2 (2.5)	0
Aspartate aminotransferase increased*	32 (40.0)	1 (1.3)	11 (13.9)	1 (1.3)
Stomatitis*	27 (33.8)	6 (7.5)	5 (6.3)	1 (1.3)
Alanine aminotransferase increased*	23 (28.8)	1 (1.3)	9 (11.4)	1 (1.3)
Anemia	19 (23.8)	3 (3.8)	8 (10.1)	3 (3.8)
Hypercholesterolemia*	19 (23.8)	0	0	0
Mouth ulceration*	19 (23.8)	0	2 (2.5)	0
Weight decreased*	17 (21.3)	1 (1.3)	2 (2.5)	0

A patient with multiple severity grades for an AE is only counted under the maximum grade. Adverse events occurring more than 30 days after the discontinuation of study treatment are not summarized. Adverse events were described as per MedDRA version 23.0, CTCAE version 4.03

^*^AE occurs in a greater proportion of patients randomized to everolimus plus exemestane (≥ 15% difference versus placebo plus exemestane)

AE, adverse event

The most common adverse events of special interest (AESIs) all occurred more frequently in the EVE + EXE arm (Table S2). These included stomatitis (reported in 73.8% of patients in the EVE + EXE arm versus 11.4% of patients in the PBO + EXE arm), cytopenia (53.8% versus 24.1%), hyperglycemia/new onset diabetes mellitus (51.3% versus 8.9%), dyslipidemia (47.5% versus 7.6%), and severe infections (45.0% versus 17.7%). Non-infectious pneumonitis, frequently associated with mTOR inhibitors, was reported in 19 patients (23.8%) randomized to EVE + EXE and all cases were considered to be treatment-related. The majority of cases were mild in nature, with only 1 case (1.3%) of Grade ≥ 3 non-infectious pneumonitis reported. No patients in the PBO + EXE arm experienced non-infectious pneumonitis.

No unexpected findings were reported in relation to clinical laboratory or vital signs. Hematological abnormalities were more commonly observed in the EVE + EXE arm than in the PBO + EXE arm. Decreased hemoglobin was the most common abnormality (EVE + EXE, 57.5%; PBO + EXE, 24.1%), followed by decreased absolute lymphocyte count (EVE + EXE, 45.0%; PBO + EXE, 22.8%). Clinical abnormalities were also more frequent in patients treated with EVE + EXE versus PBO + EXE, and included increased triglycerides (63.8% versus 25.3%) and increased cholesterol (83.8% versus 29.1%).

The incidence of AEs leading to permanent study discontinuation was low in both treatment arms (EVE + EXE, 11.3%; PBO + EXE, 3.8%). The most frequent AEs resulting in discontinuation were pneumonia (n = 3 in the EVE + EXE arm) and bilirubin increase (n = 2 in the EVE + EXE arm). Serious adverse events (SAEs) were reported more frequently in the EVE + EXE arm vs the PBO + EXE arm (20% versus 12.7% reporting ≥ one SAE). The most common SAEs in the EVE + EXE arm were pneumonia (7.5%) and interstitial lung disease (2.5%). A total of five deaths occurred during the study treatment (EVE + EXE, n = 3; PBO + EXE, n = 2), all of which were attributed to the underlying malignancy.

## ﻿Discussion

The BOLERO-5 study provides strong evidence for the efficacy and safety of EVE + EXE in Chinese post-menopausal women with ER + , HER2-, locally advanced, recurrent, or metastatic breast cancer following recurrence or progression on prior letrozole or anastrozole. The global BOLERO-2 study established the efficacy and safety of combination of EVE + EXE [14, 18] and led to the approval of EVE for the treatment of post-menopausal women with HR + , HER2- ABC. The Asian sub-population analysis of BOLERO-2 (N = 143), as well as the recent Phase IIIB EVEREXES trial of this treatment combination in Asian patients (N = 199), support the use of combination treatment with EXE + EVE to improve PFS without compromising quality of life (QoL) [18, 21–23], representing a significant advancement in the management of ABC. Following the publication of the BOLERO-2 results, several international guidelines (including the NCCN and ESO-ESMO ABC5 treatment guidelines) recommended combination treatment with EVE + EXE for this population [4, 5].

In BOLERO-5, combination EVE + EXE achieved remarkably similar PFS to that observed in the global BOLERO-2 trial (median PFS per investigator assessment: 7.4 months versus 7.8 months, respectively) and provided clinically meaningful benefits for the Chinese patients enrolled [14, 18, 21]. Treatment with EVE + EXE yielded a 48% risk reduction in PFS (per investigator assessment) and prolonged median PFS by 5.4 months, from 2.0 months (PBO + EXE) to 7.4 months (EVE + EXE). The PFS rate at 12 months was also numerically greater in the EVE + EXE arm compared to the PBO + EXE arm, indicating a more sustained treatment benefit with this combination. The magnitude of this PFS benefit compares favorably with other studies of everolimus and anti-estrogen therapy in this setting [16, 17], and also with the alternative treatment options available for these patients [8, 9, 24]. Patients receiving combination treatment with EVE + EXE also achieved greater ORR and CBR, further supporting the primary PFS outcome. Similar to PFS data, the observed ORR for EVE + EXE in BOLERO-5 was very similar to that reported in the BOLERO-2 global trial (8.8% versus 9.5%, respectively). The similarity of results between investigator and BIRC assessment of primary and secondary outcomes lends further credence to the robust nature of these data. While the OS data were immature at the primary cutoff, long-term follow up will enable further evaluation of the benefits of EVE + EXE combination treatment. The publication of the pharmacokinetic results of this trial will also provide an opportunity to evaluate the impact of combination EVE + EXE on estrogen concentration.

The combination of EVE + EXE was well tolerated in this Chinese patient population, with the incidence of AEs consistent with that previously reported with EVE and other rapamycin analogues [25], as well as with the safety profile of this treatment combination in HR + , HER2- ABC [14, 18, 21]. While the incidence of AEs was increased in the EVE + EXE group versus PBO + EXE, analysis of patient-reported outcomes in a similar patient population in the BOLERO-2 study revealed that QoL was maintained with this treatment combination, despite the increase in AEs [22]. Thus, the clinical efficacy of combination EVE + EXE may outweigh the potential toxicity, particularly when the impact on patient QoL is negligible. Most AEs reported in this study were Grade 1 or 2 in severity and could be managed with appropriate dose adjustments (reduction and/or interruption) and supportive therapies. The low discontinuation rate due to AEs in the EVE + EXE arm indicates that most AEs were well managed, allowing patients to continue the treatment as long as it provided clinical benefit.

Some differences in AESIs were reported between BOLERO-5 and BOLERO-2, although these differences were < 15%. These were for stomatitis (73.8% versus 68.0%), hemorrhages (16.3% versus 30.3%), and non-infectious pneumonitis (23.8% versus 21.8%) [26]. These events can all be effectively managed in this setting using clinically-defined management strategies [27–29]. A higher incidence of hyperglycemia and lower incidence of stomatitis were observed in BOLERO-5 compared with the BOLERO-2 Asian population. Stomatitis was reported in 73.8% of patients administered EVE + EXE in BOLERO-5 compared to 84.7% of patients in the Asian sub-population of BOLERO-2 [26]. It is reassuring that the incidence of stomatitis was lower in BOLERO-5, which can likely be attributed to the implementation of prophylactic treatment with corticosteroid-based mouthwash and to the improved management of this event over time [30]. However, no data on patient adherence to mouthwash usage were collected. The higher incidence of hyperglycemia in BOLERO-5 versus the Asian patients of BOLERO-2 (51.3% versus 11.2%) can possibly be explained by the evolution in case retrieval strategies used for mapping preferred terms to AESI [26]. The BOLERO-2 Asian subgroup analysis case retrieval strategy included hyperglycemia, diabetes mellitus, blood glucose increased, glycosuria, type 2 diabetes mellitus, and glucose urine present under the AESI of hyperglycemia/new onset diabetes mellitus, while the BOLERO-5 case retrieval strategy included hyperglycemia, diabetes mellitus, blood glucose increased, diabetic ketosis, glucose tolerance impaired, and glycosylated hemoglobin increased [26]. Of note, starting treatment with EVE at 5 mg/day rather than 10 mg/day may increase treatment compliance due to good tolerability and similar effectiveness [31].

The efficacy of combination EVE + EXE observed in BOLERO-5 was remarkably similar to the global BOLERO-2 trial, particularly the results of the BOLERO-2 Asian sub-population analysis [18, 21]. The safety profile of this treatment combination in a Chinese population was also largely consistent with the safety profile observed in the global BOLERO-2 trial [18, 21]. Similar efficacy results were also observed in the EVEREXES trial of combination EVE + EXE conducted in Asia, providing further support for this treatment combination in Chinese patients [23].

Treatment guidelines currently recommend the use of endocrine therapy in combination with CDK4/6 inhibitors in the first-line setting [4–6]. This study was carried out before this recommendation was implemented, and as such—as in the BOLERO-2 study [18]–no enrolled patients had received CDK4/6 inhibitors prior to the study start. No clinical trials have been carried out to date assessing the efficacy of EVE + EXE in patients pretreated with CDK4/6 inhibitors; however, a retrospective analysis of 622 patients with HR +, HER2- metastatic breast cancer showed that EVE + EXE was effective in patients who had received endocrine therapy alone and in combination with CDK4/6 inhibitors [32]. Similar results showing no impact of previous treatment with CDK4/6 inhibitors in OS were obtained in two smaller retrospective studies [33, 34].

This study is not without its limitations, and these results should be interpreted with a degree of caution. The small sample sizes in each group, in particular the small number of patients classed as responders, limit the interpretation of these data. Secondly, no QoL data were collected during this study, so the impact of AEs on the QoL of the Chinese postmenopausal women enrolled in this trial is unknown.

In conclusion, treatment with EVE + EXE provided a clinically meaningful PFS benefit in this heavily pre-treated population of Chinese patients with ER + , HER2- breast cancer, further validating the clinical benefit reported in the global BOLERO-2 trial. Combination treatment with EVE + EXE thus offers a significant improvement in the management of Chinese patients with ER + , HER2- breast cancer.

### Supplementary Information

Supplementary file 1 (PDF 252 KB)

Supplementary file 2 (PDF 20602 KB)

Supplementary file 3 (DOC 218 KB)

## Data Availability

The dataset supporting the conclusions of this article is included within the article (and its additional file(s). Novartis is committed to sharing with qualified external researchers, access to patient-level data and supporting clinical documents from eligible studies. These requests are reviewed and approved by an independent review panel on the basis of scientific merit. All data provided are anonymized to respect the privacy of patients who have participated in the trial in line with applicable laws and regulations. Trial data availability is according to the criteria and process described on www.clinicalstudydatarequest.com.
