# Supplementary material for: BOLERO-5: a phase II study of everolimus and exemestane combination in Chinese post-menopausal women with ER + /HER2- advanced breast cancer
Source: Discov Oncol. 2024 Jun 21;15:237. doi: 10.1007/s12672-024-01027-8 (PMC11192707; doi:10.1007/s12672-024-01027-8)
Supplement: Supplementary file 1 — (PDF 252 KB) [file 12672_2024_1027_MOESM1_ESM.pdf]

Supplementary Materials

Supplementary Tables

Table S1: Time to response and duration of response.

	EVE + EXE		PBO + EXE	
	**n**	**Range**	**n**	**Range**
**Time to response**				
Investigator assessment	7	1.7–5.3 months	1	1.8 months
BIRC assessment	7	0.5–7.4 months	2	1.7–3.6 months
**Duration of response**				
Investigator assessment	7	57–462 days	1	562 days
BIRC assessment	7	57–281 days	2	221–337 days

BIRC, blinded independent review committee.

Table S2: Adverse events of special interest*.

Safety topic	EVE + EXE n=80		PBO + EXE n=79	
	All Grades n (%)	Grade ≥3 n (%)	All Grades n (%)	Grade ≥3 n (%)
Cytopenia	43 (53.8)	9 (11.3)	19 (24.1)	7 (8.9)
Dyslipidemia	38 (47.5)	0	6 (7.6)	0
Hemorrhages	13 (16.3)	0	3 (3.8)	0
Hyperglycemia/ new onset of diabetes mellitus	41 (51.3)	9 (11.3)	7 (8.9)	1 (1.3)
Hypersensitivity (anaphylactic reactions)	22 (27.5)	1 (1.3)	5 (6.3)	1 (1.3)
Hypophosphatemia	9 (11.3)	5 (6.3)	0	0
Increased creatinine / proteinuria / renal failure	9 (11.3)	0	4 (5.1)	0
Muscle wasting / Muscle loss	22 (27.5)	1 (1.3)	9 (11.4)	1 (1.3)
Non-infectious pneumonitis	19 (23.8)	1 (1.3)	0	0
Pre-existing infection (reactivation, aggravation, or exacerbation)	8 (10.0)	5 (6.3)	1 (1.3)	1 (1.3)
Severe infections	36 (45.0)	3 (3.8)	14 (17.7)	0
Stomatitis	59 (73.8)	9 (11.3)	9 (11.4)	1 (1.3)

*Adverse events of special interest (AESI) evolve over time based on the growing body of safety information accumulated and are based on the current electronic Case Retrieval Strategy (CRS) which groups several preferred terms into each AESI.

A patient with multiple severity grades for an AE is only counted under the maximum grade. Adverse events occurring more than 30 days after the discontinuation of study treatment are not summarized. Adverse events were described as per MedDRA version 23.0, CTCAE version 4.03.

Supplementary Figures

Figure S1: Time to definitive deterioration in ECOG performance status (deterioration of performance status ≥1 point).

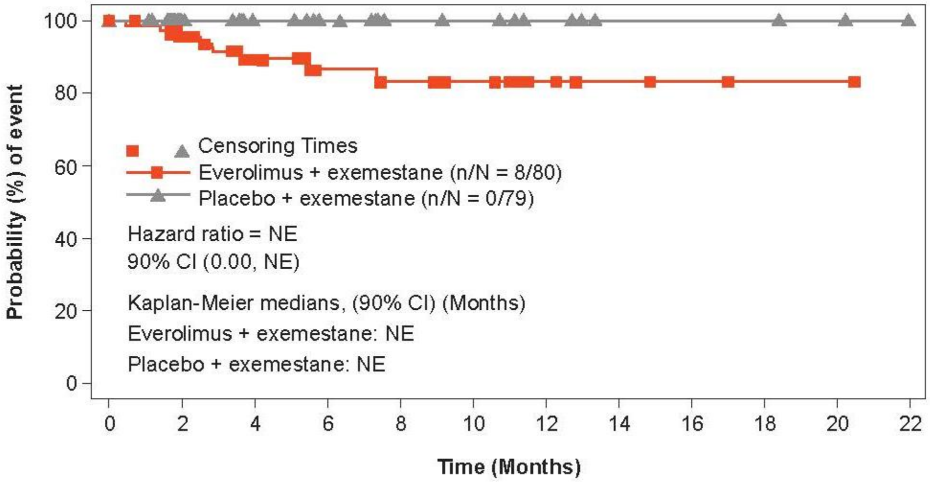

No. of patients still at risk

Time (Months)	0	2	4	6	8	10	12	14	16	18	20	22
Everolimus + exemestane	80	50	36	25	22	15	8	3	2	1	1	0
Placebo + exemestane	79	39	19	15	10	9	6	3	3	3	2	0

CI, confidence interval; NE, not estimable.
