# Supplementary material for: BOLERO-5: a phase II study of everolimus and exemestane combination in Chinese post-menopausal women with ER + /HER2- advanced breast cancer
Source: Discov Oncol. 2024 Jun 21;15:237. doi: 10.1007/s12672-024-01027-8 (PMC11192707; doi:10.1007/s12672-024-01027-8)
Supplement: Supplementary file 2 — (PDF 20602 KB) [file 12672_2024_1027_MOESM2_ESM.pdf]

NOVARTIS

Clinical Development

RAD001 (everolimus)

Oncology Clinical Protocol CRAD001Y2202 / NCT03312738

A randomized, double-blind, placebo controlled, phase II study of Everolimus in combination with Exemestane in the treatment of Chinese postmenopausal women with estrogen receptor positive, HER-2 negative, locally advanced, recurrent, or metastatic breast cancer after recurrence or progression on prior letrozole or anastrozole

Authors	[redacted]
Document type	Amended Protocol Version
EUDRACT number	Not applicable
Version number	01 (Clean)
Development phase	II
Document status	Final
Release date	23-May-2017

Property of Novartis
Confidential
May not be used, divulged, published or otherwise disclosed
without the consent of Novartis

[redacted]

Table of contents

Table of contents 2
List of figures 5
List of tables 5
List of abbreviations 7
Glossary of terms 11
Protocol summary 12
Amendment 1 (23-May-2017) 19
1 Background 23
1.1 Overview of disease pathogenesis, epidemiology and current treatment 23
1.1.1 Epidemiology of breast cancer 23
1.1.2 Treatment options for hormone receptor positive advanced breast cancer 23
1.1.3 Role of mammalian target of rapamycin in ER+ ABC 24
1.2 Introduction to investigational treatment(s) and other study treatment(s) 25
1.2.1 Overview of everolimus 26
1.2.2 Overview of exemestane 33
2 Rationale 34
2.1 Study rationale and purpose 34
2.2 Rationale for the study design 36
2.3 Rationale for dose and regimen selection 36
2.4 Rationale for choice of combination drugs 36
2.5 Risks and benefits 37
3 Objectives and endpoints 38
4 Study design 39
4.1 Description of study design 39
4.2 Timing of interim analyses and design adaptations 43
4.3 Definition of end of study 43
4.4 Early study termination 43
5 Population 43
5.1 Patient population 43
5.2 Inclusion criteria 44
5.3 Exclusion criteria 45
6 Treatment 47
6.1 Study treatment 47
6.1.1 Dosing regimen 47

[redacted]

6.1.2 Ancillary treatments ... 47
6.1.3 Rescue medication ... 47
6.1.4 Guidelines for continuation of treatment ... 47
6.1.5 Treatment duration ... 48
6.2 Dose escalation guidelines ... 48
6.3 Dose modifications ... 48
6.3.1 Dose modification and dose delay ... 48
6.3.2 Additional management and follow-up for selected toxicities ... 51
6.3.3 Exemestane ... 57
6.3.4 Follow-up for toxicities ... 58
6.4 Concomitant medications ... 58
6.4.1 Prohibited concomitant therapy ... 58
6.4.2 Use of bisphosphonates ... 59
6.4.3 Other concomitant medications ... 59
6.5 Patient numbering, treatment assignment or randomization ... 61
6.5.1 Patient numbering ... 61
6.5.2 Treatment assignment or randomization ... 62
6.5.3 Treatment blinding ... 62
6.6 Study treatment preparation and dispensation ... 63
6.6.1 Study treatment packaging and labeling ... 63
6.6.2 Drug supply and storage ... 63
6.6.3 Study treatment compliance and accountability ... 64
6.6.4 Disposal and destruction ... 64
7 Visit schedule and assessments ... 64
7.1 Study flow and visit schedule ... 64
7.1.1 Molecular pre-screening ... 68
7.1.2 Screening ... 68
7.1.3 Treatment period ... 70
7.1.4 Discontinuation of study treatment ... 70
7.1.5 Withdrawal of consent ... 71
7.1.6 Follow-up period ... 71
7.1.7 Lost to follow-up ... 72
7.2 Assessment types ... 72
7.2.1 Efficacy assessments ... 72
7.2.2 Safety and tolerability assessments ... 75
7.2.3 Pharmacokinetics ... 78

[redacted]

7.2.4 Biomarkers 81
7.2.5 Resource utilization 81
7.2.6 Patient reported outcomes 81
8 Safety monitoring and reporting 81
8.1 Adverse events 81
8.1.1 Definitions and reporting 81
8.1.2 Laboratory test abnormalities 82
8.2 Serious adverse events 83
8.2.1 Definitions 83
8.2.2 Reporting 83
8.3 Emergency unblinding of treatment assignment 84
8.4 Pregnancies 85
8.5 Warnings and precautions 85
8.6 Data Monitoring Committee 85
8.7 Steering Committee 85
9 Data collection and management 85
9.1 Data confidentiality 85
9.2 Site monitoring 86
9.3 Data collection 86
9.4 Database management and quality control 87
10 Statistical methods and data analysis 87
10.1 Analysis sets 88
10.1.1 Full Analysis Set 88
10.1.2 Safety set 88
10.1.3 Pharmacokinetic set (PAS) 88
10.2 Patient demographics/other baseline characteristics 88
10.3 Treatments (study treatment, concomitant therapies, compliance) 88
10.4 Primary objective 89
10.4.1 Variable 89
10.4.2 Statistical hypothesis, model, and method of analysis 89
10.4.3 Handling of missing values/censoring/discontinuations 89
10.4.4 Supportive and sensitivity analyses 90
10.5 Secondary objectives 90
10.5.1 Secondary efficacy objectives 90
10.5.2 Safety objectives 91
10.5.3 Pharmacokinetics 93

[redacted]

10.6 Sample size calculation.....94
10.7 Power for analysis of key secondary variables.....94
11 Ethical considerations and administrative procedures.....95
11.1 Regulatory and ethical compliance.....95
11.2 Responsibilities of the investigator and IRB/IEC/REB.....95
11.3 Informed consent procedures.....95
11.4 Discontinuation of the study.....95
11.5 Publication of study protocol and results.....95
11.6 Study documentation, record keeping and retention of documents.....96
11.7 Confidentiality of study documents and patient records.....97
11.8 Audits and inspections.....97
11.9 Financial disclosures.....97
12 Protocol adherence.....97
12.1 Amendments to the protocol.....97
13 References (available upon request).....98
14 Appendices.....101
14.1 Appendix 1: Guidelines for Response, Duration of Overall Response, TTF, TTP, Progression-Free Survival and Overall Survival (version 3.2, based on RECIST 1.1).....101
14.1.1 Introduction.....102
14.1.2 Efficacy assessments.....102
14.1.3 Efficacy definitions.....111
14.1.4 Data handling and programming rules.....122
14.1.5 References (available upon request).....125

List of figures

Figure 1-1 Chemical structure of everolimus.....26
Figure 4-1 Study design.....42

List of tables

Table 1-1 Everolimus - drug substance.....26
Table 1-2 BOLERO-2 Study: most commonly reported adverse events (>/= 10% of patients treated with everolimus + exemestane).....32
Table 1-3 Any-grade adverse events with >/+ 10% incidence in Asian ABC patients treated with everolimus + exemestane.....33
Table 3-1 Objectives and related endpoints.....38

[redacted]

Table 6-1	Dose and treatment schedule	47
Table 6-2	Recommendation of everolimus dose reductions	48
Table 6-3	Criteria for interruption and re-initiation of everolimus/placebo treatment	49
Table 6-4	Management of non-infectious pneumonitis	53
Table 6-5	Action to be taken based on hepatitis B screening results	55
Table 6-6	Guidelines for management of hepatitis B	55
Table 6-7	Guidelines for management of hepatitis C flare	56
Table 6-8	Clinically relevant drug interactions: inducers, and inhibitors of isoenzyme CYP3A	60
Table 6-9	Clinically relevant drug interactions: substrates, inducers, inhibitors of PgP and PgP/CYP3A dual inhibitors	61
Table 6-10	Packaging and labeling	63
Table 6-11	Supply and storage of study treatments	63
Table 7-1	Visit evaluation schedule	65
Table 7-2	Imaging assessment collection plan	73
Table 7-3	ECOG Performance Status Scale	76
Table 7-4	Local clinical laboratory parameters collection plan	77
Table 7-5	PK blood collection schedule	80
Table 10-1	90% CIs for various observed hazard ratios	94
Table 14-1	Response criteria for target lesions	107
Table 14-2	Response criteria for non-target lesions	109
Table 14-3	Overall lesion response at each assessment	111
Table 14-4	Overall lesion response at each assessment: patients with non-target disease only	118
Table 14-5	Options for event dates used in PFS, TTP, duration of response	120

[redacted]

List of abbreviations

ABC	Advanced Breast Cancer
ADME	Absorption Distribution Metabolism and Excretion
AE	Adverse Event
AKT	Protein Kinase B
ALP	Alkaline phosphatase
ALT	Alanine aminotransferase/glutamic pyruvic transaminase/GPT
ANC	Absolutely Neutrophil Count
ASCO	American Society of Clinical Oncology
AST	Aspartate aminotransferase/glutamic oxaloacetic transaminase/GOT
AUC0-24h	Area Under the Curve 0-24 h
BAL	Bronchoalveolar lavage
BC	Breast Cancer
BRIC	Blinded independent review committee
BSA	Body Surface Area
BUN	Blood Urea Nitrogen
CBC	Cells Blood Count
CBR	Clinical Benefit Rate
CCG	Case Report/Record Form (CRF) Completion Guidelines
CERT	Center for Education and Research on Therapeutics
CI	Confidence Interval
CISH	Chromogenic in situ hybridization
CL	Clearance
Cmax	Maximum Concentration
CMV	Cytomegalovirus
CMO&PS	Chief Medical Office and Patient Safety
CNS	Central Nervous System
CPK	Creatine Phosphokinase
CR	Complete Response
CRF	Case Report/Record Form; the term CRF can be applied to either EDC or Paper
CRO	Contract Research Organization
CSR	Clinical study report
CT	Computed Tomography
CTCAE	Common Terminology Criteria for Adverse Events
CYP	Cytochrome P
DHEA	Dehydroepiandrosterone
DILI	Drug-induced liver injury
DLCO	Carbon monoxyde diffusing capacity
DLT	Dose limiting toxicity
DMC	Data Monitoring Committee
DNA	Deoxyribonucleic Acid
DOR	Duration of Response
DSM- IV	Diagnostic and Statistical Manual of Mental Disorders, 4th Edition
EBV	Epstein - Barr virus
EC	Ethic Committee
ECG	Electrocardiogram

[redacted]

ECHO	Echocardiogram
ECOG	Eastern Cooperative Oncology Group
Ecrf	Electronic Case Report/Record Form
EDC	Electronic Data Capture
EGF	Epidermal growth factor
EGFR	Epidermal growth factor receptor
EIAED	Enzyme-inducing anti-epileptic drug
EOT	End of Treatment
ER	Estrogen receptor
EU	European Union F
FAS	Full Analysis Set
FDG-PET	Fluorodeoxyglucose-Positron Emission Tomography
FFPE	Formalin-fixed paraffin embedded
FGF	Fibroblast growth factor
FISH	Fluorescence in situ Hybridization
Fm	The relative metabolic contribution
FPG	Fasting plasma glucose
FSH	Follicle-stimulating hormone
GABA	Gamma amino-butyric acid
GAD-7	General Anxiety Disorder Assessment
GCP	Good Clinical Practice
G-CSF	granulocyte colony stimulating factor
GGT	Gamma-glutamyltranspeptidase
GI	Gastrointestinal
GLP	Good Laboratory Practice
GM-CSF	Granulocyte macrophage colony-stimulating factor
HAV	Hepatitis A Virus
HbA_{1c}	Glycosylated Hemoglobin
HBsAg	Hepatitis B surface Antigen
HBV	Hepatitis B Virus
HCV	Hepatitis C Virus
hCG	human chorionic gonadotrophin
HDL	High density lipoprotein
HDPE	High Density Polyethylene
HER2	Human epidermal growth factor receptor 2
HER2-	Human epidermal growth factor receptor 2 negative
HER2+	Human epidermal growth factor receptor 2 positive
HIV	Human immunodeficiency virus
HLGT	High Level Group Term
HR	Hormone Receptor
HR	Heart Rate
HR+	Hormone sensitive
HSV	Herpes Simplex Virus
i.m.	Intra-muscularly
i.v.	intravenous(ly)
IB	Investigators Brochure
IC_{50}	Half maximal Inhibitory Concentration

[redacted]

ICF	Informed Consent Form
ICH	International Conference on Harmonization
IEC	Independent Ethics Committee
IGF-1	Insulin-like growth factor 1
IgG	Immunoglobulin G
IgM	Immunoglobulin M
IHC	Immunohistochemistry
INR	International Normalized Ratio
IRB	Institutional Review Board
IRT	Interactive Response Technology
LC-MS/MS	Liquid chromatography tandem mass spectrometry
LDH	Lactate dehydrogenase
LDL	Low density lipoprotein
LFT	Liver Function Tests
LMWH	Low molecular weight heparin
LVEF	Left Ventricular Ejection Fraction
MBC	Metastatic breast cancer
MedDRA	Medical Dictionary for Regulatory Activities
MGMT	The O-6-methylguanine-DNA methyltransferase
MRI	Magnetic Resonance Imaging
MTD	Maximum Tolerated Dose
mTOR	Mammalian target of rapamycin
MUGA	Multiple Gated acquisition scan
N	Sample size
NCCN	National Comprehensive Cancer Network
NCI CTC	National Cancer Institute Common Terminology Criteria
NEC	Not elsewhere classified
NTI	Narrow Therapeutic Index
OC	Oral contraception
ORR	Objective Response Rate
OS	Overall survival
PD	Progressive disease
PD	Pharmacodynamics
PFS	Progression-free survival
P-gR	Progesterone Receptor
PHI	Protected health information
PK	Pharmacokinetics
PLT	Platelets
pNET	Pancreatic Neuroendocrine Tumor
PoC	Proof of Concept
PPS	Per Protocol Set
PR	Partial Response
PT	Prothrombin time
PTEN	Phosphatase and tensin homolog
QTcF	Q-T interval in the ECG (corrected according to the formula of Fridericia)
RAP	Report Analysis Plan
RBC	Round Blood Cells

[redacted]

RCC	Renal cell carcinoma
REB	Research Ethics Board
RECIST	Response Evaluation Criteria In Solid Tumors
RP2D	Recommended Phase II dose
SAE	Serious Adverse Event
SC	Steering Committee
SD	Stable disease
SoC	Standard of Care Treatment
TBL	Total bilirubin
TdP	Torsades de Pointes
Tmax	The time at which the maximum observed concentration (Cmax) occurs
TNBC	Triple Negative Breast Cancer
TSH	Thyroid stimulating hormone
TTP	Time to Progression
UDPGA	Uridine 5'-diphospho-glucuronic acid
UGT1A4	UDP-glucuronosyltransferase 1 family, polypeptide A4
UNK	Unknown
ULN	Upper Limit of Normal
VEGF	Vascular endothelial growth factor
Vss	Volume of distribution at steady state
WBC	White Blood Cell
WHO	World Health Organization

[redacted]

Glossary of terms

Assessment	A procedure used to generate data required by the study
Biologic Samples	A biological specimen including, for example, blood (plasma, serum), saliva, tissue, urine, stool, etc. taken from a study subject or study patient
Control drug	A study treatment used as a comparator to reduce assessment bias, preserve blinding of investigational drug, assess internal study validity, and/or evaluate comparative effects of the investigational drug
Cycles	Number and timing or recommended repetitions of therapy are usually expressed as number of days (e.g.: q28 days)
Dose level	The dose of drug given to the patient (total daily or weekly etc.)
Enrollment	Point/time of patient entry into the study; the point at which informed consent must be obtained (i.e. prior to starting any of the procedures described in the protocol)
Investigational drug	The study treatment whose properties are being tested in the study; this definition is consistent with US CFR 21 Section 312.3 and is synonymous with “investigational new drug.”
Investigational treatment	Drug whose properties are being tested in the study as well as their associated placebo and active treatment controls (when applicable). This also includes approved drugs used outside of their indication/approved dosage, or that are tested in a fixed combination. Investigational treatment generally does not include other study treatments administered as concomitant background therapy required or allowed by the protocol when used in within approved indication/dosage
Medication number	A unique identifier on the label of each study treatment package which is linked to one of the treatment groups of a study
Other study treatment	Any drug administered to the patient as part of the required study procedures that was not included in the investigational treatment
Subject Number	A unique identifying number assigned to each patient/subject/healthy volunteer who enrolls in the study
Randomization number	A unique treatment identification code assigned to each randomized patient, corresponding to a specific treatment arm assignment
Stage related to study timeline	A major subdivision of the study timeline; begins and ends with major study milestones such as enrollment, randomization, completion of treatment, etc.
Stage in cancer	The extent of a cancer in the body. Staging is usually based on the size of the tumor, whether lymph nodes contain cancer, and whether the cancer has spread from the original site to other parts of the body
Stop study participation	Point/time at which the patient came in for a final evaluation visit or when study treatment was discontinued whichever is later
Study treatment	Includes any drug or combination of drugs in any study arm administered to the patient (subject) as part of the required study procedures, including placebo and active drug run-ins.<br>In specific examples, it is important to judge investigational treatment component relationship relative to a study treatment combination; study treatment in this case refers to the investigational and non-investigational treatments in combination.
Study treatment discontinuation	Point/time when patient permanently stops taking study treatment for any reason
Variable	Identifier used in the data analysis; derived directly or indirectly from data collected using specified assessments at specified time points
Withdrawal of consent	Withdrawal of consent occurs only when a patient does not want to participate in the study any longer, and does not want any further visits or assessments, and does not want any further study related contact

[redacted]

Protocol summary

**Title**	A randomized, double-blind, placebo controlled, phase II study of Everolimus in combination with Exemestane in the treatment of Chinese postmenopausal women with estrogen receptor positive, HER-2 negative, locally advanced, recurrent, or metastatic breast cancer after recurrence or progression on prior letrozole or anastrozole
**Brief title**	A study of everolimus plus exemestane in Chinese postmenopausal women with estrogen receptor positive, locally advanced, recurrent, or metastatic breast cancer after recurrence or progression on non-steroidal aromatase inhibitor
**Sponsor and Clinical Phase**	Novartis<br>Phase II
**Investigation type**	Drug
**Study type**	Interventional
**Purpose and rationale**	This study aims at evaluating the safety and efficacy of everolimus plus exemestane in Chinese postmenopausal women with ER+ HER2- ABC after recurrence or progression on letrozole or anastrozole.<br>The rationale of this study is based on the following:<br>• Proven everolimus activity in breast cancer in combination with exemestane<br>• Efficacy and manageable safety profile of everolimus in combination with exemestane in the Asian subpopulation of BOLERO-2
**Primary Objective**	To assess the progression-free survival (PFS) as determined by investigator assessment of everolimus and exemestane and of exemestane and placebo
**Secondary Objectives**	• To assess PFS in the two treatment arms as determined by a Blinded Independent Review Committee (BIRC).<br>• To assess overall survival (OS) in the two treatment arms<br>• To assess the following efficacy endpoints in the two treatment arms based on both local investigator's assessment and BIRC<br>• Overall response rate (ORR)<br>• Clinical benefit rate (CBR)<br>• Time to response and duration of response<br>• To assess time to deterioration of ECOG Performance Status<br>• To characterize the safety and tolerability of everolimus and exemestane versus placebo and exemestane<br>• Characterize the pharmacokinetics of everolimus (C_{min}, C_{2h}) when administered in combination with exemestane<br>• Compare the two treatment arms with respect to pre-dose concentration (C_{min}) and concentration at 2 hours post dose (C_{2h}) of exemestane and to compare the two treatment arms with respect to estradiol (E2) changes from baseline.
**Study design**	Refer to Section 4.<br>This is a multicenter, double-blind, randomized, placebo-controlled, phase II study evaluating treatment with everolimus (10 mg daily) versus placebo in combination with exemestane (25 mg daily) in Chinese postmenopausal women with locally advanced, recurrent or metastatic, ER positive, HER-2 negative breast cancer refractory to non-steroidal aromatase inhibitors.<br>Approximately 160 patients will be randomized in 1:1 ratio to receive either everolimus or matching placebo in a blinded manner in addition to open label exemestane).<br>Screening phase:<br>Written informed consent must be obtained before any study specific medical procedures are performed. The investigator or his/her authorized designee will assign a unique number to patients being considered for the study.<br>After the patient signs the informed consent and prior to the first dose of study

[redacted]

	treatment, patients will be screened in IRT. All inclusion/exclusion criteria must be verified. Patients who do not meet the eligibility criteria will not receive study treatment.<br>All screening assessments to confirm eligibility must be performed within maximum 21 days prior to the first dose of study treatment (Table 7-1 and Section 7.1).<br>Randomized treatment phase:<br>All eligible patients will receive study treatment on Cycle 1 Day 1. These patients must be randomized into one of the two treatment arms (everolimus plus exemestane arm or placebo plus exemestane arm) within 7 days before Cycle 1 Day 1. Randomization will be stratified according to the presence of visceral disease and sensitivity to prior hormonal therapy status. Visceral refers to lung, liver, brain, pleural and peritoneal involvement. Sensitivity to prior hormonal therapy is defined as either (1) documented clinical benefit (complete response, partial response, stable disease ≥ 24 weeks) to at least one prior hormonal therapy in the advanced setting or (2) at least 24 months of adjuvant hormonal therapy prior to recurrence.<br>Randomized patients will start the study treatment at Cycle 1 Day 1. Patients will receive everolimus/placebo (10 mg daily oral tablets) in addition to exemestane (25 mg daily oral tablets) continuously. Dose adjustment (reduction, interruption) according to safety findings will be allowed.<br>Patients will receive treatment until disease progression (assessed by RECIST 1.1), unacceptable toxicity, death or discontinuation from treatment for any other reason. Tumor assessments will be performed every 8 weeks after randomization date until radiological progression per local assessment. The frequency of tumor assessment will be changed to every 12 weeks after primary analysis data cut-off and as clinically indicated, until disease progression.<br>Post-treatment follow-up phase:<br>After end of treatment visit, all patients will be followed up for safety up to 30 days after last dose of study treatment (exemestane and/or everolimus/placebo).<br>All patients who discontinue from study treatment due to disease progression must have their progression clearly documented according to the criteria specified in RECIST 1.1.<br>If a patient permanently discontinues study treatment for reasons other than disease progression, death, lost to follow-up, or withdrawal of consent to efficacy follow-up then tumor assessments should continue to be performed per assessment schedule until disease progression, death, lost to follow-up or withdrawal of consent for efficacy follow-up.<br>Antineoplastic therapy regimen received between EOT and progression will also be collected as well as the first regimen received after progression.<br>Survival follow-up phase<br>All patients will be followed for survival status at least every 3 months after randomized treatment phase or post-treatment follow-up phase unless they discontinued due to death, consent withdrawal or lost to follow-up. Survival information can be obtained via phone and information will be documented in the source documents and eCRF. Additional survival assessments may be performed more frequently if a survival update is required for reporting the results or to meet safety or regulatory needs.
**Population**	Refer to Section 5.1.<br>Chinese Postmenopausal women with ER+ HER2- locally advanced, recurrent, or metastatic breast cancer after recurrence or progression on prior letrozole or anastrozole.<br>Recurrence or progression on prior NSAI is defined as:<br>• Recurrence while on, or within one year (12 months) of end of adjuvant treatment with letrozole or anastrozole<br>• Progression while on or within one month (30 days) of the end of prior treatment with letrozole or anastrozole<br>Except for prior use of exemestane, mTOR inhibitors, PI3K inhibitors, AKT

[redacted]

	inhibitors, there are no restrictions as to the last anticancer treatment prior to randomization. Patients must have a documented evidence of recurrence or progression on last therapy prior to randomization. No more than one line of prior chemotherapy for advanced disease is allowed.
**Inclusion criteria**	Refer to Section 5.2.<br>Patients eligible for inclusion in this study have to meet **all** of the following criteria:<br>1. Women (18 years or older) of Asian ethnicity with locally advanced, recurrent, or metastatic breast cancer. Locally advanced breast cancer must not be amenable to curative treatment by surgery or radiotherapy.<br>2. Histological or cytological confirmation of estrogen-receptor positive (ER+) breast cancer<br>3. Postmenopausal women. Postmenopausal status is defined either by:<br>• Prior bilateral oophorectomy<br>• Or age ≥ 60<br>• Or age < 60 and amenorrhea for 12 or more months (in the absence of chemotherapy, tamoxifen, toremifen, or ovarian suppression), and FSH and estradiol in the postmenopausal range (serum FSH > 40 mIU/mL and estradiol <20 pg/mL or according to the postmenopausal range defined by local laboratory).<br>For women with therapy-induced amenorrhea, oophorectomy or serial measurements of FSH and/or estradiol are needed to ensure postmenopausal status.<br>**Note:** Ovarian radiation or treatment with a luteinizing hormone-releasing hormone (LH- RH) agonist (goserelin acetate or leuprolide acetate) is not permitted for induction of ovarian suppression.<br>4. Recurrence or progression on prior NSAI is defined as:<br>• Recurrence while on, or within one year (12 months) of end of adjuvant treatment with letrozole or anastrozole<br>OR<br>• Progression while on or within one month (30 days) of the end of prior treatment with letrozole or anastrozole<br>Notes:<br>• Letrozole or anastrozole do not have to be the last treatment prior to the enrollment in the study<br>5. Patients must have improved to grade 1 or better from any adverse events (with exception of alopecia) related to previous therapy prior to enrollment<br>6. Have radiological or objective evidence of recurrence or progression on or after the last systemic therapy prior to enrollment<br>7. Patient must have as per RECIST 1.1<br>• measurable disease<br>or<br>• non-measurable lytic or mixed (lytic + blastic) bone lesions in the absence of measurable disease.<br>**Note:** Measurable lesions include lytic or mixed (lytic + blastic) bone lesions, with an identifiable soft tissue component that meets the measurability criteria per RECIST 1.1.<br>Patients with only non-measurable lesions (e.g. pleural effusion, ascites) and no lytic or a mix of lytic and blastic bone lesions are not eligible.<br>8. Patient is able to swallow and retain oral medication.<br>9. Patient must meet the following laboratory values at the screening visit:<br>• Absolute Neutrophil Count (ANC) ≥ 1.5 × 10^9/L<br>• Platelets ≥ 100 × 10^9/L<br>• Hemoglobin (Hgb) ≥ 9 g/dL<br>• INR≤2<br>• Serum creatinine ≤ 1.5 × ULN

[redacted]

	• Total bilirubin ≤ 1.5 × ULN (≤ 3 × ULN for patients known to have Gilbert Syndrome)<br>• Aspartate transaminase (AST) ≤ 2.5 × ULN, except for patients with liver metastasis, who may only be included if AST ≤ 5.0 × ULN<br>• Alanine transaminase (ALT) ≤ 2.5 × ULN, except for patients with liver metastasis, who may only be included if ALT ≤ 5.0 × ULN<br>• Fasting serum cholesterol ≤ 300 mg/dl or 7.75 mmol/L and fasting triglycerides ≤ 2.5 × ULN. In case one or both of these thresholds are exceeded, the patient can only be included after initiation of statin therapy and when the above mentioned values have been achieved<br>10. Patient has a WHO performance status ≤2<br>11. Written informed consent must be obtained prior to any screening procedures.
**Exclusion criteria**	Refer to Section 5.3.<br>Patients eligible for this study must not meet **any** of the following criteria:<br>1. HER2-overexpressing patients by local laboratory testing (IHC 3+ staining or in situ hybridization positive), based on the most recent test. Note: Patients with IHC 2+ must have a negative in situ hybridization test.<br>2. Patients who received more than one chemotherapy line for ABC<br>Note: A chemotherapy line in advanced disease is an anticancer regimen that contains at least one chemotherapy agent and is given for 21 days or longer. If a cytotoxic chemotherapy regimen was discontinued for a reason other than disease progression and lasted less than 21 days, then this regimen does not count as a "prior line of chemotherapy". Chemotherapy regimens composed of more than one drug are considered as one line of therapy.<br>3. Patients with symptomatic visceral disease and candidate to chemotherapy<br>4. Patients with only non-measurable lesions other than lytic or mixed (lytic and blastic) bone metastasis (e.g. pleural effusion, ascites etc.)<br>5. Previous treatment with exemestane, mTOR inhibitors, PI3K inhibitors, AKT inhibitors.<br>6. Known hypersensitivity to mTOR inhibitors, e.g. sirolimus (rapamycin)<br>7. Patients who have a history of another primary malignancy, with the exceptions of non-melanoma skin cancer, and carcinoma *in situ* of the cervix, uteri, or breast from which have been disease free for ≥3 years<br>8. Radiotherapy within four weeks prior to enrollment in the study except in case of localized palliative radiotherapy (for analgesic purpose) or for lytic lesions at risk of fracture which was completed at least two weeks prior to registration in the current study. Patients must have recovered from radiotherapy toxicities.<br>9. Currently receiving any hormone replacement therapy, unless discontinued prior to registration in the current study.<br>10. Patients with central nervous system (CNS) involvement unless they meet ALL of the following criteria:<br>• At least 4 weeks from prior therapy completion (including radiation and/or surgery) to starting study treatment.<br>• Clinically stable CNS lesions at the time of screening which are untreated or without evidence of progression for at least 4 weeks after treatment, as determined by clinical examination and brain imaging (MRI or CT). Patients must have not been treated with steroids for brain metastases during the screening period<br>11. Patients receiving concomitant immunosuppressive agents or chronic corticosteroids use at the time of study entry except topical applications, inhaled sprays, eye drops or local injections.<br>12. Bilateral diffuse lymphangitic carcinomatosis<br>13. Severely impaired lung function as defined as spirometry and DLCO that is 50% or less of the normal predicted value and/or O2 saturation that is 88% or less at rest on room air<br>14. Patients who have serious liver disease such as cirrhosis, decompensated liver

[redacted]

	disease, and acute or currently active hepatitis (i.e. quantifiable HBV-DNA and/or positive HbsAg, quantifiable HCV-RNA)<br>15. Patients with a known history of HIV seropositivity. Screening for HIV infection at baseline is not required.<br>16. Active, bleeding diathesis, or on oral anti-vitamin K medication (except low dose warfarin, LMWH and acetylsalicylic acid or equivalent, as long as the INR is ≤ 2).<br>17. Impairment of gastrointestinal (GI) function or GI disease that may significantly alter the absorption of oral everolimus.<br>18. Uncontrolled diabetes mellitus as defined by HbA1c >7% despite adequate therapy. Patients with a known history of impaired fasting glucose or diabetes mellitus (DM) are eligible, however blood glucose and antidiabetic treatment must be monitored closely throughout the trial and adjusted as necessary;<br>19. Active ulceration of the upper gastrointestinal tract.<br>20. Any severe and / or uncontrolled medical conditions such as<br>a. unstable angina pectoris, symptomatic congestive heart failure, myocardial infarction ≤6 months prior to randomization, serious uncontrolled cardiac arrhythmia,<br>b. active or uncontrolled severe infection,<br>21. Patients being treated with drugs recognized as being strong inhibitors or inducers of the isoenzyme CYP3A4 (Rifabutin, Rifampicin, Clarithromycin, Ketoconazole, Itraconazole, Voriconazole, Ritonavir, Telithromycin) continuously for at least 7 days during any time period in the last 2 weeks prior to registration in the current study<br>22. Patients who have received live attenuated vaccines within 1 week of start of study drug and during the study. Patient should also avoid close contact with others who have received live attenuated vaccines. Examples of live attenuated vaccines include intranasal influenza, measles, mumps, rubella, oral polio, BCG, yellow fever, varicella and TY21a typhoid vaccines;<br>23. History of noncompliance to medical regimens<br>24. Patients unwilling to or unable to comply with the protocol<br>25. Patients who are currently part of or have participated in any clinical investigation with an investigational drug within 1 month prior to dosing;
**Investigational and reference therapy**	Refer to Section 6.1.<br>Study treatment is defined as everolimus +exemestane or placebo + exemestane (10 mg/day + 25 mg/day)
**Efficacy assessments**	Refer to Section 7.2.<br>Imaging assessments as described in Table 7-2 should be performed at the time-points specified using the same imaging modality used at baseline, irrespective of study treatment interruption or actual dosing (see Table 7-1). Imaging assessments for response evaluation will be performed every 8 weeks after randomization. Tumor assessments are to be performed every 12 weeks after primary analysis data cut-off and as clinically indicated, until disease progression.<br>Additional imaging assessments may be performed at any time during the study at the investigator's discretion to support the efficacy evaluations for a subject, as necessary. Clinical suspicion of disease progression at any time requires a physical examination and imaging assessments to be performed promptly rather than waiting for the next scheduled imaging assessment.<br>Each lesion that is measured at baseline must be measured by the same method (either same imaging method or by photography, including a metric ruler) and when possible, the same local radiologist/physician throughout the study so that the comparison is consistent. If an off-schedule imaging assessment is performed because progression is suspected, subsequent imaging assessments should be performed in accordance with the original imaging schedule.<br>Combined PET/CT may be used only if the CT is of similar diagnostic quality as a CT performed without PET, including the utilization of IV contrast media. At the discretion of the Investigators, FDG-PET scans may be performed to document

[redacted]

	progressive disease per RECIST 1.1 (Appendix 1).<br>Imaging data will be centrally collected and checked for quality by an imaging Contract Research Organization (CRO) designated by Novartis.
**Safety assessments**	Refer to Section 8.<br>Safety assessments will consist of monitoring and recording all adverse events (AEs), including serious adverse events (SAE), the regular monitoring of hematology, serum chemistry, routine monitoring of vital signs (heart rate, blood pressure, and body temperature), weight, ECOG performance status, chest CT scans, and physical condition. Toxicity will be assessed using the NCI-CTC Common Terminology Criteria for Adverse Events, version 4.03: (CTCAE v4.03).
**Data analysis**	Refer to Section 10.<br>The primary efficacy population is defined as the Full Analysis Set (FAS), which consists of all randomized patients. All primary efficacy analyses will be evaluated based on data from this population on an intent-to-treat (ITT) basis according to the treatment group and strata they were assigned to at randomization.<br>The safety population will consist of all patients who received at least one dose of the study treatment defined as either everolimus + exemestane or matching placebo + exemestane. Patients will be analyzed according to treatment actually received.<br>**Data analysis for the primary objective:**<br>The primary objective in this study is to assess PFS on the combination treatment of everolimus + exemestane and placebo + exemestane in Chinese postmenopausal women with estrogen receptor positive, locally advanced, recurrent, or metastatic breast cancer after recurrence or progression on prior letrozole or anastrozole.<br>The primary analysis of PFS will be performed based on the local investigator's assessment. The distribution of PFS will be estimated using the Kaplan-Meier method. The median PFS along with 90% CI will be presented by treatment arm. The Kaplan-Meier curves will be displayed and the number of patients at risk at certain time points will be shown on the plot. A Cox regression model stratified by randomization stratification factors will be used to estimate the hazard ratio (HR) of PFS, along with 90% CI. The primary analysis for PFS will be repeated based on central review data as a supportive analysis.<br>**Data analysis for the secondary objectives:**<br>The secondaryobjectives in this study are to assess the two treatment groups with respect to PFS determined by BIRC (Central review should stop after the number of events is reached for primary PFS analysis), OS, ORR, deterioration in the ECOG PS, safety, and CBR. Additionally, time to response (TTR) and duration of response (DOR) will be summarized for both treatment groups.<br>Overall survival will be estimated using the Kaplan-Meier method. The median OS along with 90% confidence intervals will be presented by two treatment groups, along with proportion of patients alive at 3, 6, 9, 12 and 18 months and the associated 90% confidence intervals. The stratified Cox regression will be used to estimate the HR of OS, along with 90% confidence interval.<br>ORR and CBR will be estimated and the exact binomial 90% CI will be reported by treatment arm. The above analyses will be performed on local assessment and also be repeated using data from central review as supportive analysis.<br>TTR and DOR will be summarized using descriptive statistics. The analysis will be performed based on investigator assessment and also repeated using data from central review as supportive analyses.<br>An analysis of the time to definitive deterioration of the ECOG PS by one category of the score from baseline will be performed. Kaplan-Meier method will be used to estimate the distribution function of time to definitive worsening. The estimated treatment-specific median time to definitive worsening will be presented along with a 90% confidence interval for the two treatment arms.<br>The assessment of safety will be based mainly on the frequency of adverse events and on the number of laboratory values that fall outside of pre-determined ranges. Other safety data (e.g., electrocardiogram, vital signs) will be considered as

[redacted]

	appropriate.
**Key words**	Chinese postmenopausal woman, Advanced breast cancer, Endocrine therapy, Aromatase inhibitors

[redacted]

Amendment 1 (23-May-2017)

Enrollment to Study CRAD001Y2202 is expected to commence in July 2017. Protocol Version 00 was previously approved by pOPRC on 31 August 2016.

Amendment rationale

The main purposes of this amendment are to:

1. Revise the PK collection schedule to better clarify time points of PK collection during cycle 1 week 4, as well the definition of the steady state related to vomiting event prior to the pharmacokinetic blood sampling.
2. DMC is not applicable for this study because of the following reasons:
    a. No interim analysis is planned for this study. According to Novartis internal guidelines, data monitoring committees are to be considered for studies with at least one interim analysis of safety and/or efficacy data
    b. The safety profile of everolimus is well established, including the population of Chinese patients with breast cancer. The Steering Committee will review the blinded pooled safety data approximately every 6 months
    c. The everolimus PK exposure in Chinese patients is comparable to Caucasian patients
3. To clarify the process and eligibility for re-screening
4. Update Guidelines for Response, Duration of Overall Response, TTF, TTP, Progression-Free Survival and Overall Survival (based on RECIST 1.1) to version 3.2
5. To be consistent with the updated RAD safety profile, the inclusion/exclusion criteria section is updated accordingly.
6. To be consistent with the updated RAD safety profile, the dose modification section is updated accordingly.
7. To update various sections of the protocol to ensure consistency with the most recent standard protocol for oncology study. Namely, the visit schedule and the definition of the end of treatment, end of post-treatment phase and survival follow-up were clarified. The AE section is now including collection of grade 5 AE's. Also typographical errors were corrected throughout the protocol.

Changes to the protocol

The changes being made to the protocol due to this amendment are incorporated in the following sections:

- Author list: updated authors lists: [redacted].
- List of abbreviations: deleted DS&E and added CMO&PS per the latest protocol template.
- Protocol summary: revised protocol summary according to changes in main contents.
- Section 1.1.2: Added "Ribociclib, another CDK4/6 inhibitor, combined with letrozole also gained FDA approval in March 2017 as first line treatment post-menopausal for women with HR-positive, HER2-negative advanced breast cancer."
- Section 2.1: Added "Breast cancer" to NCCN guidelines

[redacted]

- Section 4.1: Patient number was replaced by Subject number
- Section 4.1: Survival follow-up section was clarified: All patients will be followed for survival status at least every 3 months after randomized treatment phase or post-treatment follow-up phase unless they discontinued due to death, consent withdrawal or lost to follow-up.
- Figure 4-1: Study design figure clarifies the post –treatment follow-up phase and the survival follow-up phase.
- Section 4.3: deleted “If the primary analysis of PFS does not demonstrate treatment benefit, the follow up for OS will end”
- Section 4.4: In case of early study termination, the completion of end of post treatment follow up visit was added
- Section 5.2 inclusion criteria #9, INR≤2 was added
- Section 5.2 inclusion criteria #10, changed to Patient has a WHO performance status ≤2;
- Section 5.3:
  - Exclusion criteria related to patients who have a history of another primary malignancy, period of disease free > 5 years to 3 years.
  - Criteria of patients with CNS involvement were updated per the latest protocol template.
  - Exclusion criteria of patients with uncontrolled diabetes mellitus was added
  - Exclusion criteria of patient with uncontrolled medical condition was updated
  - Exclusion criteria of patients who have received live attenuated vaccines within a week of study start was added
  - Exclusion criteria of patients having participated in clinical trials was added
- Section 6.1.5 : in the treatment duration section, Addition of end of treatment reasons: Protocol deviation, Study termination by sponsor, Technical problem
- Section 6.3.1, Table 6-3 updated:
  - for hematological toxicity, modification of guidance for thrombocytopenia grade 2 and detailed guidance for febrile neutropenia grade 3 and grade 4
  - for other toxicities (non-hematological), addition of detailed guidance for grade 1 to grade 4
- Section 6.3.2.1 : addition of the section related to management of infection during treatment with everolimus
- Section 6.3.2.3: Added “especially during the first 8 weeks of treatment (majority of stomatitis events occur within first 8 weeks of treatment).”
- Section 6.4.3:
  - Tables 6-8 and 6-9 were updated
  - Instructions regarding the use of vaccination during the study has been added
- Section 6.5.1: Patient number was replaced by Subject number.
- Table 7-1 Visit evaluation schedule:

[redacted]

  - Addition of the visit name related to Post-treatment follow-up phase and survival follow-up phase
  - Relevant medical history/current medical conditions was replaced by medical history
  - PK blood sampling visit was added for cycle 1.
  - Removed "X" of PK blood sampling from the column of "Every 2 cycles after C1D1".
  - Removed visit 4 in footnotes 1 and 1a, respectively, as visit number is not applicable in this protocol.
  - Removed "X" for performance status during post-treatment follow-up phase as not performed during the post-treatment follow-up phase
  - Clarify in the tumor evaluation, the frequency of tumor evaluation in the post-treatment follow-up phase
- Section 7.1.1.1: Process and eligibility for re-screening was included: patients, who met all inclusion exclusion criteria, however were not able to be randomized within the screening window due to administrative issues, will be allowed to be re-screened for randomization.
- Section 7.1.2.3: update of "HER status" to "HER2+ status". Replacement of "date of birth" by "year of birth".
- Section 7.1.4: Removed "study" in the sentence "Patients who discontinue study treatment should undergo an end of study treatment visit".
- Section 7.1.6.1: Removed the sentence "the investigator or his/her designee will complete a Study Evaluation Completion CRF page". Replace the "Study Evaluation Completion eCRF" by the "appropriate disposition CRF".
- Section 7.1.6.3: Addition of the paragraph related to Survival follow-up "All patients who discontinued treatment phase or the post-treatment follow-up phase for reasons other than death, consent withdrawal, will be followed for survival status at least every 3 and until end of study. Survival information can be obtained via phone and information will be documented in the source documents and eCRF. Additional survival assessments may be performed more frequently if a survival update is required for reporting the results or to meet safety or regulatory needs"
- Section 7.2.1 – Efficacy assessments: Novartis guideline version 3.1 was updated to 3.2.
- Section 7.2.1 – Efficacy assessments: Added "Central review should stop after the number of events is reached for Primary PFS analysis."
- Section 7.2.1: Removal of " if clinically indicated " for Brain CT or MRI and Whole body bone scan
- Section 7.2.2.4: Performance status, removal of " even if study treatment is being interrupted"
- Section 7.2.3: in the section " if the patient vomits within the first 4 hours", "or the 2 days" was replaced by "or the day before", and removal of the "Vomiting Log eCRF"
- Section 7.2.3.1 Pharmacokinetic blood sample collection and handling:
  - Volumes of PK blood sampling were revised to be consistent with Table 7-5
  - Deleted visit 4 and added any time during week 4

[redacted]

- Section 7.2.2.5.3: “ New or worsening” was replaced by “New (including new or worsened)”
- Table 7-5 PK Blood Collection Schedule:
  - “Visit” was changed to “Visit Name”;” ; “4” was changed to “Cycle1/Week 4” in this column.
  - “PK Collection number” was changed to “Dosed Reference ID”.”
- Section 8.1.1 Definition and reporting:
  - AESIs were updated according to current Case Retrieval Strategy
  - AEs were updated per the latest protocol template (include collection of grade 5)
  - “Action taken with respect to study or investigational treatment” was updated per the latest oncology standards.
- Section 8.2.2 Reporting: DS&E was changed to Chief Medical Office and Patient Safety per the protocol template.
- Section 8.6: Changed to: “Not Applicable”.
- Section 8.7: Added “The SC will review blinded pooled safety data approximately every 6 months (after the first randomized patient has started study treatment) and provide recommendations to the sponsor regarding the conduction of the study.”
- Section 10.1.2 Safety set: “Note: the statement that a patient had no adverse events constitutes a safety assessment.” was removed.
- Section 10.2: second paragraph, removal of the “Relevant”, in the sentence “ Relevant medical history (…) will be summarized”.
- Section 10.5.1 Secondary efficacy objectives: “safety” was removed from the list of secondary efficacy objectives.
- Section 10.5.1 Secondary efficacy objectives: Added “(Central review should stop after the number of events is reached for Primary PFS analysis)”
- Section 10.5.1.6 ECOG Performance Status: The last sentence was changed to: “The estimated treatment-specific median times to definitive worsening will be presented along with a 90% confidence interval for the two treatment arms.”
- Section 10.5.2.2 Adverse events: AESIs were updated according to current Case Retrieval Strategy
- Section 10.5.2.3 Laboratory abnormalities: “For laboratory tests where grades are not defined by CTCAE v4.03, results will be categorized as low/normal/high based on laboratory normal ranges.” was added per the latest protocol template.
- Section 10.5.3 Pharmacokinetics: “(visit 4)” was deleted.
- Section 10.5.3 Pharmacokinetics : the sentence “no vomiting within 4 hours after any of the previous 2 doses” was replaced by “no vomiting within 4 hours following study drug administration on the day of or the day before pharmacokinetic blood sampling”; and the sentence “no vomiting within 4 hours after any of the previous 2 doses and the dose prior to the C_{2h} sample” was replaced by “no vomiting within 4 hours following study drug administration on the day of or the day before pharmacokinetic blood sampling”
- Section 14.1 Appendix 1: Novartis guideline version 3.1 was updated to 3.2.

[redacted]

1 Background

1.1 Overview of disease pathogenesis, epidemiology and current treatment

1.1.1 Epidemiology of breast cancer

Breast cancer is the most frequently diagnosed cancer in women accounting for 23% (1.38 million) of all new cancer cases and is the leading cause of cancer related deaths in females worldwide causing more than 400,000 deaths yearly. The incidence rates in Asia is lower than in western countries (Jemal et al 2011) accounting for 21.6 cases per 100,000 woman in China (Globocan 2008). It, however, has increased dramatically over past 30 years and this trend is expected to continue in the next 20 years due to shifts in the reproductive and lifestyle risk factors (Linos et al 2008). The presence of hormone receptor (HR) [estrogen receptor (ER) and/or progesterone receptor (PgR)] is one of the most important prognostic and predictive factors detected in approximately 70% of all invasive breast cancers. Endocrine therapy is the core treatment modality in patients with HR+ advanced breast cancer (ABC).

1.1.2 Treatment options for hormone receptor positive advanced breast cancer

Endocrine therapy options for postmenopausal women with estrogen receptor positive (ER+), ABC include selective nonsteroidal aromatase inhibitors (NSAI; anastrozole and letrozole), steroidal aromatase inhibitors (exemestane), estrogen receptor antagonist (fulvestrant), and selective estrogen receptor modulator (SERM; tamoxifen). Blocking of estrogenic signaling with tamoxifen has been the main approach in treatment for ER+ breast cancer for over 30 years. Tamoxifen is indicated for the treatment across the whole continuum of breast cancer, ranging from risk reduction in women with high risk of developing breast cancer to treatment in multiple lines of metastatic disease. Aromatase inhibitors (AI) reduce peripheral estrogen synthesis by blocking the conversion of androgens to estrogens, which is the primary way estrogens are produced in postmenopausal women. Aromatase inhibitors are generally prescribed as the first line of therapy for the treatment of postmenopausal women with ER+ breast cancer (Beslija 2009, Cardoso 2011, [NCCN 2011.2]).

Inhibition of CDK 4/6 prevents DNA replication by prohibiting progression from G1 to S phase during cell division. Blocking this mechanism prevents tumor cell proliferation through control of the cell cycle. Palbociclib is the first CDK4/6 inhibitor which was approved by FDA in February 2015, in combination with letrozole as initial endocrine based therapy for the treatment of postmenopausal women with HR-positive, HER2-negative advanced breast cancer. On February 19, 2016, the FDA also approved palbociclib in combination with fulvestrant for the treatment of women with hormone receptor (HR)-positive, human epidermal growth factor receptor 2 (HER2)-negative advanced or metastatic breast cancer with disease progression following endocrine therapy. Ribociclib, another CDK4/6 inhitor, combined with letrozole also gained FDA approval in March 2017 as first line treatment post-menopausal for women with HR-positive, HER2-negative advanced breast cancer.

[redacted]

Despite a broad spectrum of available options of endocrine therapy for the patients with ER+ ABC, all patients will eventually develop resistance to initial treatment. An emerging mechanism of endocrine resistance is aberrant signaling via the phosphatidylinositol 3-kinase (PI3K)/Akt/mammalian target of rapamycin (mTOR) signaling pathway (Burstein 2011, Johnston 2006, Schiff 2004). Also, hyperactivation of the PI3K/mTOR pathway is observed in endocrine-resistant breast cancer cells, and treatment with mTOR inhibitors, including rapamycin analogs, reverses this resistance (e.g. Miller 2010). In addition, growing evidence supports a close interaction of the mTOR pathway with ER signaling. A substrate of mTOR Complex 1 (mTORC1), S6 kinase-1 (S6K1), phosphorylates the activation domain AF-1 of the ER, responsible for ligand-independent receptor activation (Yamnik 2009; Yamnik 2010).

Everolimus is a rapamycin derivative that inhibits mTOR through allosteric binding to mTORC1 but not mTORC2 (Efeyan and Sabatini 2010). In the human study, everolimus monotherapy demonstrated clinical activity in patients with ABC who had mostly ER+ tumors and had received previous endocrine therapy (Ellard et al 2009). In this trial, 19 of the 49 patients enrolled were ER-positive/human epidermal growth factor receptor 2 (HER2)-negative; Median PFS for these 19 patients was 3.5 months (95% C.I.: 1.9 – 5.5 months, data source: NCI-Canada).

More recently, the combination of everolimus with exemestane showed significant improvement in efficacy, in terms of PFS, response rate, and clinical benefit rate, relative to exemestane monotherapy (Baselga et al 2011). The median PFS by local assessment was 7.8 months for everolimus + exemestane versus 3.2 months for exemestane (HR = 0.45; 95% CI: 0.38-0.54; P<.0001). Overall response rate (ORR) (12.6% vs 1.7%; P<.0001) and CBR (51.3% vs 26.4%; P<.0001) were superior in the everolimus + exemestane arm versus exemestane + placebo. Analyses by central assessment showed a median PFS of 11.0 months with everolimus versus 4.1 months with placebo (HR = 0.38; 95% CI: 0.31 – 0.48; P <.0001) confirming the results of the primary PFS analysis (Yardley et al 2013). At the time of the final OS analysis, the median duration of OS was 30.98 months versus 26.55 months for the everolimus + exemestane arm and the placebo + exemestane arm, respectively [HR= 0.89(95% CI: 0.73 to 1.10; p=0.1426)] (Piccart et al 2014). The combination of everolimus and exemestane has received a marketing authorization in the USA, EU and many other countries based on the results of this large randomized phase III study.

1.1.3 Role of mammalian target of rapamycin in ER+ ABC

The mTOR, a key protein kinase, acts as a nutrient sensor and monitor of the cellular metabolic state regulating protein synthesis and ultimately cell growth, proliferation, and survival. mTOR serves a key role in normal mammalian cell physiology, and is centrally involved in tumor-cell physiology, (for example, facilitating cell- cycle progression from G1-S phase) and consequently inhibition of this target has received considerable attention as an anticancer approach, as reviewed by (Bjornsti 2004; Abraham 2007). mTOR regulates global mRNA translation (Beuvink 2005). Indeed, downstream from mTOR is the serine / threonine kinase p70S6 kinase (S6K). S6K phosphorylates key residues on the ribosomal protein S6, permitting its activation and full function as a protein involved in ribosomal biogenesis. The mTOR kinase also modulates phosphorylation of 4E-binding protein 1 (4E-BP1), releasing its

[redacted]

inhibition of eIF-4E and consequently permitting efficient cap-dependent translation (Bjornsti and Houghton 2004).

Activation of the mTOR pathway is a key adaptive change driving endocrine resistance. Research into the mechanisms of resistance has shown that various signal transduction pathways are activated to escape the effect of endocrine therapy. For example, the PI3 kinase/Akt/mTOR pathway is constitutively activated in aromatase inhibitor resistant and long-term estrogen deprivation BC cells (Tokunaga 2006; Santen 2005; Campbell 2001). Selective inhibitor of mTOR, sirolimus or rapamycin, demonstrated a significant growth inhibition particularly in long-term estrogen deprived BC cells (Yue et al 2007). Rapamycin and its analogues partially inhibit mTOR through allosteric binding to mTORC1 but not mTORC2 (Efeyan and Sabatini 2010). However, prolonged exposure to rapamycin also results in mTORC2 inhibition (Sarbassov et al 2006).

1.2 Introduction to investigational treatment(s) and other study treatment(s)

Everolimus first entered clinical development for one of numerous oncology indications in 2002.

It was approved by the FDA on 30-Mar-2009 under the trade name Afinitor® for the treatment of patients with advanced renal cell carcinoma (RCC) after failure of treatment with sunitinib or sorafenib. The European Commission approved Afinitor® on 03-Aug-2009 for the treatment of patients with advanced RCC, whose disease has progressed on or after treatment with vascular endothelial growth factor (VEGF)-targeted therapy. Everolimus has been approved in >121 countries worldwide for the treatment of patients with advanced RCC.

On 05-May-2011, FDA approved Afinitor® for the treatment of progressive neuroendocrine tumors of pancreatic origin (PNET) in patients with unresectable, locally advanced or metastatic disease. The EC approved Afinitor® on 24-Aug-2011 for the treatment of unresectable or metastatic, well- or moderately-differentiated neuroendocrine tumors of pancreatic origin in adults with progressive disease. Everolimus has been approved in >115 countries worldwide for the treatment of patients with advanced pNET/NET.

On 20-Jul-2012, FDA approved Afinitor® for the treatment of postmenopausal women with advanced hormone receptor-positive (HR+), human epidermal growth factor receptor 2 negative (HER2-) breast cancer in combination with exemestane, after failure of treatment with letrozole or anastrozole. The EC approved Afinitor® on 23-Jul-2012 for the treatment of HR+, HER2- advanced breast cancer, in combination with exemestane, in postmenopausal women without symptomatic visceral disease after recurrence or progression following a non-steroidal aromatase inhibitor. Everolimus has been approved in >115 countries worldwide for the treatment of patients with advanced HR+, HER2- breast cancer.

In January 2013, everolimus, under the trade name Afinitor, was approved for the treatment of advanced RCC in China. In February 2014, everolimus (trade name: Afinitor®) was approved for the treatment of pNET and TSC-SEGA in China. Afinitor is approved in more than 105 countries for each of the oncology indications and more than 95 for each of the TSC indications. Most recently everolimus was approved in the US on 26-Feb-2016 for Afinitor for the treatment of adult patients with progressive, well-differentiated, advanced

[redacted]

nonfunctional neuroendocrine tumors (NET) of gastrointestinal (NETs of GI) or lung origin that. Applications for this newest oncology indication are unresectable, locally advanced or metastatic pending worldwide.

The following is a brief summary of the main characteristics of everolimus. More detailed information can be obtained from the everolimus investigator's brochure.

1.2.1 Overview of everolimus

Mechanism of action

Everolimus is a derivative of rapamycin which acts as a signal transduction inhibitor (Table 1-1, Figure 1-1). Everolimus selectively inhibits mammalian target of rapamycin (mTOR), specifically targeting the mTOR-raptor signal transduction complex. mTOR is a key serine-threonine kinase in the phosphoinositide 3-kinase/protein kinase B (PI3K/AKT) signaling cascade, which is known to be dysregulated in a wide spectrum of human cancers (Boulay and Lane 2007).

Everolimus is being investigated as an anticancer agent based on its potential to act

- directly on the tumor cells by inhibiting tumor cell growth and proliferation;
- indirectly by inhibiting angiogenesis leading to reduced tumor vascularity (via potent inhibition of tumor cell VEGF production and VEGF-induced proliferation of endothelial cells).

Table 1-1 Everolimus - drug substance

Chemical name	(1R,9S,12S,15R,16E,18R,19R,21R,23S,24E,26E,28E,30S,32S,35R)-1,18-dihydroxy-12-{(1R)-2-[(1S,3R,4R)-4-(2-hydroxyethoxy)-3-methoxycyclohexyl]-1-methylethyl}-19,30-dimethoxy-15,17,21,23,29,35-hexamethyl-11,36-dioxa-4-aza-tricyclo[30.3.1.$0^{4,9}$]hexatriaconta-16,24, 26,28-tetraene-2,3,10,14,20-pentaone
International non-proprietary name	Everolimus

Figure 1-1 Chemical structure of everolimus

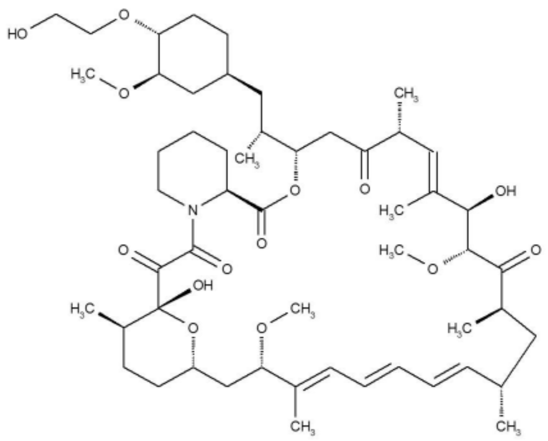

[redacted]

mTOR pathway and cancer

At the cellular and molecular level, everolimus acts as a signal transduction inhibitor. It selectively inhibits mTOR, a key protein kinase which regulates cell growth, proliferation and survival. The mTOR kinase is mainly activated via the PI3K pathway through AKT/PKB and TSC1/2. Mutations in these components or in phosphatase and tensin homolog (PTEN), a negative regulator of PI3K, may result in their dysregulation. Abnormal functioning of various components of the signaling pathways contributes to the pathophysiology of numerous human cancers. Various preclinical models have confirmed the role of this pathway in tumor development (Cohen et al 2005).

The main known functions of mTOR include the following (Bjornsti and Houghton 2004):

- mTOR functions as a sensor of mitogens, growth factors and energy and nutrient levels;
- Facilitating cell-cycle progression from G1-S phase in appropriate growth conditions;
- The PI3K/mTOR pathway itself is frequently dysregulated in many human cancers, and oncogenic transformation may sensitize tumor cells to mTOR inhibitors;
- PI3K mutations have been reported in the primary tumor in 10-20% of human colorectal cancers (Frattini 2005, Velho 2005);
- The loss of PTEN protein, either through gene deletion or functional silencing (promoter hypermethylation), is reported in approximately 60% of primary human colorectal cancers (Goel et al 2004);
- The mTOR pathway is involved in the production of pro-angiogenic factors (i.e., VEGF) and inhibition of endothelial cell growth and proliferation;
- Through inactivating eukaryotic initiation factor 4E binding proteins and activating the 40S ribosomal S6 kinases (i.e., p70S6K1), mTOR regulates protein translation, including the HIF-1 proteins. Inhibition of mTOR is expected to lead to decreased expression of HIF-1.

1.2.1.1 Non-clinical experience

Everolimus inhibits the proliferation of a range of human tumor cell lines in vitro including lines originating from lung, breast, prostate, colon, melanoma and glioblastoma. Inhibitory concentration at 50% (IC50s) ranges from sub/low nM to µM. Everolimus also inhibits the proliferation of human umbilical vein endothelial cells (HUVECS) in vitro, with particular potency against VEGF-induced proliferation suggesting that everolimus may also act as an anti-angiogenic agent. The antiangiogenic activity of everolimus was confirmed in vivo. Everolimus selectively inhibited VEGF-dependent angiogenic response at well tolerated doses. Mice with primary and metastatic tumors treated with everolimus showed a significant reduction in blood vessel density when compared to controls.

The potential of everolimus as an anticancer agent was shown in rodent models. Everolimus is orally bioavailable, residing longer in tumor tissue than in plasma in a subcutaneous mouse xenograft model, and demonstrating high tumor penetration in a rat pancreatic tumor model. The pharmacokinetic profile of everolimus indicates sufficient tumor penetration, above that needed to inhibit the proliferation of endothelial cells and tumor cell lines deemed sensitive to everolimus in vitro.

[redacted]

Everolimus administered orally daily was a potent inhibitor of tumor growth, at well tolerated doses, in 11 different mouse xenograft models (including pancreatic, colon, epidermoid, lung and melanoma) and two syngeneic models (rat pancreatic, mouse orthotopic melanoma). These models included tumor lines considered sensitive and "relatively resistant" in vitro. In general, everolimus was better tolerated in mouse xenograft models than standard cytotoxic agents (i.e., doxorubicin and 5-fluorouracil), while possessing similar anti-tumor activity.

Additionally, activity in a VEGF-impregnated subcutaneous implant model of angiogenesis and reduced vascularity (vessel density) of everolimus-treated tumors (murine melanoma) provided evidence of *in vivo* effects of angiogenesis.

It is not clear which molecular determinants predict responsiveness of tumor cells to everolimus. Molecular analysis has revealed that relative sensitivity to everolimus in vitro correlates with the degree of phosphorylation (activation) of the AKT/PKB protein kinase and the S6 ribosomal protein; in some cases (i.e., glioblastoma) there is also a correlation with Phosphatase and Tensin Homolog (PTEN) status.

In vivo studies investigating the anti-tumor activity of everolimus in experimental animal tumor models showed that everolimus monotherapy typically reduced tumor cell growth rates rather than produced regressions. These effects occurred within the dose range of 2.5 mg to 10 mg/kg, orally once a day.

In preclinical models, the administration of everolimus is associated with reduction of protein phosphorylation in target proteins downstream of mTOR, notably phosphorylated S6 (p-S6) and p-4E-BP1, and occasionally with an increase in phosphorylated AKT, a protein upstream of mTOR signaling pathway.

All significant adverse events observed in toxicology studies with everolimus in mice, rats, monkeys and mini-pigs were consistent with its anticipated pharmacological action as an anti-proliferative and immunosuppressant and at least in part reversible after a 2 or 4-week recovery period with the exception of the changes in male reproductive organs, most notably testes. Further details can be found in the everolimus [Investigator's Brochure].

1.2.1.2 Clinical experience

1.2.1.2.1 Everolimus pharmacokinetics

Everolimus is rapidly absorbed with a median t_{max} of one to two hours. The steady-state area under the concentration time curve AUC0-τ is dose-proportional over the dose range between 5 to 70 mg in the weekly regimen and 5 and 10 mg in the daily regimen. Steady-state was achieved within two weeks with the daily dosing regimen. C_{max} is dose-proportional between 5 and 10 mg for both the weekly and daily regimens. At doses of 20 mg/week and higher, the increase in C_{max} is less than dose proportional (amended [Study C2102 CP report]).

In healthy patients, high fat meals reduced systemic exposure to everolimus 10 mg (as measured by AUC) by 22% and the peak plasma concentration C_{max} by 54%. Light fat meals reduced AUC by 32% and C_{max} by 42%. Food, however, had no apparent effect on the post absorption phase concentration-time profile [Study C2120].

[redacted]

The blood-to-plasma ratio of everolimus, which is concentration-dependent over the range of 5 to 5,000 ng/mL, is 17% to 73%. The amount of everolimus confined to the plasma is approximately 20% at blood concentrations observed in cancer patients given everolimus 10 mg/day [Study 303-044]. Plasma protein binding is approximately 74% both in healthy patients and in patients with moderate hepatic impairment [Study A2303].

The major and nearly exclusive enzyme responsible for the metabolism of everolimus in man was CYP3A4 ([DMPK(US)1998/005]; [DMPK(CH) R99-2448], (Kuhn et al 2001)). Other cytochrome P450 (CYP) isoenzymes either do not metabolize everolimus or do so at very low rates. Everolimus is also a moderate inhibitor of P-glycoprotein-like mediated efflux systems, although the compound has a high intrinsic permeability when P-glycoprotein is inhibited (Crowe 1998, Laplante 2002, [DMPK(CH) 1997/417]). Following oral administration, everolimus is the main circulating component in human blood and is considered to contribute the majority of the overall pharmacologic activity [Study W107].

No specific excretion studies have been undertaken in cancer patients; however, data available from the transplantation setting found the drug to be mainly eliminated through the feces.

Everolimus at a dose of 5 mg/day or 10 mg/day was confirmed PK profile in 24 Chinese patients with advanced solid tumors. Absorption of everolimus after oral administration was rapid to moderate with median tmax of 2 to 3 hours. The Cmin, Cmax, and $AUC_{0-\tau}$ at the dose of 10 mg/day were approximately 2-fold those at 5 mg/day and increased dose-proportionally. After reaching steady-state on Day 8, mean (±SD) values of CL/F were 16.7 (±5.6) and 18.2 (±7.2) L/h at doses of 5 and 10 mg/day, respectively [Study X2101]. The mean (±SD) steady-state Cmax and CL/F values obtained in this study population of Chinese patients were comparable to the values observed in Japanese patients with Non-Hodgkin's Lymphoma after the respective daily doses [Study C1104], and the values observed in Caucasian patients with RCC at the everolimus 10 mg/day once-daily dose [Study C2240], which demonstrated the PK profile of everolimus observed in Chinese patients was consistent with previous experience in oncology setting.

1.2.1.2.2 Efficacy in hormone receptor positive breast cancer

Several randomized trials evaluated everolimus in HR+ breast cancer and showed evidence of efficacy of everolimus in this patient population.

In a multicenter, randomized phase II study, a daily dose of everolimus (10 mg) was evaluated in patients with ABC including patients with HR+ disease previously received endocrine therapy. In this trial, 19 of the 49 patients enrolled were ER-positive/HER2-negative; one complete response, 2 partial responses, 3 stable disease for longer than 6 months, and 6 stable diseases for less than 6 months were reported in this subgroup. Median PFS in this subset of 19 patients was 3.5 months (95% C.I.: 1.9 – 5.5 months, data source: NCI-Canada). An additional partial response was reported in a patient with ER-positive HER2-unknown tumor (Ellard et al 2009).

The combination of everolimus with endocrine therapy has been assessed in different disease settings. In newly diagnosed patients with HR+ early BC, a neoadjuvant randomized 270-patient phase II study compared the combination of everolimus and letrozole to letrozole alone. The overall response rate in the everolimus arm was higher than that with letrozole

[redacted]

alone arm (68% vs. 59% (palpation, p =0.062) and 58% vs. 47% (ultrasound, p =0.021) respectively. Additionally, there was a greater antiproliferative response, with a decrease of the Ki67 proliferation index to <1 in 57% of patients in the everolimus arm and in 30% of patients in the placebo arm (P<0.01). This reduction in Ki67 was observed only two weeks after initiation of trial therapy (Baselga et al 2009).

In a randomized phase III, double-blind, placebo-controlled study (BOLERO-2), everolimus in combination with exemestane was compared with exemestane alone in 724 postmenopausal women with HR+ ABC who had a recurrence or progression on letrozole or anastrozole. The combination of everolimus with exemestane showed significant improvement in efficacy, in terms of PFS, response rate, and clinical benefit rate, relative to exemestane monotherapy (Baselga et al 2011). Everolimus plus exemestane significantly improved PFS versus exemestane monotherapy (HR = 0.45; 95% CI, 0.38-0.54; $P < .0001$) in the overall study population (Yardley 2013). Of 143 patients of Asian origin enrolled in this study, 98 received everolimus plus exemestane and 45 received exemestane plus placebo. At the time of data cut-off, 55 Asian patients (56%) in the combination arm had experienced disease progression compared with 34 patients (76%) in the exemestane plus placebo arm. The combination therapy reduced the risk of disease progression versus exemestane alone by 38% among Asian patients (HR =0.62; 95% CI, 0.41-0.94) corresponding to median PFS prolongation from 4.1 months in the placebo plus exemestane arm to 8.5 months in the everolimus plus exemestane arm (Noguchi et al 2013).

In a 111-patient randomized phase II study in postmenopausal women with ER+ ABC previously pretreated with aromatase inhibitors, the combination of everolimus and tamoxifen showed a significant improvement in progression-free survival (8.6 months vs. 4.5 months, p=0.0021) and overall survival (median not reached vs. 24.4 months, p=0.0137) relative to tamoxifen alone (TAMRAD trial, Bachelot et al 2012).

Both everolimus and exemestane are substrates of CYP3A4 and there is a potential DDI between the two drugs. In BOLERO-2, average exemestane predose concentration (C_{min}) and two-hour post-dose concentration (C_{2h}) were 45% and 64% higher, respectively, when co-administered with everolimus. However, the increase in exemestane level is not likely to have major impact on the efficacy and safety of exemestane. In the everolimus plus exemestane arm, exemestane C_{min} or C_{2h} values were similar for Japanese and non-Japanese patients. In the placebo plus exemestane arm, exemestane C_{min} or C_{2h} values were also similar for Japanese and non-Japanese patients. It was demonstrated that the PK exposure is similar between Japanese and non-Japanese patients.

Safety profile of everolimus

The following adverse events are considered to be the class-effects of the mTOR inhibitors: stomatitis/oral mucositis/ulcers, infections and infestations, rash and similar events, cytopenia, hemorrhages, non-infectious pneumonitis, hyperglycemia/new-onset diabetes mellitus, renal events, and thromboembolism. The more common metabolic side effects reported with mTOR inhibitors result from inhibitory effects on mTOR-regulated lipid and glucose pathways, while infections stem from the immunosuppressive properties of these agents. Virtually all of the side effects associated with mTOR inhibitors can be managed effectively with dose modification and/or supportive intervention.

[redacted]

The safety profile of everolimus observed in the phase III Study Y2301 (BOLERO-2) is consistent with prior experience in the oncology setting; events continue to be predominantly low grade (grade 1 or 2). Compared to the control arm [exemestane + placebo], an increased risk of non-infectious pneumonitis, infection, and stomatitis in the everolimus plus exemestane arm was observed. The most common (in ≥ 10% patients) adverse events (AEs) reported in the patients treated with everolimus plus exemestane were stomatitis, rash, fatigue, diarrhea, decreased appetite, weight decreased, cough, dysgeusia, dyspnea, headache arthralgia, peripheral edema, anemia, nausea, epistaxis, vomiting, pyrexia, pneumonitis, constipation, back pain, pruritus, insomnia, asthenia, AST/ALT/GGT increase, hyperglycemia, dry mouth, alopecia nasopharyngitis and urinary tract infection (Table 1-1). The most common grade 3-4 AEs suspected to be related to treatment with an incidence of ≥ 2% were: stomatitis, fatigue, diarrhea, weight loss, dyspnea, anemia, pneumonitis, asthenia, hyperglycemia and AST/GGT increase.

[redacted]

Table 1-2 BOLERO-2 Study: most commonly reported adverse events (>/= 10% of patients treated with everolimus + exemestane)

	EVE+EXE (n=482), %					Placebo+EXE (n=238), %				
		Grade					Grade			
AE (preferred term)	All	1	2	3	4	All	1	2	3	4
Stomatitis	59	29	22	8	0	12	9	2	1	0
Rash	39	29	9	1	0	7	5	2	0	0
Fatigue	37	18	14	4	<1	27	16	10	1	0
Diarrhea	34	26	6	2	<1	19	14	4	1	0
Nausea	31	21	9	<1	<1	29	21	7	1	0
Decreased appetite	28	19	10	2	0	13	8	4	1	0
Weight decreased	26	10	16	2	0	7	3	5	0	0
Cough	22	21	4	1	0	12	8	3	0	0
Dysgeusia	22	18	4	0	0	6	6	0	0	0
Dyspnea	23	10	6	5	<1	11	8	2	1	<1
Headache	21	17	6	<1	0	15	13	2	0	0
Arthralgia	21	15	5	1	0	17	11	6	<1	0
Peripheral edema	21	14	6	1	0	6	5	1	<1	0
Anemia	21	4	10	7	1	5	2	2	<1	<1
Epistaxis	17	16	2	0	0	1	1	0	0	0
Vomiting	17	11	6	1	<1	13	9	3	1	0
Pyrexia	16	13	3	<1	0	7	6	1	<1	0
Pneumonitis	16	7	6	3	0	0	0	0	0	0
Constipation	15	11	3	1	0	13	8	5	<1	0
Back pain	15	10	5	<1	0	11	6	3	2	0
Pruritus	14	11	3	<1	0	7	5	2	0	0
Insomnia	14	10	4	<1	0	8	6	3	0	0
Asthenia	14	7	6	2	<1	4	3	1	<1	0
AST increased	14	6	5	3	<1	6	2	2	1	0
Hyperglycemia	14	4	5	5	<1	2	1	1	<1	0
ALT increased	12	5	4	3	<1	5	1	2	2	0
Dry mouth	11	10	1	0	0	7	7	<1	0	0
Alopecia	11	9	1	0	0	12	12	0	0	0
Nasopharyngitis	10	9	1	0	0	9	7	2	0	0
Pain in extremity	10	6	3	<1	0	12	5	5	2	0
Urinary tract infection	10	3	7	<1	0	2	<1	2	0	0
GGT increase	10	2	2	5	2	9	1	1	5	2

In the analysis of Asian subset (143), commonly reported adverse events in the combination arm were consistent with the overall population and included stomatitis (80%), rash (49%), and dysgeusia (31%). Adverse events that occurred more frequently in the Asian subset versus Caucasians included stomatitis (80% vs 54%) and rash (49% vs 37%), whereas dyspnea (8% vs 24%) and asthenia (1% vs 17%) occurred less frequently in the Asian subset versus Caucasians (Noguchi et al 2013).

[redacted]

Table 1-3 Any-grade adverse events with >/+ 10% incidence in Asian ABC patients treated with everolimus + exemestane

	**EVE+EXE** (n=482)		**Placebo +EXE** (n=238)	
**Adverse Event, n (%)**	**Non-Asian (n = 384)**	**Asian (n = 98)**	**Non-Asian (n = 193)**	**Asian (n = 45)**
Stomatitis	207 (54)	78 (80)	21 (11)	7 (16)
Rash	140 (36)	49 (50)	12 (6)	4 (9)
Dysgeusia	76 (20)	30 (31)	11 (6)	3 (7)
Fatigue	154 (40)	24 (24)	57 (30)	8 (18)
Headache	85 (22)	24 (24)	30 (16)	5 (11)
Diarrhea	142 (37)	23 (23)	38 (20)	6 (13)
Weight decreased	110 (29)	23 (23)	14 (7)	3 (7)
Pneumonitis	54 (14)	23 (23)	0	0
Nausea	125 (33)	22 (22)	57 (30)	11 (24)
Nasopharyngitis	27 (7)	22 (22)	12 (6)	9 (20)
Nail disorder	18 (5)	22 (22)	1 (< 1)	0
Constipation	50 (13)	20 (20)	27 (14)	5 (11)
Cough	103 (27)	20 (20)	25 (13)	3 (7)
Decreased appetite	129 (34)	19 (19)	27 (14)	4 (9)
AST increased	49 (13)	18 (18)	12 (6)	1 (2)
ALT increased	42 (11)	17 (17)	10 (5)	1 (2)
Pyrexia	61 (16)	16 (16)	12 (6)	4 (9)
Arthralgia	83 (22)	16 (16)	36 (19)	4 (9)
Epistaxis	68 (18)	15 (15)	2 (1)	1 (2)
Thrombocytopenia	46 (12)	15 (15)	1 (< 1)	0
Anemia	86 (22)	14 (14)	11 (6)	1 (2)
LDH increased	15 (4)	14 (14)	3 (2)	1 (2)
Vomiting	71 (18)	13 (13)	28 (15)	4 (9)
Insomnia	54 (14)	13 (13)	18 (9)	2 (4)
Interstitial lung disease	5 (1)	13 (13)	0	0
Pruritus	53 (14)	12 (12)	8 (4)	3 (7)
Back pain	62 (16)	11 (11)	23 (12)	2 (4)

No new safety concerns have emerged compared to previous experience with everolimus monotherapy or combination therapy.

1.2.2 Overview of exemestane

Exemestane is an irreversible steroidal aromatase inactivator that has demonstrated efficacy in the treatment of postmenopausal patients with ABC. It is indicated for adjuvant treatment of postmenopausal women with Estrogen receptor positive (ER+) early BC who have received two to three years of tamoxifen and are switched to exemestane for completion of a total of five consecutive years of adjuvant endocrine therapy. It is also indicated for the treatment of advanced breast cancer (ABC) in postmenopausal women whose disease has progressed following tamoxifen therapy (in the USA and China) or following anti-oestrogen therapy (in Europe).

[redacted]

Exemestane is initially recognized by the aromatase enzyme as a false substrate and then transformed through an NADPH-dependent mechanism to an intermediate that binds irreversibly to the enzyme causing inactivation. Exemestane significantly lowers circulating estrogen concentrations (estradiol, estrone and estrone sulfate) but has no detectable effect on adrenal biosynthesis of corticosteroids or aldosterone ([Aromasin prescribing information PI-Pfizer-Pharmacia 2005]).

The recommended daily dose of exemestane is 25 mg via oral administration. Exemestane is rapidly absorbed from the gastrointestinal tract. Its bioavailability is limited by first-pass metabolism, but is increased when taken with food. Exemestane is widely distributed, and is extensively bound to plasma proteins. It appears to be more rapidly absorbed in women with breast cancer (tmax of 1.2 hours) than in healthy women (tmax of 2.9 hours). The terminal halflife for exemestane is 18-24 hours. The time needed to reach maximal E2 suppression is 7 days (Demers 1993, Plourde 1995, Buzdar 2003). Exemestane is metabolized by CYP3A4 and aldoketoreductases. It does not inhibit any of the major CYP isoenzymes, including CYP 1A2, 2C9, 2D6, 2E1 and 3A4. Although no formal drug-drug interaction studies have been conducted, significant effects on exemestane clearance by CYP isoenzyme inhibitors appear unlikely ([Aromasin prescribing information-Pfizer-Pharmacia 2011], Hutson 2005, Buzdar 2003).

The most frequently reported adverse effects for exemestane are gastrointestinal disturbances, hot flushes, arthralgia, myalgia, sweating, fatigue, and dizziness. Other reported effects include headache, insomnia, somnolence, depression, skin rashes, alopecia, asthenia, and peripheral and leg edema. Thrombocytopenia and leucopenia have been reported occasionally. Reductions in bone mineral density can occur with long-term use of exemestane. A total of 1058 patients were treated with exemestane 25 mg once daily in the clinical trials program. Exemestane was generally well tolerated, and adverse events were usually mild to moderate. Adverse events occurring in greater than 10% of patients include hot flushes (14%), nausea (11.9%), insomnia, headache, increased sweating, joint and musculoskeletal pain, and fatigue (USPI; Aromasin summary of product characteristics August 2008 (UK as reference member state for EU mutual recognition procedure)). Androgenic effects were reported in a limited number of patients (4.3%) (Buzdar et al 2003).

Refer to the package insert of the local supply of exemestane for more details.

2 Rationale

2.1 Study rationale and purpose

This study aims at evaluating the efficacy and safety of everolimus plus exemestane in Chinese postmenopausal women with ER+ HER2- ABC after recurrence or progression on letrozole or anastrozole.

The rationale of this study is based on the following:

- Everolimus activity in breast cancer in combination with endocrine therapy (Baselga 2009; Baselga 2011; Bachelot 2012)

[redacted]

- Positive efficacy and manageable safety profile of everolimus in combination with exemestane in large Ph III randomized study (Baselga 2011) and in Asian patient population of BOLERO-2 (Noguchi et al 2013)

In a Phase II study, 49 patients who received no or one prior chemotherapy regimen for metastatic breast cancer received everolimus either 10 mg daily or 70 mg weekly (Ellard et al 2009). Majority of patients in the study had had prior chemotherapy for advanced disease and was ER positive (29/49) and HER2 negative (40/49). 19 of the 49 patients with ER+/HER2- enrolled were treated with everolimus 10 mg daily; one complete response, 2 partial responses, 3 stable disease for longer than 6 months, and 6 stable diseases for less than 6 months were reported in this subgroup. Median PFS in this subset of 19 patients was 3.5 months (95% C.I.: 1.9 – 5.5 months, data source: NCI-Canada). An additional partial response was reported in a patient with ER-positive HER2-unknown tumor. The study demonstrated that daily therapy with 10 mg is worthy of further study in this patient population (Ellard et al 2009).

A GINECO study in patients with HR+, human epidermal growth factor receptor 2- metastatic breast cancer with prior exposure to aromatase inhibitors showed that 6-month CBR was 61% (95% CI, 47 to 74) with tamoxifen 20 mg/day plus everolimus 10 mg/day and 42% (95% CI, 29 to 56) with tamoxifen alone (Bachelot et al 2012). Time to progression increased from 4.5 months with tamoxifen alone to 8.6 months with tamoxifen plus everolimus, corresponding to a 46% reduction in risk of progression with the combination (hazard ratio [HR], 0.54; 95% CI, 0.36 to 0.81). Risk of death was reduced by 55% with tamoxifen plus everolimus versus tamoxifen alone (HR, 0.45; 95% CI, 0.24 to 0.81) (Bachelot et al 2012).

A neoadjuvant phase II study in newly diagnosed patients with ER+ early BC showed the overall response rate in the combination of everolimus and letrozole was significantly higher than letrozole alone (68% vs. 59% by palpation, p = 0.062 and 58% vs. 47% by ultrasound, p= 0.021) (Baselga et al 2009).

In BOLERO-2, the combination therapy of everolimus and exemestane has shown superiority in terms of PFS compared to exemestane alone (Baselga 2011; Yardley 2013). In the most current report of 510 PFS events at a median follow-up of 18 months, the addition of everolimus to exemestane significantly prolonged median PFS versus exemestane monotherapy as per local assessment (7.8 vs 3.2 mo, respectively; HR=0.45 [95% CI, 0.38 - 0.54]; log-rank p<0.0001) (Yardley 2013). Furthermore, these data were consistent with results based on central assessment (11.0 mo for everolimus plus exemestane vs 4.1 mo for exemestane alone; HR=0.38 [95% CI, 0.31-0.48]; log-rank p<0.0001). Fewer deaths were reported with everolimus plus exemestane (25.4%) vs exemestane (32.2%). In addition, PFS benefits with everolimus plus exemestane versus exemestane were consistent in all prospectively defined subgroups, including subsets of patients with visceral metastases (n=406), 3 or more sites of metastasis (n=271), and Eastern Cooperative Oncology Group (ECOG) performance status 1 or 2 (n=274) (Yardley 2013). The subgroup analysis in Asian patients in BOLERO-2 showed median PFS of 8.48 months in everolimus plus exemestane arm compared to 4.14 months in placebo plus exemestane arm (Noguchi et al 2013). The combination therapy reduced the risk of disease progression versus exemestane alone by 38% among Asian patients (HR = 0.62; 95% CI, 0.41-0.94).

[redacted]

On 20-Jul-2012, FDA approved Afinitor® for the treatment of postmenopausal women with advanced hormone receptor-positive, HER2-negative breast cancer in combination with exemestane, after failure of treatment with letrozole or anastrozole. The EC approved Afinitor® on 23-Jul-2012 for the treatment of hormone receptor-positive, HER2/neu negative advanced breast cancer, in combination with exemestane, in postmenopausal women without symptomatic visceral disease after recurrence or progression following a non-steroidal aromatase inhibitor.

Based on the results from the studies aforementioned, the NCCN guideline for breast cancer (Version 3.0, 2012) has recommended the use of the combination of everolimus and exemestane for postmenopausal women with HR+ HER2- ABC after failure of NSAI. In light of the need for this new treatment option for Chinese patients, it is imperative and reasonable to conduct a study in Chinese patients to assess the efficacy and safety of the combination of everolimus and exemestane in this target population. Given the available strong evidence provided by the BOLERO-2 study, the benefit of everolimus plus exemestane in the target population and also in the Asian subgroup are obvious and have been acknowledged. Therefore, it is not necessary to do a large sample size randomized study in Chinese patients. This phase II, randomized, control study is to meet China registration purpose and to confirm the efficacy and safety of everolimus plus exemestane in Chinese patients.

2.2 Rationale for the study design

This is a phase II, double-blind, placebo controlled, multicenter study conducted in China. This study design is well-established to assess the efficacy and safety of everolimus/exemestane combination.

2.3 Rationale for dose and regimen selection

The selection of the 10 mg continuous daily dose for everolimus is based on a pharmacodynamic model (Tanaka et al 2008), which was supported by results of a clinical pharmacodynamic study in patients with solid tumors (Tabernero et al 2008). The 10 mg daily dose of everolimus was favored over a 5 mg daily dose in Study C2108, a Phase I study combining everolimus with letrozole in postmenopausal patients with advanced breast cancer (Awada et al 2008). Everolimus 10 mg/day was effectively used as monotherapy in patients with recurrent/metastatic breast cancer (Ellard et al 2009). The same dose of everolimus showed positive efficacy outcomes in combination with letrozole as neoadjuvant therapy in patients with ER+ early breast cancer and in combination with tamoxifen in patient with ABC (Baselga 2009, Bachelot 2012). This was further corroborated by results from BOLERO-2 study, where a daily regimen of 10 mg everolimus in combination with exemestane 25 mg daily was more efficacious than exemestane alone (Piccart et al 2012).

The recommended daily dose of exemestane is 25 mg via oral administration.

2.4 Rationale for choice of combination drugs

Results in BOLERO-2 study demonstrated PFS benefit of the combination of everolimus and exemestane compared to exemestane alone in postmenopausal women with HR+ HER2-, ABC who has a recurrence or progression on NSAI with acceptable safety profile (Yardley et

[redacted]

al 2013). The approved doses in the treatment of postmenopausal women with HR+ HER2- and ABC are 10 mg daily of everolimus plus 25 mg daily of exemestane via oral administration.

2.5 Risks and benefits

The risk to subjects in this trial may be minimized by compliance with the eligibility criteria and study procedures, close clinical monitoring, and dose modification, early termination procedure. There may be unforeseen risks with study treatment which could be serious.

[redacted]

3 Objectives and endpoints

Table 3-1 Objectives and related endpoints

Objective	Endpoint	Analysis
Primary		Refer to Section 10.4.
To assess the combination treatment of everolimus and exemestane to exemestane and placebo with respect to progression-free survival	PFS, defined as the time from the date of randomization to the date of the first documented progression (as assessed by local assessment per RECIST 1.1) or death due to any cause	
Secondary		Refer to Section 10.5.
To assess the two treatment arms with respect to PFS as determined by a Blinded Independent Review Committee (BIRC).	PFS, defined as the time from the date of randomization to the date of the first documented progression (as assessed by BIRC assessment per RECIST 1.1) or death due to any cause	
To assess the two treatment arms with respect to overall survival	OS, defined as time from date of randomization to date of death due to any cause	
To assess the two treatment arms with respect to following based on local investigator assessment and BIRC<br>• Overall response rate (ORR)<br>• Clinical benefit rate (CBR)<br>• To summarize time to response and duration of response in the two treatment arms	The following endpoints will be evaluated by Investigator and BIRC assessment per RECIST 1.1:<br>• ORR, defined as the proportion of patients with a best overall response defined as complete response (CR) or partial response (PR); (CR+PR)<br>• CBR, defined as the proportion of patients with best overall response of complete response (CR), partial response (PR) or stable disease (SD) with duration of 24 weeks or longer.<br>• Time to response, defined as the time between date of randomization until first documented response (CR or PR)<br>• DOR, defined as the time from date of first documented CR or PR to date of first documented disease progression or death due to any cause	
Time to deterioration of ECOG Performance Status	ECOG PS categories are defined in Section 7.2.2.	
To characterize the safety and tolerability of everolimus and exemestane versus placebo and exemestane in all patients	Adverse events (AEs), ECGs and laboratory abnormalities	

[redacted]

Objective	Endpoint	Analysis
Characterize the pharmacokinetics of everolimus (Cmin, C2h) when administered in combination with exemestane		
Compare the two treatment arms with respect to pre-dose concentration (Cmin) and concentration at 2 hours post dose (C2h) of exemestane and to compare the two treatment arms with respect to estradiol (E2) changes from baseline.		

4 Study design

4.1 Description of study design

This is a multicenter, double-blind, randomized, placebo-controlled, phase II study evaluating treatment with everolimus (10 mg daily) versus placebo in combination with exemestane (25 mg daily) in Chinese postmenopausal women with locally advanced, recurrent or metastatic ER positive HER2-negative breast cancer refractory to non-steroidal aromatase inhibitors.

Approximately 160 patients will be randomized in 1:1 ratio to receive either everolimus or matching placebo in a blinded manner in addition to open label exemestane (25 mg daily tablets).

Screening phase

Written informed consent must be obtained before any study specific medical procedures are performed. The investigator or his/her authorized designee will assign a unique number (subject number) to patients being considered for the study. Once assigned, the subject numbers for patients will not be reused.

The study will use Interactive Response Technology (IRT) for patient screening and management of everolimus drug supply. Exemestane will be supplied locally by Novartis in accordance with the country regulations.

After the patient signs the informed consent and prior to the first dose of study treatment, patients will be screened in IRT. All inclusion/exclusion criteria must be verified. Patients who do not meet the criteria will not be given the study treatment.

All screening assessments to confirm eligibility must be performed within maximum 21 days prior to the first dose of study treatment (Table 7-1 and Section 7.1).

Screening for hepatitis B

During the screening phase, all patients should be tested for hepatitis B viral load and serologic markers of HBV-DNA, HBsAg, HBsAb, and HBcAb.

[redacted]

Patients with positive hepatitis B results should start prophylactic antiviral treatment of 1 to 2 weeks prior to the first doses of the study treatment, and should continue for at least 4 weeks after last dose of study treatment.

The management guidelines, in Section 6.3.2.3, are provided according to the results of the baseline assessment of viral load and serological markers for hepatitis B.

Screening for hepatitis C

Patients with any of the following risk factors for hepatitis C should be tested using quantitative RNA-PCR

- known or suspected past hepatitis C infection (including patients with past interferon ‘curative’ treatment),
- blood transfusions prior to 1990,
- current or prior IV drug users,
- current or prior dialysis,
- household contact of hepatitis C infected patient(s),
- current or prior high-risk sexual activity,
- body piercing or tattoos,

At the discretion of the investigator, additional patients may also be tested for hepatitis C. The management guidelines, in Section 6.3.2.3, are provided according to the results of the baseline assessment of hepatitis C viral load.

Randomized treatment phase

All eligible patients will be randomized within 7 days before Cycle 1 Day 1 to one of the two treatment arms: everolimus plus exemestane arm or placebo plus exemestane arm. Randomization will be stratified according to the presence of visceral disease and sensitivity to prior hormonal therapy status. Visceral refers to lung, liver, brain, pleural and peritoneal involvement. Sensitivity to prior hormonal therapy is defined as either (1) documented clinical benefit (complete response, partial response, stable disease ≥ 24 weeks) to at least one prior hormonal therapy in the advanced setting or (2) at least 24 months of adjuvant hormonal therapy prior to recurrence.

Randomized patients will start the study treatment at Cycle 1 Day 1. Patients will receive everolimus/placebo (10 mg daily oral tablets) in addition to exemestane (25 mg daily oral tablets) continuously. Dose adjustment (reduction, interruption) according to safety findings will be allowed (Section 6.3).

Patients will receive treatment until disease progression (assessed by RECIST 1.1), unacceptable toxicity, death or discontinuation from treatment for any other reason. Tumor assessments will be performed every 8 weeks after randomization until radiological progression per local assessment. Tumor assessments are to be performed every 12 weeks after data cut-off for primary analysis and as clinically indicated, until disease progression.

Imaging data used for tumor assessments during treatment phase and follow-up phase will be centrally collected, checked for quality and reviewed by a central vendor designated by

[redacted]

Novartis. The central review of the scans will be carried out in a blinded fashion. The decision regarding patient management will remain with the local investigator.

Post-treatment follow-up phase

After end of treatment visit, all patients will be followed up for safety up to 30 days after last dose of study treatment (exemestane and/or everolimus/placebo).

All patients who discontinue from study treatment due to disease progression must have their progression clearly documented according to the criteria specified in RECIST 1.1.

If a patient permanently discontinues study treatment for reasons other than disease progression, death, lost to follow-up, or withdrawal of consent to efficacy follow-up then tumor assessments should continue to be performed per assessment schedule until disease progression, death, lost to follow-up or withdrawal of consent for efficacy follow-up.

Antineoplastic therapy regimen received between EOT and progression will also be collected as well as the first regimen received after progression.

Survival follow-up phase

All patients will be followed for survival status at least every 3 months after randomized treatment phase or post-treatment follow-up phase unless they discontinued due to death, consent withdrawal or lost to follow-up. Survival information can be obtained via phone and information will be documented in the source documents and eCRF. Additional survival assessments may be performed more frequently if a survival update is required for reporting the results or to meet safety or regulatory needs.

[redacted]

Figure 4-1 Study design

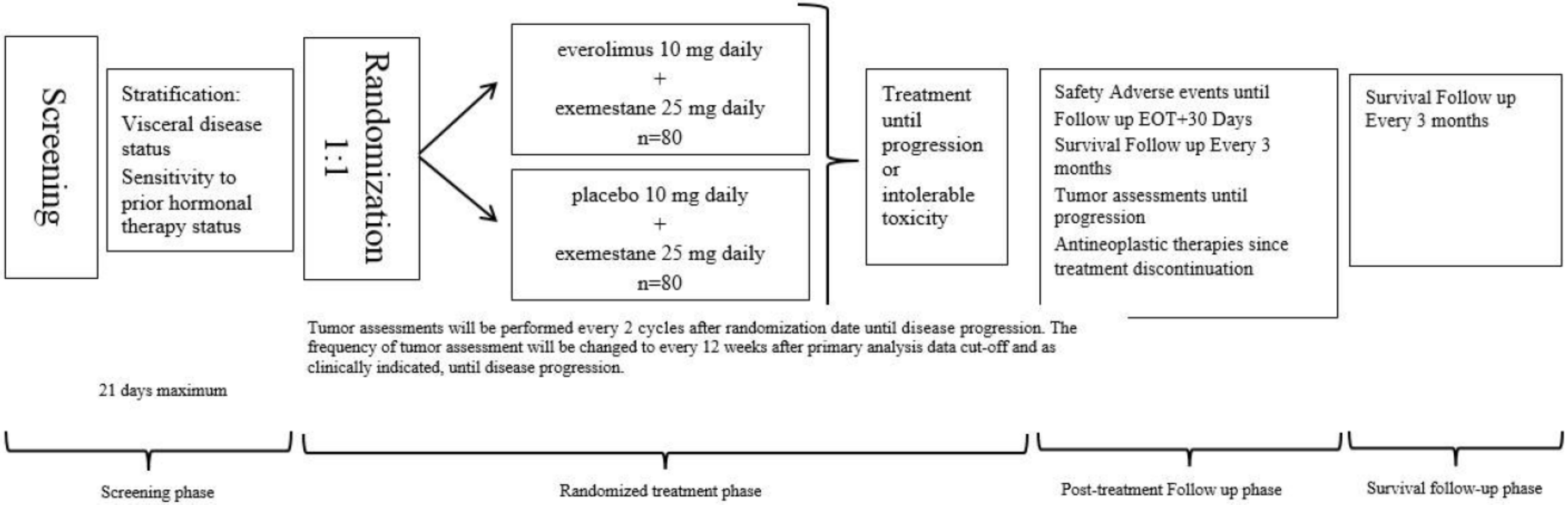

[redacted]

4.2 Timing of interim analyses and design adaptations

Not applicable.

4.3 Definition of end of study

The primary analysis will occur when approximately 110 PFS events have been documented based on local assessment (refer to Section 10.4). At this time, the primary clinical study report (CSR) will be produced.

After the primary analysis of PFS, the study will remain open provided the PFS demonstrates treatment benefit. Patients still being followed on the study after the primary analysis time point will continue as per the schedule of assessments.

The end of study is defined as the earliest occurrence of one of the following:

- All patients have died or discontinued from the study
- Another clinical study or patient support programs becomes available that can continue to provide Afinitor in this patient population and all patients ongoing are eligible to be transferred to them
- Afinitor has been approved for this indication in combination with Exemestane

The final analysis of study data will be conducted at the end of the study. All available data from all patients up to this cutoff date will be analyzed.

4.4 Early study termination

The study can be terminated at any time for any reason by Novartis. Should this be necessary, the patient will be contacted by the investigator or his/her designee. The patient should be seen as soon as possible for an end of treatment visit / or end of post treatment follow up visit and the same assessments should be performed as described in Section 7 for a discontinued or withdrawn patient. The investigator may be informed of additional procedures to be followed in order to ensure that adequate consideration is given to the protection of the patient's interests. The investigator will be responsible for informing IRBs and/or ECs of the early termination of the trial.

5 Population

5.1 Patient population

Chinese Postmenopausal women with ER+ HER2- locally advanced, recurrent, or metastatic breast cancer after recurrence or progression on prior letrozole or anastrozole.

Patients enrolled in this study are not permitted to participate in additional parallel investigational drug or device studies. Patients who have discontinued this study won't be re-enrolled.

The investigator or designee must ensure that only patients who meet all the following inclusion and none of the exclusion criteria are offered treatment in the study.

[redacted]

5.2 Inclusion criteria

Patients eligible for inclusion in this study have to meet **all** of the following criteria:

1. Women (18 years or older) with locally advanced, recurrent, or metastatic breast cancer. Locally advanced breast cancer must not be amenable to curative treatment by surgery or radiotherapy.
2. Histological or cytological confirmation of estrogen-receptor positive (ER+) breast cancer
3. Postmenopausal women. Postmenopausal status is defined either by:

- Prior bilateral oophorectomy
- Or age ≥60
- Or age < 60 and amenorrhea for 12 or more months (in the absence of chemotherapy, tamoxifen, toremifen, or ovarian suppression), and FSH and estradiol in the postmenopausal range (serum FSH > 40 mIU/mL and estradiol <20 pg/mL or according to the postmenopausal range defined by local laboratory).

For women with therapy-induced amenorrhea, oophorectomy or serial measurements of

FSH and/or estradiol are needed to ensure postmenopausal status.

Note: Ovarian radiation or treatment with a luteinizing hormone-releasing hormone (LH- RH) agonist (goserelin acetate or leuprolide acetate) is not permitted for induction of ovarian suppression.

4. Recurrence or progression on prior NSAI is defined as:
   - Recurrence while on, or within one year (12 months) of end of adjuvant treatment with letrozole or anastrozole

   or

   - Progression while on or within one month (30 days) of the end of prior treatment with letrozole or anastrozole

Notes:

- Letrozole or anastrozole do not have to be the last treatment prior to the enrollment in the study
- Patients must have improved to grade 1 or better from any adverse events (with exception of alopecia) related to previous therapy prior to enrollment

5. Radiological or objective evidence of recurrence or progression on or after the last systemic therapy prior to enrollment
6. Patients must have recovered to grade 1 from any adverse events (except alopecia) related to prior therapy prior to randomization.
7. Patient must have as per RECIST 1.1
   - measurable disease

   or

   - non-measurable lytic or mixed (lytic + blastic) bone lesions in the absence of measurable disease.

Note: Measurable lesions include lytic or mixed (lytic + blastic) bone lesions, with an identifiable soft tissue component that meets the measurability criteria per RECIST 1.1.

[redacted]

Patients with only non-measurable lesions (e.g. pleural effusion, ascites) and no lytic or a mix of lytic and blastic bone lesions are not eligible.

8. Patient is able to swallow and retain oral medication
9. Patient must meet the following laboratory values at the screening visit:
   - Absolute Neutrophil Count (ANC) ≥1.5 x 10^9/L
   - Platelets ≥100 x 10^9/L
   - Hemoglobin (Hgb) ≥9 g/dL
   - INR≤2
   - Serum creatinine ≤1.5 x ULN
   - Total bilirubin ≤1.5 x ULN (≤ 3 × ULN for patients known to have Gilbert Syndrome)
   - Aspartate transaminase (AST) ≤ 2.5 x ULN, except for patients with liver metastasis, who may only be included if AST ≤5.0 x
   - Alanine transaminase (ALT) ≤ 2.5 x ULN, except for patients with liver metastasis, who may only be included if ALT ≤5.0 x ULN
   - Fasting serum cholesterol ≤ 300 mg/dl or 7.75 mmol/L and fasting triglycerides ≤ 2.5 x ULN. In case one or both of these thresholds are exceeded, the patient can only be included after initiation of statin therapy and when the above mentioned values have been achieved
10. Patient has a WHO performance status ≤ 2
11. Written informed consent must be obtained prior to any screening procedures.

5.3 Exclusion criteria

Patients eligible for this study must not meet **any** of the following criteria:

1. HER2-overexpressing patients by local laboratory testing (IHC 3+ staining or in situ hybridization positive), based on the most recent test. Note: Patients with IHC 2+ must have a negative in situ hybridization test.
2. Patients who received more than one chemotherapy line for ABC

Note: A chemotherapy line in advanced disease is an anticancer regimen that contains at least one chemotherapy agent and is given for 21 days or longer. If a cytotoxic chemotherapy regimen was discontinued for a reason other than disease progression and lasted less than 21 days, then this regimen does not count as a "prior line of chemotherapy". Chemotherapy regimens composed of more than one drug are considered as one line of therapy.

3. Patient with symptomatic visceral disease and is candidate to chemotherapy
4. Patients with only non-measurable lesions other than lytic or mixed (lytic and blastic) bone metastasis (e.g. pleural effusion, ascites etc.)
5. Previous treatment with exemestane, mTOR inhibitors, PI3K inhibitors, AKT inhibitors.
6. Known hypersensitivity to mTOR inhibitors, e.g. sirolimus (rapamycin)
7. Patients who have a history of another primary malignancy, with the exceptions of non-melanoma skin cancer, and carcinoma in situ of the cervix, uteri, or breast who have been disease free for ≥3 years

[redacted]

8. Radiotherapy within four weeks prior to enrollment in the study except in case of localized palliative radiotherapy (for analgesic purpose) or for lytic lesions at risk of fracture which was completed at least two weeks prior to registration in the current study. Patients must have recovered from radiotherapy toxicities.
9. Currently receiving any hormone replacement therapy, unless discontinued prior to registration in the current study.
10. Patients with central nervous system (CNS) involvement unless they meet ALL of the following criteria:
    - At least 4 weeks from prior therapy completion (including radiation and/or surgery) to starting the study treatment.
    - Clinically stable CNS lesions at the time of screening which are untreated or without evidence of progression for at least 4 weeks after treatment, as determined by clinical examination and brain imaging (MRI or CT). Patients must not be treated with steroids for brain metastases during the screening period
11. Patients receiving concomitant immunosuppressive agents or chronic corticosteroids use at the time of study entry except topical applications, inhaled sprays, eye drops or local injections.
12. Bilateral diffuse lymphangitic carcinomatosis
13. Severely impaired lung function as defined as spirometry and DLCO that is 50% or less of the normal predicted value and/or O_2 saturation that is 88% or less at rest on room air
14. Patients who have serious liver disease such as cirrhosis, decompensated liver disease, and acute or currently active hepatitis (i.e. quantifiable HBV-DNA and/or positive HbsAg, quantifiable HCV-RNA)
15. Patients with a known history of HIV seropositivity. Screening for HIV infection at baseline is not required.
16. Active, bleeding diathesis, or on oral anti-vitamin K medication (except low dose warfarin, LMWH and acetylsalicylic acid or equivalent, as long as the INR is ≤2).
17. Impairment of gastrointestinal (GI) function or GI disease that may significantly alter the absorption of study treatment.
18. Uncontrolled diabetes mellitus as defined by HbA1c >7% despite adequate therapy. Patients with a known history of impaired fasting glucose or diabetes mellitus (DM) may be included, however, blood glucose and antidiabetic treatment must be monitored closely throughout the trial and adjusted as necessary;
19. Active ulceration of the upper gastrointestinal tract.
20. Any severe and / or uncontrolled medical conditions such as
    a. unstable angina pectoris, symptomatic congestive heart failure, myocardial infarction ≤6 months prior to randomization, serious uncontrolled cardiac arrhythmia,
    b. active or uncontrolled severe infection,
21. Patients being treated with drugs recognized as being strong inhibitors or inducers of the isoenzyme CYP3A4 (Rifabutin, Rifampicin, Clarithromycin, Ketoconazole, Itraconazole, Voriconazole, Ritonavir, Telithromycin) continuously for at least 7 days during any time period in the last 2 weeks prior to registration in the current study

[redacted]

22. Patients who have received live attenuated vaccines within 1 week of start of study drug and during the study. Patient should also avoid close contact with others who have received live attenuated vaccines. Examples of live attenuated vaccines include intranasal influenza, measles, mumps, rubella, oral polio, BCG, yellow fever, varicella and TY21a typhoid vaccines;
23. History of noncompliance to medical regimens
24. Patients unwilling to or unable to comply with the protocol
25. Patients who are currently part of or have participated in any clinical investigation with an investigational drug within 1 month prior to dosing

6 Treatment

6.1 Study treatment

The investigational drug used in the course of this trial is everolimus. In addition, all patients will receive open label exemestane. For this study, the term "Study treatment" is defined as everolimus + exemestane or placebo + exemestane. Study drug is defined as everolimus or matching placebo.

In both treatment arms, the study drug will be administered by continuous oral daily dosing of 2 × 5 mg tablets each morning. For the list of excipients, please refer to current [Investigator's Brochure].

6.1.1 Dosing regimen

All patients will take everolimus/placebo as a daily dose of 10 mg (2 × 5 mg tablets at same time) orally continuously per day and exemestane 25 mg (one tablet) orally per day. Everolimus tablets should be swallowed whole with a glass of water once daily. Tablets should not be chewed or crushed. Exemestane will be self-administered as described in Table 6-1 and taken together with everolimus. Both drugs will be taken after a meal at the same time each day in the morning.

Table 6-1 Dose and treatment schedule

Study treatments	Pharmaceutical form and route of administration	Dose	Frequency and/or Regimen
everolimus/placebo	Tablets for oral use	10 mg (2 × 5 mg)	Daily
exemestane	Tablets for oral use	25mg	Daily

6.1.2 Ancillary treatments

Not applicable.

6.1.3 Rescue medication

Not applicable.

6.1.4 Guidelines for continuation of treatment

For guidelines for continuation of treatment, refer to Section 6.3 Dosing modifications.

[redacted]

Patients who permanently discontinue one of the study treatments for any reason other than disease progression may continue the other study treatment as part of the trial therapy at the investigators discretion until disease progression, unacceptable toxicity, death or discontinuation from study treatment due to any other reason and should follow the protocol safety and efficacy assessments as scheduled. After discontinuing study treatment, further treatment is left to the physician's discretion.

6.1.5 Treatment duration

Patients may be discontinued from treatment earlier due to:

- Disease progression according to RECIST 1.1 as determined by investigator.
- Unacceptable toxicity
- Discontinued treatment at the discretion of the investigator or the patient
- Death
- Patient is lost to follow-up
- Withdraw the consent
- Protocol deviation
- Study termination by sponsor
- Technical problems

6.2 Dose escalation guidelines

Not applicable.

6.3 Dose modifications

6.3.1 Dose modification and dose delay

Everolimus

For patients who do not tolerate the protocol-specified dosing schedule, dose adjustments are permitted in order to allow the patient to continue the study treatment. The following guidelines may be applied:

If dose reduction is required, the suggested dose is approximately 50% lower than the daily dose previously administered (Table 6-2). If a patient has already decreased 2 dose levels, no further dose reduction is permitted.

Patients requiring an additional dose reduction will be required to discontinue study treatment. Patients who interrupt therapy for more than 4 weeks must be discontinued from the study.

These changes must be recorded on the Dosage Administration Record CRF.

Table 6-2 Recommendation of everolimus dose reductions

Dose level	Dose and schedule
0 = starting dose	10 mg daily
-1 dose level	5mg daily
-2 dose level	5mg every other day

[redacted]

All dose modifications should be based on the worst preceding toxicity.

Each patient is only allowed 2 dose reductions. In addition, a patient must discontinue treatment with everolimus/placebo if, after treatment is resumed at a lower dose, the toxicity recurs with the same or worse severity. Patients who interrupt therapy for more than 4 weeks must be permanently discontinued from the study treatment.

Guidelines for dose modification and dose interruption of everolimus/placebo are described in Table 6-3.

The investigators are also advised to consult the latest version of the [Investigator's Brochure]. Objectives and related endpoints are described in Table 3-1 below.

Table 6-3 Criteria for interruption and re-initiation of everolimus/placebo treatment

**Recommended dose modifications for everolimus/placebo**	
Worst toxicity<br>CTCAE Grade (value)	During a cycle of therapy
**Hematologic**	
**Neutropenia (ANC)**	
Grade 1 (ANC < LLN - 1500/mm^3)	Maintain dose level
Grade 2 (ANC < 1500 - 1000/mm^3)	Maintain dose level
Grade 3 (ANC < 1000 - 500/mm^3)	Omit dose until resolved to ≤ Grade 2, then maintain dose level
Grade 4 (ANC < 500/mm^3)	Omit dose until resolved to ≤ Grade 2, then ↓ 1 dose level
**Thrombocytopenia**	
Grade 1 (PLT < LLN - 75,000/mm^3)	Maintain dose level
Grade 2 (PLT < 75,000 - 50,000/mm^3)	Temporary dose interruption until recovery to Grade ≤1.<br>Re-initiate everolimus at the same dose.
Grade 3 (PLT < 50,000 - 25,000/mm^3)	Temporary dose interruption until recovery to Grade ≤1.<br>Re-initiate treatment at a lower dose.
Grade 4 (PLT < 25,000/mm^3)	Temporary dose interruption until recovery to Grade ≤1.<br>Re-initiate treatment at a lower dose.
Febrile neutropenia	
Grade 3<br>(ANC<1.0x10^9/L with single temperature >38.3°C (101°F)<br>or sustained temperature ≥38°C (100.4°F) for >1h)	Temporary dose interruption until recovery of (ANC≥1.25x10^9/l) and no fever.<br>Re-initiate everolimus at a lower dose.
Grade 4<br>(Life-threatening consequences; urgent intervention indicated)	Discontinue everolimus.
Any hematologic toxicity requiring study drug interruption for >28 days	Discontinue everolimus.
**Hepatic**	
**Isolated AST or ALT elevation**	
> ULN - 3.0 × ULN	Maintain dose level

[redacted]

**Recommended dose modifications for everolimus/placebo**	
Worst toxicity<br>CTCAE Grade (value)	During a cycle of therapy
> 3.0 - 5.0 × ULN<br>For patients with baseline value ≤ 3.0 × ULN	Maintain dose level. Repeat LFTs[b] as soon as possible, preferably within 48-72 hours from awareness of the abnormal results; if abnormal lab values are confirmed upon the repeat test, then monitor LFTs[b] weekly, or more frequently if clinically indicated, until resolved to ≤ 3.0 × ULN
For patients with baseline value > 3.0 -5.0 × ULN	Maintain dose level
> 5.0 - 10.0 × ULN<br>For patients with baseline value ≤ 3.0 × ULN	Omit dose. Repeat LFTs[b] as soon as possible, preferably within 48-72 hours from awareness of the abnormal results; monitor LFTs[b] weekly, or more frequently if clinically indicated, until resolved to ≤ 3.0 × ULN. Then<br>If resolved in ≤ 14 days, maintain dose level<br>If resolved in > 14 days, ↓ 1 dose level
For patients with baseline value > 3.0 -5.0 × ULN	Maintain dose level. Repeat LFTs[b] as soon as possible, preferably within 48-72 hours from awareness of the abnormal results; if abnormal lab values are confirmed upon the repeat test, then monitor LFTs[b], weekly, or more frequently if clinically indicated, until resolved to ≤ 5.0 × ULN
> 10.0 - 20.0 × ULN	Omit dose. Repeat LFTs[b] as soon as possible, preferably within 48-72 hours from awareness of the abnormal results; monitor LFTs[b] weekly, or more frequently if clinically indicated, until resolved to ≤ baseline. Then ↓ 1 dose level.
> 20.0 × ULN	Omit dose. Repeat LFTs[b] as soon as possible, preferably within 48-72 hours from awareness of the abnormal results; monitor LFTs[b] weekly, or more frequently if clinically indicated, until resolved to ≤ 3 x ULN (or ≤ 5 × ULN for patients with baseline value > 3.0 -5.0 × ULN), then resume treatment at ↓ 1 dose level. Only 1 dose reduction is allowed; if reoccurs at > 5 × ULN, discontinue patient from study treatment.
**Combined [c] elevations of AST or ALT and total bilirubin**	
For patients with normal baseline ALT and AST and total bilirubin value:<br>AST or ALT >3.0xULN combined with total bilirubin >2.0 x ULN without evidence of cholestasis[d]<br>OR<br>(Note to study team: If supported by available data)<br>For patients with elevated baseline AST or ALT or total bilirubin value<br>[AST or ALT>2x baseline AND > 3.0 xULN] OR [AST or ALT > 8.0 xULN], combined with [total bilirubin >2x baseline AND >2.0 ×ULN]	Permanently discontinue patient from study treatment.<br><br>Repeat as soon as possible, preferably within 48 hours from awareness of the abnormal results, then with weekly monitoring of LFTs[b]), or more frequently if clinically indicated, until AST, ALT, or bilirubin have resolved to baseline or stabilization over 4 weeks. Refer to Section 6.3.2.1 for additional follow-up evaluations as applicable.
**Metabolic** (e.g. hyperglycemia, dyslipidemia)	
Grade 1	Maintain dose level, initiate appropriate medical therapy and monitor
Grade 2	Maintain dose level, manage with appropriate medical therapy and monitor
Grade 3	Omit dose until resolved to ≤ grade 1, then ↓ 1 dose level
Grade 4	Discontinue patient from study treatment, and treat with appropriate medical

[redacted]

**Recommended dose modifications for everolimus/placebo**	
Worst toxicity<br>CTCAE Grade (value)	During a cycle of therapy
**Non-infectious pneumonitis**	See Section 6.3.2.1.
**Stomatitis**	See Section 6.3.2.2.
Other non-hematologic toxicities (excluding metabolic events)	
Grade 1	If toxicity is tolerable, no dose adjustment required.<br>Initiate appropriate medical therapy and monitor.
Grade 2	If toxicity is tolerable, no dose adjustment required.<br>Initiate appropriate medical therapy and monitor.<br>If toxicity becomes intolerable, temporary dose interruption until recovery to Grade ≤1. Re-initiate everolimus at the same dose.<br>If toxicity recurs at Grade 2, interrupt everolimus until recovery to Grade ≤1. Re-initiate everolimus at a lower dose.
Grade 3	Temporary dose interruption until recovery to Grade ≤1.<br>Initiate appropriate medical therapy and monitor.<br>Consider re-initiating everolimus at a lower dose.<br>If toxicity recurs at Grade 3, consider discontinuation.
Grade 4	Discontinue everolimus and treat with appropriate medical therapy.
All dose modifications should be based on the worst preceding toxicity.<br>[a] Common Toxicity Criteria for Adverse Events (CTCAE Version 4.03)<br>[b] Core LFTs consist of ALT, AST, GGT, total bilirubin (fractionated [direct and indirect], if total bilirubin > 2.0 × ULN), and alkaline phosphatase (fractionated [quantification of isoforms], if alkaline phosphatase > 2.0 × ULN.)<br>[c] "Combined" defined as total bilirubin increase to the defined threshold concurrently with ALT/AST increase to the defined threshold<br>If combined elevations of AST or ALT and total bilirubin do not meet the defined thresholds, please follow the instructions for isolated elevation of total bilirubin and isolated elevation of AST/ALT, and take a conservative action based on the degree of the elevations (e.g. discontinue treatment at the situation when omit dose is needed for one parameter and discontinue treatment is required for another parameter). After all elevations resolve to the defined thresholds that allow treatment re-initiation, re-start the treatment either at the same dose or at one dose lower if meeting a criterion for dose reduction	

6.3.2 Additional management and follow-up for selected toxicities

6.3.2.1 Management of infections

Everolimus has immunosuppressive properties and may predispose patients to bacterial, fungal, viral or protozoal infections, including infections with opportunistic pathogens. Localized and systemic infections, including pneumonia, other bacterial infections, invasive fungal infections, such as aspergillosis, candidiasis or pneumocystis jirovecii pneumonia (PJP) and viral infections including reactivation of hepatitis B virus, have been described in patients taking everolimus. Some of these infections have been severe (e.g. leading to sepsis, respiratory or hepatic failure) and occasionally have had a fatal outcome.

Physicians and patients should be aware of the increased risk of infection with everolimus. Treat pre-existing infections prior to starting treatment with everolimus. While taking everolimus, be vigilant for symptoms and signs of infection; if a diagnosis of infection is made, institute appropriate treatment promptly and consider interruption or discontinuation of everolimus.

[redacted]

Patients with positive results of HBV-DNA and/or HBsAg at screening should begin a prophylaxis treatment for 1-2 week prior to beginning everolimus therapy. Patients should have HBV-DNA monitored frequently throughout the course of everolimus therapy for signs of hepatitis reactivation.

If a diagnosis of invasive systemic fungal infection is made, discontinue everolimus and treat with appropriate antifungal therapy.

Cases of PJP, some with fatal outcome, have been reported in patients who received everolimus. PJP may be associated with concomitant use of corticosteroids or other immunosuppressive agents. Prophylaxis for PJP should be considered when concomitant use of corticosteroids or other immunosuppressive agents is required.

6.3.2.2 Management of non-infectious pneumonitis

Non-infectious pneumonitis is a known side effect of rapamycin analogues. Clinically significant pneumonitis is typically accompanied by non-specific symptoms including dyspnea, nonproductive cough, fatigue, and fever. Diagnosis is generally suspected in individuals receiving mTOR inhibitors who develop these symptoms or in asymptomatic individuals in whom a routine chest CT scan reveals a new ground glass or alveolar infiltrate.

The frequency of symptomatic pulmonary toxicity (all grades) was approximately 13% in a phase III study of everolimus in patients with metastatic renal cell carcinoma [CRAD001C2240]. Severe (CTC grade 3) pneumonitis occurred in 4% of patients, and an occasional fatality was reported. The lung toxicity was partly or completely reversible in the majority of cases with interventions including drug interruption, discontinuation and the use of corticosteroids.

Individuals participating in this trial will be routinely questioned as to the presence of new or changed pulmonary symptoms consistent with lung toxicity. CT scans and pulmonary function test should be done, as clinically indicated, if there are symptoms that indicate that the patient has developed non-infectious pneumonitis. If non-infectious pneumonitis develops, the guidelines in Table 6-4 should be followed. Dose modification instructions are also provided in Table 6-4. Consultation with a pulmonologist is recommended for any case of pneumonitis that develops during the study.

[redacted]

Table 6-4 Management of non-infectious pneumonitis

Worst Grade Pneumonitis	Required Investigations	Management of Pneumonitis	EVEROLIMUS/placebo Dose Adjustment
Grade 1	CT scans with lung windows. Repeat at least every 8 weeks until return to within normal limits.	No specific therapy is required	Administer 100% of study treatment dose.
Grade 2	CT scan with lung windows. Consider pulmonary function testing includes: spirometry, DLCO, and room air O^2 saturation at rest. Repeat at least every 8 weeks until return to within normal limits. Consider a bronchoscopy with biopsy and / or BAL.	Symptomatic only. Consider corticosteroids if symptoms are troublesome.	Reduce study treatment dose by 1 dose level (see Table 6-2) until recovery to < Grade 1. Study treatment may also be interrupted if symptoms are troublesome. Patients will discontinue study treatment if they fail to recover to < Grade 1 within 28 days.
Grade 3	CT scan with lung windows pulmonary function testing includes: spirometry, DLCO, and room air O^2 saturation at rest. Repeat at least every 6 weeks until return to within normal limits. Bronchoscopy with biopsy and / or BAL is recommended.	Consider corticosteroids if infective origin is ruled out. Taper as medically indicated.	Hold treatment until recovery to < Grade 1. May restart study treatment within 28 days at a reduced dose (by one level) if evidence of clinical benefit.
Grade 4	CT scan with lung windows and required pulmonary function testing, if possible, includes: spirometry, DLCO, and room air O2 saturation at rest. Repeat at least every 6 weeks until return to within normal limits. Bronchoscopy with biopsy and / or BAL is recommended if possible.	Consider corticosteroids if infective origin is ruled out. Taper as medically indicated.	Discontinue treatment.

6.3.2.3 Guidelines for the prevention and the treatment of study drug induced stomatitis/oral mucositis/mouth ulcers

For prevention the stomatitis, all patients will be instructed to perform routine "good oral care" each day during the trial. Good oral care will consist of: brushing teeth at least twice daily with soft bristled toothbrush, continue current daily flossing routine (if patients were not already flossing daily, they should not be instructed to start flossing as this could cause oral trauma), and continue routine dental care/maintenance with their dentist, if they have one. It is' recommended that patients should use 10mL of an alcohol-free, 0.5mg/5mL dexamethasone steroid mouthwash swishing and spitting QID, especially during the first 8 weeks of treatment (majority of stomatitis events occur within the first 8 weeks of treatment). The mouthwash is to be held in mouth and swished around mouth to come in contact with entire buccal mucosa surface for a minimum of two minutes, and then spat out.

Patients with a clinical history of stomatitis/mucositis/mouth ulcers and those with gastrointestinal morbidity associated with mouth/dental infections, irritation of esophageal

[redacted]

mucosa e.g. gastroesophageal reflux disease (GERD) and pre-existing stomatitis/mucositis must be monitored even more closely. Patients should be instructed to report the first onset of buccal mucosa irritation/reddening to their study physician immediately.

General guidance and management include patient awareness and early intervention. Stomatitis/oral mucositis/mouth ulcers due to everolimus should be treated using local supportive care. Evaluation for herpes virus or fungal infection should be considered. Patients should be informed about the possibility of developing mouth ulcers/ oral mucositis and instructed to report promptly any signs or symptoms to their physician. Patients should be educated about good oral hygiene, instructed to avoid spicy/acidic/salty foods, and should follow the following guidelines:

- For mild toxicity (grade 1), use conservative measures such as non-alcoholic mouth wash or salt water (0.9%) mouth wash several times a day until resolution.
- For more severe toxicity (grade 2 in which case patients have pain but are able to maintain adequate oral alimentation, or grade 3 in which case patients cannot maintain adequate oral alimentation), the suggested treatments are topical analgesic mouth treatments (i.e., local anesthetics such as benzocaine, butyl aminobenzoate, tetracaine hydrochloride, menthol, or phenol) with or without topical corticosteroids, such as triamcinolone oral paste 0.1% (Kenalog in Orabase®).
- Agents containing alcohol, hydrogen peroxide, iodine, and thyme derivatives may tend to worsen mouth ulcers. It is preferable to avoid these agents.
- Antifungal agents must be avoided unless a fungal infection is diagnosed. In particular, systemic imidazole antifungal agents (ketoconazole, fluconazole, itraconazole, etc.) should be avoided in all patients due to their strong inhibition of everolimus metabolism, therefore leading to higher everolimus exposures. Therefore, topical antifungal agents are preferred if an infection is diagnosed. Similarly, antiviral agents such as acyclovir should be avoided unless a viral infection is diagnosed.

6.3.2.4 Management of hepatitis reactivation

Both chemotherapy (Yeo et al 2004) and mTOR inhibition (Guo et al 2007) may cause reactivation of hepatitis B.

Table 6-5 provides details of monitoring and prophylactic therapy according to the screening results of viral load and serologic markers testing. If the patient is already known to have a chronic infection with HBV and is taking anti-HBV medication, the site does not have to wait for the screening HBV results from the local laboratory prior to the first dose of the study treatment.

[redacted]

Table 6-5 Action to be taken based on hepatitis B screening results

	Result	Result	Result	Result	Result
HBV-DNA	+	+ or -	-	-	-
HBsAg	+ or -	+	-	-	-
HBsAb	+ or -	+ or -	+<br>and no prior HBV vaccination	+ or -	- or +<br>with prior HBV vaccination
HBcAb	+ or -	+ or -	+ or -	+	-
Recommendation	Prophylaxis treatment should be started 1-2 weeks prior to first dose of study treatment.<br>Monitor HBV-DNA approximately every 4-8 weeks<br>(from Visit 2 and onwards)		No prophylaxis<br>Monitor HBV-DNA approximately every 4 weeks<br>(from Visit 2 and onwards)		No specific action

Antiviral prophylaxis therapy should continue for at least 4 weeks after last dose of study treatment. For hepatitis B reactivation, definition and management guidelines see Table 6-6.

Table 6-6 Guidelines for management of hepatitis B

**HBV reactivation** (with or without clinical signs and symptoms)*	
For patients with baseline results:<br>Positive HBV-DNA OR<br>Positive HBsAg<br>--------------------------------------<br>Reactivation is defined as:<br>[Increase of 1 log in HBV-DNA relative to baseline HBV-DNA value OR new appearance of measurable HBV-DNA]	Treat: Start a second antiviral<br>AND<br>Interrupt study treatment administration until resolution:<br>-<br>≤ baseline HBV-DNA levels<br><br>If resolution occurs within 28 days, study treatment should be re-started at one dose lower, if available. (see Table 6-2 Study drug dose reductions) If the patient is already receiving the lowest dose of study drug according to the protocol, the patient should restart at the same dose after resolution. Both antiviral therapies should continue at least 4 weeks after last dose of study drug.<br>If resolution occurs > 28 days, patients should discontinue study treatment but continue both antiviral therapies at least 4 weeks after last dose of study drug.
For patients with baseline results:<br>Negative HBV-DNA and HBsAg<br>AND<br>[Positive HBsAb (with no prior history of vaccination against HBV), OR positive HBcAb]<br>-------------------------------------<br>reactivation is defined as: New appearance of measurable HBV-DNA	Treat : Start first antiviral medication<br>AND<br>Interrupt study drug administration until resolution:<br>≤ undetectable (negative) HBV-DNA levels<br>If resolution occurs within 28 days, study drug should be re-started at one dose lower, if available. (see Table 6-2 Study drug dose reductions) If the patient is already receiving the lowest dose of study drug according to the protocol, the patient should restart at the same dose after resolution. Antiviral therapy should continue at least 4 weeks after last dose of study drug.<br>If resolution occurs > 28 days, patients should discontinue study drug but continue antiviral therapy at least 4 weeks after last dose of study drug.
* All reactivations of hepatitis B are to be recorded as grade 3 (CTCAE v4.03), unless considered life threatening by the investigator; in which case they should be recorded as grade 4 (CTCAE v4.03). Date of viral reactivation is the date on which the rise or reappearance of HBV- DNA was recorded.	

[redacted]

Monitoring for hepatitis C flare

The following two categories of patients should be monitored every 8 weeks for HCV flare:

- Patients with detectable HCV RNA-PCR test at screening.
- Patients known to have a history of HCV infection, despite a negative viral load test at screening (including those that were treated and are considered 'cured')

For definition of HCV flare and the management guidelines, see Table 6-7 Guidelines for management of hepatitis C. If the patient is already known to have a chronic infection with HCV, the site does not have to wait for the screening HCV results from the local laboratory prior to the first dose of the study treatment.

Table 6-7 Guidelines for management of hepatitis C flare

HCV flare *	
For patients with baseline results: Detectable HCV-RNA,<br>HCV flare is defined as:<br>> 2 log_{10} IU/mL increase in HCV-RNA AND<br>ALT elevation > 5 x ULN OR 3 x baseline level, whichever is higher	Discontinue study treatment
For patients with baseline results:<br>Knowledge of past hepatitis C infection with no detectable HCV-RNA,<br>HCV flare is defined as:<br>New appearance of detectable HCV-RNA AND<br>ALT elevation > 5 × ULN OR 3 x baseline level, whichever is higher	Discontinue study treatment
*All flares of hepatitis C are to be recorded as grade 3 (CTCAE v 4.03), unless considered life threatening by the investigator; in which case they should be recorded as grade 4 (CTCAE v 4.03). Date of viral flare is the date on which both the clinical criteria described above were met (e.g., for a patient whose HCV-RNA increased by 2 logs on 01 JAN 2011 and whose ALT reached > 5 × ULN on 22 JAN 2011), the date of viral flare is 22 JAN 2011.	

6.3.2.5 Management of skin toxicity

For patients with grade 1 toxicity, no specific supportive care is usually needed or indicated. Rash must be reported as an AE. Patients with grade 2 or higher toxicity may be treated with the following suggested supportive measures at the discretion of the investigator: oral minocycline, topical tetracycline, topical clindamycin, topical silver sulfadiazine, diphenphydramine, oral prednisolone (short course) topical corticosteroids or pimecrolimus.

6.3.2.6 Management of haemorrhage

Caution is advised in patients taking everolimus particularly during concomitant use with active substances known to affect platelet function or that can increase the risk of haemorrhage as well as in patients with a history of bleeding disorders. Healthcare professionals and patients should be vigilant for signs and symptoms of bleeding throughout the treatment period, especially if risk factors for haemorrhage are combined.

[redacted]

6.3.2.7 Management of wound healing complications

Impaired wound healing is a class effect of rapamycin derivatives, including everolimus. Caution should therefore be exercised with the use of everolimus in the peri-surgical period.

6.3.2.8 Management of lactose

Patients with rare hereditary problems of galactose intolerance, Lapp lactase deficiency or glucose-galactose malabsorption should not take this medicinal product.

6.3.2.9 Management of hyperlipidemia and hyperglycemia

Treatment of hyperlipidemia should take into account the pre-treatment status and dietary habits of the patient. Grade 2 or higher hypercholesterolemia (>300 mg/dL or 7.75 mmol/L) or grade 2 hypertriglyceridemia or higher (>2.5x upper normal limit) should be treated with a 3-hydroxy-3-methyl-glutaryl (HMG)-CoA reductase inhibitor (e.g. atorvastatin, pravastatin, fluvastatin) or appropriate triglyceride-lowering medication, in addition to diet.

Note: Concomitant therapy with fibrates and an HMG-CoA reductase inhibitor is associated with an increased risk of a rare but serious skeletal muscle toxicity manifested by rhabdomyolysis, markedly elevated creatine phosphokinase (CPK) levels and myoglobinuria, acute renal failure and sometimes death. The risk versus benefit of using this therapy should be determined for individual patients based on their risk of cardiovascular complications of hyperlipidemia.

Hyperglycemia has been reported in patients taking everolimus. Monitoring of fasting serum glucose is recommended prior to the start of study drug and periodically thereafter. More frequent monitoring is recommended when everolimus is co-administered with other drugs that may induce hyperglycemia. Optimal glycemic control should be achieved before starting a patient on study drug.

6.3.2.10 Management of renal failure events

Cases of renal failure (including acute renal failure), some with a fatal outcome, have been observed in patients treated with everolimus. Renal function of patients should be monitored particularly where patients have additional risk factors that may further impair renal function. Monitoring of renal function, including measurement of blood urea nitrogen (BUN), urinary protein, or serum creatinine, is recommended prior to the start of study drug and periodically thereafter.

6.3.3 Exemestane

The most frequently reported adverse effects for exemestane are gastrointestinal disturbances, hot flushes, arthralgia, myalgia, sweating, fatigue, and dizziness. Other reported effects include headache, insomnia, somnolence, depression, skin rashes, alopecia, asthenia, and peripheral and leg oedema. Thrombocytopenia and leucopenia have been reported occasionally. Reductions in bone mineral density can occur with long-term use of exemestane.

Exemestane was generally well tolerated, and adverse events were usually mild to moderate. Adverse events occurring in greater than 10% of patients include hot flushes (14%), nausea (11.9%), insomnia, headache, increased sweating, joint and musculoskeletal pain, and fatigue

[redacted]

(USPI; Aromasin SmPC August 2008 (UK as RMS for EU MRP)). Androgenic effects were reported in a limited number (4.3%) of patients (Buzdar 2003).

Refer to the package insert of the local supply of exemestane for more details.

6.3.4 Follow-up for toxicities

Patients whose treatment is interrupted or permanently discontinued due to an adverse event must be followed at least once a week for 4 weeks, and subsequently at 4-week intervals, until resolution or stabilization of the event. All patients will be followed for onset of any new serious adverse events for 30 days following the last dose of study treatment.

If, because of toxicity, a patient requires a dose delay of > 4 weeks from the intended day of dose, then the patient must discontinue study treatment. However, the patient will continue to be followed for toxicity and tumor assessments as previously described.

6.4 Concomitant medications

All medications (excluding study treatment and prior antineoplastic treatments), procedures and significant non-drug therapies (including physical therapy and blood transfusions) administered within 28 days prior to the study treatment through 30 days after the last dose of study treatment (either everolimus/placebo or exemestane, whichever is later) will be recorded in the Concomitant medications or Surgical and medical procedures eCRF. Medications include not only physician prescribed medications, but also all over-the counter medications, herbal medications (prohibited) and food or vitamin supplements. The investigator should instruct the patient to notify the investigational site about any new medications she takes after the start of the study treatment.

The patient must be told to notify the investigational site about any new medications he/she takes after the start of the study treatment. All medications (other than study treatment) and significant non-drug therapies (including physical therapy and blood transfusions) administered during the study must be listed on the Concomitant Medications or the Procedures and Significant Non-Drug Therapies eCRF, respectively.

6.4.1 Prohibited concomitant therapy

The following concomitant treatments are not allowed during the study:

- Investigational or commercial anticancer agents, such as chemotherapy, immunotherapy, targeted therapy, or endocrine therapy should not be given to patients.
- Hormone replacement therapy, topical estrogens (including any intra-vaginal preparations), megestrol acetate and selective estrogen-receptor modulators (e.g. raloxifene) are prohibited.
- Prolonged systemic corticosteroid treatment, except for topical applications (e.g. rash), inhaled sprays (e.g. obstructive airways diseases), eye drops or local injections (e.g. intraarticular) should not be given. A short duration (<2 weeks) of systemic corticosteroids is allowed (e.g. chronic obstructive pulmonary disease, anti-emetic).

[redacted]

- Hematopoietic growth factors (e.g. erythropoietins, G-CSF and GM-CSF) are not to be administered prophylactically. Use of these should be reserved to cases of severe neutropenia and anemia as per the labeling of these agents.

It is to be noted that

- Local radiotherapy for analgesic purposes or for lytic lesions at risk of fracture may be carried out if required. Whenever possible, these patients should have a tumor assessment of the lesion(s) before they actually receive the radiotherapy. No dose modification of study treatment is needed during radiotherapy.
- Everolimus may affect the response to vaccinations making it less effective. Live vaccines should be avoided while a patient is treated with everolimus.

6.4.2 Use of bisphosphonates

The use of bisphosphonates for bone metastatic disease is allowed. If bisphosphonate therapy is initiated after start of study treatment, the reason for its use must be clearly documented and progression as per RECIST 1.1 must be ruled out.

Same guidelines should apply to the use of denosumab in the treatment of bone metastatic disease.

6.4.3 Other concomitant medications

Everolimus is a substrate of CYP3A4, and a substrate and moderate inhibitor of the multidrug efflux pump, PgP (PgP, MDR1, and ABCB1). Therefore, extent of absorption and subsequent elimination of systemically absorbed everolimus may be influenced by products that are substrates, inhibitors, or inducers of CYP3A4 and/or PgP. Co-administration with strong inhibitors of CYP3A4 or PgP should be avoided. Co-administration with moderate CYP3A4 inhibitors (e.g., erythromycin, fluconazole) or PgP inhibitors should be used with caution. Seville orange, star fruit, grapefruit and their juices affect CYP3A4 and PgP activity. Concomitant use should be avoided.

Please refer to Table 6-8 listing relevant inducers and inhibitors of CYP3A and to Table 6-9 for a list of relevant substrates, inducers, and inhibitors of PgP.

Everolimus may affect the response to vaccinations making the response to the vaccination less effective. Live vaccines should be avoided while a patient is treated with everolimus.

[redacted]

Table 6-8 Clinically relevant drug interactions: inducers, and inhibitors of isoenzyme CYP3A

**Inducers**
**Strong inducers:**
avasimibe, carbamazepine, mitotane, phenobarbital, phenytoin, rifabutin, rifampin (rifampicin), St. John's wort (hypericum perforatum)
**Moderate inducers:**
bosentan, efavirenz, etravirine, modafinil, nafcillin, genistein, ritonavir, thioridazine, tipranavir, semagacestat, talviraline, lopinavir, lersivirine,
**Weak inducers:**
amprenavir, aprepitant, armodafinil (R-modafinil), bexarotene, clobazam, danshen, dexamethasone, Echinacea, garlic (allium sativum), gingko (ginkgo biloba), glycyrrhizin, methylprednisolone, nevirapine, oxcarbazepine, pioglitazone, prednisone, [pleconaril], primidone, raltegravir, rufinamide, sorafenib, telaprevir, terbinafine, topiramate, [troglitazone], vinblastine eslicarbazepine, ginseng, vemurafenib, boceprevir, sulfinpyrazone, ticagleror, vicriviroc/ritonavir, ritonavir, ticlopidine, brivacetam, Stribild (combo of elvitegravir, cobicistat, emtricitabine, and tenofovir), quercetin.
**Inhibitors**
**Strong inhibitors:**
**boceprevir**, clarithromycin, cobicistat, conivaptan, elvitegravir, indinavir, itraconazole, ketoconazole, lopinavir, mibefradil, nefazodone, nelfinavir, posaconazole (Krishna et al 2009), ritonavir, saquinavir, **telaprevir**, telithromycin, tipranavir, troleandamycin, voriconazole, indinavir/ritonavir, tipranoavir/ritonavir, cobicistat, troleandomycin, danoprevir/ritonavir, eltegravir/ritonavir,
**Moderate inhibitors:**
amprenavir, aprepitant, atazanavir, ciprofloxacin, darunavir/ritonavir, diltiazem, erythromycin, fluconazole, fosamprenavir, grapefruit juice, nilotinib imatinib, tofisopam, cyclosporin, ciprofloxacin, verapamil, dronedarone, crizotinib, casopitant, amprenavir, atazanavir/ritonavir, duranavir, netupitant, schisandra sphenanthera, cimetidine, lomitapide
**Weak inhibitors:**
tabimorelin, ranolaxine, fosaprepritant, Seville orange, amlodipine, clozoxazone, fluvoxamine, ranitidine, goldenseal, clotrimazole, tacrolimus, cilostazol, ticagrelor, ivacaftor, roxithromycin, propiverine, isoniazid, berberine, oral contraceptives, peppermint oil, delavirdine, simeprevir, atorvastatin, tolvaptan, almorexant, linagliptin, resveratrol, lacipidine, cranberry juice, nilotinib, pazopanib, evrolimus, blueberry juice, alprazolam, bicalutamide, sitaxentan, azithromycin, ginkgo, teriflunomide, alprazolam, amiodarone, amlodipine, bicalutamide, cilostazol, cimetidine, cyclosporine, fluoxetine, isoniazid, ranitidine, ranolazine, tipranavir/ritonavir, zileuton

[redacted]

Table 6-9 Clinically relevant drug interactions: substrates, inducers, inhibitors of PgP and PgP/CYP3A dual inhibitors

**Substrates**
digoxin, quinidine, paclitaxel, cyclosporine, sirolimus, tacrolimus, fentanyl, pphenytoin, aliskiren, ambrisentan, atorvastatin, atorvastatin acid, azithromycin, cerivastatin, colchicine, CP-481,715, cyclosporine, dabigatran, digoxin, docetaxel, domperidone, doxorubicin, fentanyl, fexofenadine, lapatinib, linezolid, loperamide, maraviroc, nevirapine, paclitaxel, proguanil, quinidine, ranolazine, ritonavir, saquinavir, simvastatin, sirolimus, sofosbuvir, tacrolimus, ticagrelor, voclosporin, afatinib, alfuzosin, aliskiren, alogliptin, ambrisentan, apixaban, apremilast, aprepitant, boceprevir, bosentan, carvedilol, carvedilol, caspofungin, ceritinib, citalopram, colchicine, cyclosporine, dabigatran, digoxin, doxepin, doxorubicin, eribulin, everolimus, fidaxomicin, fluvastatin, fosamprenavir, gatifloxacin, idelalisib, iloperidone, indacaterol, irbesartan, lacosamide, lapatinib, levetiracetam, levofloxacin, linagliptin, losartan, maraviroc, mirabegron, moxifloxacin, naloxegol, nateglinide, nintedanib, olodaterol, pantoprazole, paroxetine, pazopanib, posaconazole, pravastatin, quinine, ranolazine, riociguat, risperidone, rivaroxaban, saquinavir, silodosin, simeprevir, sirolimus, sitagliptin, sorafenib, telaprevir, tenofovir, ticagrelor, tipranavir, tolvaptan, topotecan, umeclidinium, valsartan, vardenafil, vincristine, voriconazole
**Inducers**
avasimibe, carbamazepine, efavirenz, genistein, phenytoin, quercetin, rifampin, St. Johns wort extract
**PgP Inhibitors and PgP/CYP3A Dual Inhibitors**
PgP Inhibitors<br>alogliptin, canaglifozin, cremophor RH40, curcumin, ketoconazole, lapatinib, lopinavir/ritonavir, mirabegron, propafenone, simepravir, valspodar, vandetanib, voclosporin<br>PgP/CYP3A Dual Inhibitors<br>amiodarone, azithromycin,captopril, carvedilol, clarithromycin, conivaptan, diltiazem, dronedarone, elacridar, erythromycin, felodipine, fluvoxamine, ginko, indinavir, indinavir/ritonavir, itraconazole, mibefradil, milk thisle, nelfinavir, nifedipine, nitredipine, paroxetine, quercetin, quinidine, ranolazine, rifampin, ritonavir, sequinavir/ritonavir, schisandra chinesis extract, St. John's wort extract, talinolol, telaprevir, telmisartan, ticagrelor, tipranavir/ritonavir, tolvaptan, verapamil
Reference: Internal Clinical Pharmacology Drug-drug interaction (DDI) memo, updated April-2015 which summarizes DDI data from three sources including the FDA's "Guidance for Industry, Drug Interaction Studies", the University of Washington's Drug Interaction Database, and Indiana University School of Medicine's Drug Interaction Table.

Vaccinations

Immunosuppressants may affect the response to vaccination and vaccination during treatment with everolimus may therefore be less effective. The use of live vaccines should be avoided during treatment with everolimus.

Examples of live vaccines are: intranasal influenza, measles, mumps, rubella, oral polio, BCG, yellow fever, varicella, and TY21a typhoid vaccines.

6.5 Patient numbering, treatment assignment or randomization

6.5.1 Patient numbering

Each patient is identified in the study by a Subject number, that is assigned when the patient is first enrolled for screening and is retained as the primary identifier for the patient throughout his/her entire participation in the trial. The Subject number consists of the Center Number (Center No.) which is assigned by Novartis to the investigative site with a sequential patient number suffixed to it, so that each subject is numbered uniquely across the entire database. Upon signing the informed consent form, the patient is assigned to the next sequential Subject number available to the investigator.

[redacted]

The investigator or designated staff will contact the IRT and provide the requested identifying information for the patient to register them into the IRT. Once assigned, the Subject number must not be reused for any other subject and the Subject number for that individual must not be changed, even if the patient is re-screened. If the patient fails to be randomized or start treatment for any reason, the reason will be entered into the Screening Disposition page.

6.5.2 Treatment assignment or randomization

Patients will be assigned to one of the two treatment arms (Section 4.1 and Section 6.1) in a ratio of “1:1”. Randomization will be stratified by documented sensitivity to prior hormonal therapy (yes vs. no) and by the presence of visceral disease (yes vs. no).

The randomization numbers will be generated using the following procedure to ensure that treatment assignment is unbiased and concealed from patients and investigator staff. A patient randomization list will be produced by the Interactive Response Technology (IRT) provider using a validated system that automates the random assignment of patient numbers to randomization numbers. These randomization numbers are linked to the different treatment arms, which in turn are linked to medication numbers.

Prior to dosing, all patients who fulfill all inclusion/exclusion criteria will be randomized via IRT to one of the treatment arms. The investigator or his/her delegate will call or log on to the IRT and confirm that the patient fulfills all the inclusion/exclusion criteria. The IRT will assign a randomization number to the patient, which will be used to link the patient to a treatment arm and will specify a unique medication number for the first package of study treatment to be dispensed to the patient. The randomization number will not be communicated to the caller.

6.5.3 Treatment blinding

This is a double blind study. In particular, patients, investigators, local radiologists, study team, or anyone involved in the study conduct will remain blinded to the identity of the treatment from the time of randomization until unblinding.

Randomization data are kept strictly confidential until the time of unblinding, and will not be accessible to anyone involved in the conduct of the study. The identity of the treatments will be concealed by the use of investigational drugs (everolimus or placebo) that are identical in packaging, labeling, schedule of administration and in appearance. Confidentiality of randomization data is required to limit the occurrence of potential bias arising from the influence that the knowledge of treatment may have on the recruitment and allocation of patients.

Unblinding will only occur in the case of patient emergencies (Section 8.3), for regulatory reporting purposes and at the conclusion of the study.

In rare cases when unblinding occurs because of emergency patient management, the actual treatment arm will not be communicated to any of the Novartis employees involved in running the trial in order to remain blinded. The patient will be withdrawn from the study treatment.

[redacted]

At the conclusion of the study, when the study data have been verified, and the protocol deviations have been determined and the database locked, the assigned blinded drug codes can be broken and made available to the Sponsor for the final analysis of the study data.

6.6 Study treatment preparation and dispensation

The investigator or responsible site personnel must instruct the patient or caregiver to take the study treatment as per protocol. Study treatment will be dispensed to the patient by authorized site personnel only. All dosages prescribed to the patient and all dose changes during the study must be recorded on the Dosage Administration Record CRF.

Tablets of everolimus and exemestane including instructions for administration are dispensed by study personnel on an outpatient basis.

Patients will be provided with adequate supply of study treatment for self-administration at home until at least their next scheduled study visit.

6.6.1 Study treatment packaging and labeling

Responsible site personnel will dispense everolimus/placebo to the patient by using the IRT and obtaining the medication number(s). Site personnel will add the patient number on the label. Everolimus/placebo labels will be in the local language and comply with the legal requirements of China. They will include storage conditions for the drug and the medication number but no information about the patient.

Commercially available exemestane will be supplied to investigator sites in accordance with local regulations in China.

Table 6-10 Packaging and labeling

Study treatments	Packaging	Labeling (and dosing frequency)
Everolimus/placebo	Tablets in blister	Labeled as 'RAD001', 2×5mg tablets to be taken orally
Exemestane	Refer to local product information	Refer to local product information

6.6.2 Drug supply and storage

Study treatments must be received by designated personnel at the study site, handled and stored safely and properly, and kept in a secured location to which only the investigator and designated site personnel have access. Upon receipt, the study treatment should be stored according to the instructions specified on the drug labels and in the [Investigator's Brochure].

Table 6-11 Supply and storage of study treatments

Study treatments	Supply	Storage
Everolimus/placebo	Locally	Refer to study treatment label
Exemestane	Locally	Refer to local product information

[redacted]

6.6.3 Study treatment compliance and accountability

6.6.3.1 Study treatment compliance

Compliance will be assessed by the investigator and/or study personnel at each patient visit and information provided by the patient and/or caregiver will be captured in the Drug Accountability Form. This information must be captured in the source document at each patient visit.

6.6.3.2 Study treatment accountability

The investigator or designee must maintain an accurate record of the shipment and dispensing of study treatment in a drug accountability log. Drug accountability will be noted by the field monitor during site visits and at the completion of the study. Patients will be asked to return all unused study treatment and packaging on a regular basis, at the end of the study or at the time of study treatment discontinuation.

At study close-out, and, as appropriate during the course of the study, the investigator will return all used and unused study treatment, packaging, drug labels, and a copy of the completed drug accountability log to the Novartis monitor or to the Novartis address provided in the investigator folder at each site.

6.6.4 Disposal and destruction

The study treatment supply can be destroyed at the local Novartis facility, Drug Supply group or third party, as appropriate.

7 Visit schedule and assessments

7.1 Study flow and visit schedule

Table 7-1 lists all of the assessments and indicates with an “X”, the visits when they are performed. All data obtained from these assessments must be supported in the patient’s source documentation. The table indicates which data are entered into the database (D) or remain in source documents only (S). Assessments that generate data for database entry and which are recorded on eCRFs are listed using the eCRF name.

No CRF will be used as a source document.

[redacted]

Table 7-1 Visit evaluation schedule

				**Randomized treatment phase**			**Post Treatment Follow up phase**	**Survival follow up phase**
	**Category**	**Protocol Section**	**Screening/ Baseline**	**Cycle 1**	**Every 2 cycles after C1D1**	**End of study treatment (EoT)**		
Visit Name			Screening Visit (D -21 to D -1)	C1D1	C3D1 C5D1, etc…	EOT Visit (within 7d of last dose)	POST TRT FUP 1, 2, ect	Survival fup
Obtain Informed Consent	D,S		X					
IRT screening / randomization / discontinuation / progression / death	S		X	X		X		
Patient history	D		X					
Demography	D		X					
Inclusion/exclusion criteria	D		X					
**Medical History**	D		X					
Diagnosis and extent of cancer	D		X					
Smoking history	D		X					
Prior/Post antineoplastic therapy	D		X			X		
Prior/concomitant medications	D		X	Continuous			up to 30 days after last dose of treatment	
Complete Physical examination	S	7.2.2.1.	X	If clinically indicated				
Performance status	D	7.2.2.4.	X	X	X	X		
Height	D	7.2.2.3.	X					
Weight	D	7.2.2.3.	X	X	X	X		
Vital signs	D	7.2.2.2.	X	X	X	X		

[redacted]

				**Randomized treatment phase**			**Post Treatment Follow up phase**	**Survival follow up phase**
	**Category**	**Protocol Section**	**Screening/ Baseline**	**Cycle 1**	**Every 2 cycles after C1D1**	**End of study treatment (EoT)**		
Visit Name			Screening Visit (D -21 to D -1)	C1D1	C3D1 C5D1, etc…	EOT Visit (within 7d of last dose)	POST TRT FUP 1, 2, ect	Survival fup
**Laboratory assessments**								
Hematology	D	7.2.2.5.1.	X	X	X	X		
Biochemistry	D	7.2.2.5.2.	X	X	X	X		
Lipid Panel	D		X	X	X	X		
Coagulation	D	7.2.2.5.	X	X	X	X		
Hepatitis B and C screening/monitoring(if applicable)	D		X		X	X		
Tumor evaluation (**EOT scan not required if previous scan was performed within ≤28 days)**	D	7.2.1.	X		Every 2 cycles after C1D1; the frequency will be changed to every 12 weeks after data cut-off for primary analysis and as clinically indicated	X	X If not progressed, every 2 cycles after EOT ; the frequency will be changed to every 12 weeks after data cut-off for primary analysis and as clinically indicated	
ECG	D	7.2.2.5.3.	X	If clinically indicated				
Pulmonary function test	D		If clinically indicated, and if warranted to exclude or manage non-infection pneumonitis					
**Safety**								
Adverse events	D		X	Continuous			Up to 30 days after last dose of treatment	
**PK blood sampling**[1]	D	7.2.3.	X[1a]	X[1,1a]				

[redacted]

				**Randomized treatment phase**			**Post Treatment Follow up phase**	**Survival follow up phase**
	**Category**	**Protocol Section**	**Screening/ Baseline**	**Cycle 1**	**Every 2 cycles after C1D1**	**End of study treatment (EoT)**		
Visit Name			Screening Visit (D -21 to D -1)	C1D1	C3D1 C5D1, etc…	EOT Visit (within 7d of last dose)	POST TRT FUP 1, 2, ect	Survival fup
**Study treatment administration**								
Everolimus / Placebo Dosing	D			Continuous daily dosing				
Exemestane Study Dosing	D			Continuous daily dosing				
**Survival Follow-up**	D							X every 12 weeks

1.Blood samples for everolimus and exemestane levels will be collected pre-dose and 2 hours post dose on Cycle 1 Week 4.
1a. Blood sample for Estradiol (E2) will be collected at screening or day 1 before study treatment and at Cycle 1 Week 4.
A cycle is defined as 4 weeks ; Visit assessments window is ± 7 days

[redacted]

7.1.1 Molecular pre-screening

Not applicable.

7.1.2 Screening

Written informed consent must be obtained before any study specific assessments are performed, including screening.

The screening assessments must be performed within maximum 21 days to confirm patient's eligibility and to the first study treatment dose of randomized treatment phase.

Laboratory, physical examination, performance status and vital signs assessments should be performed within 7 days before Cycle 1 Day 1.

Tumor evaluation should be performed within 21 days prior to study treatment start in the randomized treatment phase on cycle 1 day 1 except for bone scans as noted below.

As part of patient's routine disease care (prior to signing study inform consent), with proper documentation in patient's file, the following tumor assessments can be used:

- if bone scan was performed within 4 weeks prior cycle 1 day 1

or

- if tumor scans were performed within 21 days prior to cycle 1 day 1.

For laboratory evaluations used to determine eligibility, a repeated evaluation within the screening window is permitted for screening results out of the defined range. If the repeated laboratory result meets the criteria, that result may be used to determine eligibility. If the repeated laboratory result does not meet the criteria, the patient will be considered a screening failure.

Performance status, weight, and vital signs performed ≤ 7 days of the first dose of study treatment do not need to be repeated on Day 1 of Cycle 1.

7.1.2.1 Eligibility screening

Following registering in the IRT for screening, patient eligibility will be checked once all screening procedures are completed. The eligibility check will be embedded in the IRT system. Please refer and comply with detailed guidelines in the IRT manual.

Re-screening is allowed, only, for patients who meet all of the inclusion/exclusion criteria, but were not randomized due to one of the following administrative reasons:

- CT /MRI results have expired (>28 days prior to randomization) due to unexpected administrative issues
- Unexpected drug supply issues.

Procedure for re-screening is as follows:

- Site must register the patient as a screen failure in IRT
- Site is to send a re-screening request to the CRA within 7 days of the screen failure date
- Subject number will be the same as the initial screening

[redacted]

- Informed consent doesn't need to be resigned.
- Lab assessments needs to be to performed -7 or -1 days before randomization

Adverse events that begin or worsen after informed consent should be recorded in the Adverse Events CRF. Conditions that were already present at the time of informed consent should be recorded in Medical History CRF.

CRA is to review and confirm patient's eligibility for re-screening with the site.

Patients may be re-screened only once and must be randomized within 28 days after the recorded rescreening date. All evaluations including original assessments and repeated assessments must be collected on the eCRF.

7.1.2.2 Information to be collected on screening failures

Patients who sign an informed consent but fail to be started on treatment for any reason will be considered a screen failure. The reason for not being started on treatment will be entered on the Screening Phase Disposition Page. The demographic information, informed consent, and Inclusion/Exclusion pages must also be completed for Screen Failure Patients. No other data will be entered into the clinical database for patients who are screen failures, unless the patient experienced a Serious Adverse Event during the Screening Phase (see Section 8 for SAE reporting details). If the patient fails to be randomized, the IRT must be notified within 2 days of the screen fail that the patient was not randomized.

7.1.2.3 Patient demographics and other baseline characteristics

The following patient demographics and baseline characteristics will be collected on the eCRF:

- ER and HER2 status
- Demography including year of birth, race and ethnicity
- Height, weight (See Section 7.2.2.3)
- Diagnosis and extent of cancer (including staging, histology/cytology and sites of disease at study entry)
- Medical history (e.g., important medical, surgical, and allergic conditions from the patient's medical history which could have an impact on the patient's evaluation) / current medical conditions (e.g., all relevant current medical conditions which are present at the time of signing informed consent). Ongoing medical conditions, symptoms and disease which are recorded on the Medical History eCRF should include the toxicity grade.
- Prior and concomitant medications
- Prior anticancer therapy (surgery, medication, radiotherapy); specifically, for prior anticancer therapy the following data will be collected: start and end date of therapy, setting (neo-adjuvant, adjuvant, metastatic), best response and reason for discontinuation of therapy.
- Physical Examination (See Section 7.2.2.1)
- Vital signs including blood pressure and pulse (See Section 7.2.2.2)
- ECOG performance status (See Section 7.2.2.4)

[redacted]

- ECG (See Section 7.2.2.5.3)
- Tumor evaluation (See Section 7.2.1)
- Laboratory evaluations (e.g., hematology, coagulation, biochemistry, fasting serum lipid profile/ urinalysis) (See Section 7.2.2.5)
- Hepatitis B serologic markers and viral load: HBV-DNA HBsAg, HBcAb, and HBsAb.
- The patients listed in Section 6.3.2.3 should be tested for HCV RNA. If the patient is already known to have a chronic infection with HBV or HCV and is taking anti-HBV medication, the site does not have to wait for the screening HBV or HCV results prior to the first dose of the study treatment.
- Pulmonary function tests (PFTs) can be performed at screening or Treatment Day 1 (prior to administration of the study treatment) and during the trial if clinically indicated and if warranted to exclude or manage non-infection pneumonitis. Chest CT scans also be considered and can be used for exclude or manage non-infectious pneumonitis.

7.1.3 Treatment period

Patients will be randomized to one of the two treatment arms (everolimus plus exemestane arm or placebo plus exemestane arm) within 7 days before Cycle 1 Day 1. Randomization will be stratified according to visceral disease status and sensitivity to prior hormonal therapy status. Patients will receive treatment until disease progression, unacceptable toxicity, death, or discontinuation from the study treatment due to any other reason.

7.1.4 Discontinuation of study treatment

Patients may voluntarily discontinue from the study treatment for any reason at any time. If a patient decides to discontinue from the study treatment, the investigator should make a reasonable effort (e.g. telephone, e-mail, letter) to understand the primary reason for this decision and record this information in the patient's chart and on the appropriate CRF pages. They may be considered withdrawn if they state an intention to withdraw, fail to return for visits, or become lost to follow-up for any other reason.

The investigator should discontinue study treatment for a given patient, if he/she believes that continuation would be detrimental to the patient's well-being.

Study treatment must be discontinued under the following circumstances:

- Emergence of specific adverse events or laboratory abnormalities under some circumstances as outlined in Section 6.
- Use of prohibited treatment (refer to Section 6.4.1)
- Any protocol deviation that results in a significant risk to the patient's safety

The appropriate personnel from the site and Novartis will assess whether study treatment should be discontinued for any patient whose treatment code has been broken inadvertently for any reason.

Patients who discontinue study treatment should NOT be considered withdrawn from the study. They should return for the assessments indicated in Section 7.2.1. If they fail to return

[redacted]

for these assessments for unknown reasons, every effort (e.g. telephone, email, or letter) should be made to contact them as specified in Section 7.1.7.

Patients who discontinue study treatment should undergo an end of treatment visit.

For patients who discontinue treatment for reasons other than documented disease progression, death, lost to follow-up, or withdrawal of consent, tumor assessments must continue to be performed per assessment schedule until documented disease, death, lost to follow-up, or withdrawal of consent.

The investigator must also contact the IRT to register the patient's discontinuation from study treatment within 2 days.

7.1.4.1 Replacement policy

Not applicable.

7.1.5 Withdrawal of consent

Patients may voluntarily withdraw consent to participate in the study for any reason at any time. Withdrawal of consent occurs only when a patient does not want to participate in the study any longer, and does not want any further visits or assessments, and does not want any further study related contact.

Novartis will continue to retain and use all research results that have already been collected for the study evaluation. All biological samples that have already been collected may be retained and analyzed at a later date (or as required by local regulations).

If a patient withdraws consent, the investigator should make a reasonable effort (e.g. telephone, e-mail, letter) to understand the primary reason for this decision and record this information.

Study treatment must be discontinued and no further assessments conducted.

Further attempts to contact the patient are not allowed unless safety findings require communication or follow up.

7.1.6 Follow-up period

7.1.6.1 Follow up for safety evaluations

All patients must have safety evaluations for 30 days after the last dose of study treatment. Adverse events and SAE information will be collected and recorded in the appropriate eCRFs. Record all concomitant medications or therapies used/taken to treat the SAEs.

Antineoplastic therapy regimen received between EOT and progression will also be collected as well as the first regimen received after progression.

For patients who are lost to follow-up, the investigator should show "due diligence" by documenting in the source documents steps taken to contact the patient, e.g., dates of telephone calls, registered letters, etc. The investigator must determine the primary reason for a patient's premature withdrawal from the study and record this information on the appropriate disposition CRF.

[redacted]

7.1.6.2 Post-treatment follow-up

All patients who discontinued treatment during the treatment phase for reasons other than death, consent withdrawal, lost to follow-up or disease progression as per investigator assessment (Section 7.1.3) will continue tumor assessments as per Table 7-1 thereafter until PD as per investigator assessment, withdrawal of consent or death. Once the patient ceases tumor follow-up, the reason for completion should be recorded on the End of Post Treatment Disposition eCRF page.

7.1.6.3 Survival follow-up

All patients will be followed for survival status at least every 3 months after randomized treatment phase or post-treatment follow-up phase, unless they discontinued due to death, consent withdrawal or lost to follow-up. Survival information can be obtained via phone and information will be documented in the source documents and eCRF. Additional survival assessments may be performed more frequently if a survival update is required for reporting the results or to meet safety or regulatory requirements.

7.1.7 Lost to follow-up

For patients whose status is unclear because they fail to appear for study visits without stating an intention to withdraw consent, the investigator should show "due diligence" by contacting the patient, family or family physician as agreed in the informed consent and by documenting in the source documents steps taken to contact the patient, e.g. dates of telephone calls, registered letters, etc. A patient should not be considered lost to follow-up until due diligence has been completed. Patients lost to follow up should be recorded as such on the appropriate Disposition CRF.

7.2 Assessment types

7.2.1 Efficacy assessments

Efficacy assessments

Tumor response will be assessed locally and centrally according to the Novartis guideline version 3.2 (Appendix 1) based on RECIST 1.1 (Eisenhauer et al 2009). The imaging assessment collection plan is presented in Table 7-2. Details of the central review process will be described in the independent review charter.

Imaging data will be centrally collected and checked for quality by an imaging Contract Research Organization (CRO) designated by Novartis. Central review should stop after the number of events is reached for the primary PFS analysis. The results of the local investigator’s assessment will be used for primary analysis purposes.

[redacted]

Table 7-2 Imaging assessment collection plan

Procedure	Screening/Baseline	During Treatment/Follow-up
Chest, abdomen and pelvis CT or MRI(with intravenous contrast enhancement)	Mandated	Mandated, every 8 weeks after randomization date until PD(± 7 days); the assessment will be performed very 12 weeks after primary analysis data cut-off or as clinically indicated, until PD(± 7 days)
Brain CT or MRI	Mandated	Mandated if lesions were documented at baseline, follow same schedule as CT/MRI of chest, abdomen, and pelvis
Whole body bone scan	Mandated	If clinically indicated
Localized bone CT, MRI or x-ray	For any lesions identified on the whole body bone scan that are not visible on the chest, abdomen and pelvis CT or MRI	Mandated if lesions were documented at baseline, follow same schedule as CT/MRI of chest, abdomen, and pelvis
Color photography (with scale/ruler)	For any skin lesions present	Mandated if lesions were documented at baseline, follow same schedule as CT/MRI of chest, abdomen, and pelvis
CT or MRI of other metastatic sites (e.g., neck)	If clinically indicated	Mandated if lesions were documented at baseline, follow same schedule as CT/MRI of chest, abdomen, and pelvis

Baseline imaging assessments

Imaging assessments will be performed at screening/baseline within 21 days before start of treatment.

Any imaging assessments already completed during the regular work-up of the patient within 21 days prior to start of treatment, including before signing the main study ICF, can be considered as the baseline images for this study. Any imaging assessments obtained after randomization cannot be considered baseline images. The following assessments are required at screening/baseline:

- Chest, abdomen and pelvis CT or MRI
- Brain CT or MRI
- Whole body bone scan
- Localized bone CT, MRI or x-ray, for any lesions identified on the whole body bone scan that are not visible on the chest, abdomen and pelvis CT or MRI
- Color photography for any skin lesions present
- CT or MRI of other metastatic sites (e.g., neck), if clinically indicated

If a patient is known to have a contraindication to CT intravenous (IV) contrast media or develops a contraindication during the trial, a non-contrast CT of the chest (MRI is not recommended due to respiratory artifacts, however if CT is not feasible per local regulations, MRI can be performed instead) plus a contrast-enhanced MRI (if possible) of the abdomen and pelvis should be performed.

[redacted]

If brain metastases are suspected at baseline, brain MRI or CT should be completed. Contrast enhanced brain MRI is preferred, however, if MRI contrast is contraindicated, then MRI without contrast or CT with/without contrast is acceptable.

A whole body bone scan should be performed per institutional standard of care [e.g., Tc-99 bone scan, whole body bone MRI, Fluorodeoxyglucose positron emission tomography (FDG-PET) or sodium fluoride (NaF) PET]. After screening, whole body bone scans is not required, unless clinically indicated. If indicated, the same methodology as at screening should be used.

Localized CT, MRI or X-rays should be acquired for all skeletal lesions identified on the screening whole body bone scan, which are not visible on the chest, abdomen and pelvis CT/MRI. If skeletal lesions are documented at baseline, scans need to be continued following the same schedule as CT/MRI of chest, abdomen and pelvis. The same methodology as at screening should be used.

If clinically indicated, CT or MRI of other areas (e.g., neck) of disease as appropriate should be performed.

If skin lesions are present at screening, color photography should be acquired using a digital camera in clear focus, including a scale/ruler, in such a way that the size of the lesion(s) can be determined from the photograph.

Any potentially measurable lesion that has been previously treated with radiotherapy should be considered as a non-measurable lesion. However, if a lesion previously treated with radiotherapy has clearly progressed since the radiotherapy, it can be considered as a measurable lesion.

Chest x-rays and ultrasound should not be used to measure tumor lesions.

Post-baseline imaging assessments

Imaging assessments as described in Table 7-2 should be performed at the time-points specified using the same imaging modality used at baseline, irrespective of study treatment interruption or actual dosing (see Table 7-1). Imaging assessments for response evaluation will be performed every 8 weeks (± 7 days) after randomization. Tumor assessments are to be performed every 12 weeks after primary analysis data cut-off and as clinically indicated, until disease progression.

Additional imaging assessments may be performed at any time during the study at the investigator's discretion to support the efficacy evaluations for a subject, as necessary. Clinical suspicion of disease progression at any time requires a physical examination and imaging assessments to be performed promptly rather than waiting for the next scheduled imaging assessment.

Each lesion that is measured at baseline must be measured by the same method (either same imaging method or by photography, including a metric ruler) and when possible, the same local radiologist/physician throughout the study so that the comparison is consistent. If an off-schedule imaging assessment is performed because progression is suspected, subsequent imaging assessments should be performed in accordance with the original imaging schedule.

[redacted]

Combined PET/CT may be used only if the CT is of similar diagnostic quality as a CT performed without PET, including the utilization of IV contrast media. At the discretion of the Investigators, FDG-PET scans may be performed to document progressive disease per RECIST 1.1 (Appendix 1).

All study imaging (including any off-schedule imaging studies) should be submitted to the designated imaging CRO for quality control and central review.

7.2.2 Safety and tolerability assessments

Safety assessments will consist of monitoring and recording all adverse events (AEs), including serious adverse events (SAEs), the regular monitoring of hematology, serum chemistry, coagulation, routine monitoring of vital signs (heart rate, blood pressure, and body temperature), weight, ECOG performance status, CT scans of the chest, abdominal, and pelvic area, physical examinations, and pulmonary function tests (PFTs) if clinically indicated. For details on AE collection and reporting, refer to Section 8.

These assessments should be performed ± 7 days of the scheduled day of assessment (Table 7-1) except for adverse events and concomitant medications that will be evaluated and recorded continuously throughout the study.

Significant findings of any safety evaluation must be recorded either on the Medical History eCRF (if present before signing informed consent) or on the Adverse Events eCRF (if newly occurring or worsening since signing informed consent).

7.2.2.1 Physical examination

A complete physical examination must be performed at screening and later as clinically indicated and will include the examination of general appearance, skin, neck (including thyroid), eyes, ears, nose, throat, lungs, heart, abdomen, back, lymph nodes, extremities, vascular and neurological. If indicated based on medical history and/or symptoms, rectal, external genitalia, breast, and pelvic exams will be performed.

A short physical exam will include the examination of general appearance and vital signs (blood pressure [BP] and pulse).

Significant findings that were present prior to the signing of informed consent must be included in the Medical History page on the patient's CRF. Significant new findings that begin or worsen after informed consent must be recorded on the Adverse Event page of the patient's CRF.

7.2.2.2 Vital signs

Vital signs include blood pressure (supine position preferred when ECG is collected), pulse measurement, and body temperature.

7.2.2.3 Height and weight

Height will be measured at screening.

Body weight (in indoor clothing, but without shoes) will be measured at screening and at subsequent time points as specified in Table 7-1.

[redacted]

7.2.2.4 Performance status

Assessment of ECOG performance status will be performed as per the assessment schedule in Table 7-1,. More frequent examinations may be performed at the investigator's discretion, if medically indicated.

Table 7-3 ECOG Performance Status Scale

Score	Performance Status
0	Fully active, able to carry on all pre-disease performance without restriction.
1	Restricted in physically strenuous activity but ambulatory and able to carry out work of a light or sedentary nature, e.g., light housework, office work.
2	Ambulatory and capable of all self-care but unable to carry out any work activities. Up and about more than 50% of waking hours.
3	Capable of only limited self-care, confined to bed or chair more than 50% of waking hours.
4	Completely disabled. Cannot carry on any self-care. Totally confined to bed or chair
5	Dead

7.2.2.5 Laboratory evaluations

The standard clinical laboratory analyses described below are to be performed by the study site's local laboratories according to the Visit Schedule, outlined in Table 7-1. More frequent examinations may be performed at the investigator's discretion if medically indicated; results should be recorded in the eCRFs.

Novartis must be provided with a copy of the laboratory certification and a tabulation of the normal ranges for each parameter required. In addition, if at any time a patient has laboratory parameters obtained from a different outside laboratory, Novartis must be provided with a copy of the certification and a tabulation of the normal ranges for that laboratory.

Abnormal laboratory parameters which are clinically relevant (e.g., require dose modification and/or interruption of study treatment, lead to clinical symptoms or signs or require therapeutic intervention), whether specifically requested in the protocol or not, must be recorded in the eCRF. When abnormal laboratory values or test results constitute an adverse event (Section 8.1.1) it must be recorded on the eCRF Adverse Events page.

Hematology, biochemistry, lipid panel, and coagulation assessments performed ≤ 7 days of the first dose of study treatment do not need to be repeated on Day 1 of Cycle 1.

The frequency of the assessments is indicated in Table 7-1 and is to be repeated if clinically indicated.

[redacted]

Table 7-4 Local clinical laboratory parameters collection plan

Test Category	Test Name
Hematology	Hematocrit, Hemoglobin, Platelets, Red blood cells, White blood cells, Differential (Basophils, Eosinophils, Lymphocytes, Monocytes, Neutrophils)
Biochemistry	Albumin, Alkaline phosphatase, ALT, AST, Gamma-glutamyl-transferase (GGT), Lactate dehydrogenase (LDH), Calcium, Magnesium, Phosphorus, Sodium, Potassium, Total Protein, Creatinine, Creatine kinase, Direct Bilirubin, Total Bilirubin, LDL, HDL Blood Urea Nitrogen (BUN) or Urea, Uric Acid, Glucose (fasting)
Coagulation	International normalized ratio [INR], Activated partial thromboplastin time (APTT)
Serum lipid profile	Total cholesterol and triglycerides
Hepatitis markers	HBV-DNA, HbsAg, HbsAb, HbcAb, HCV RNA-PCR (baseline)

7.2.2.5.1 Hematology

Hematology tests include a complete blood count (CBC). A total white blood cell (WBC) with absolute differentials (including neutrophil count plus bands, lymphocyte, monocyte, eosinophil, basophil counts), hemoglobin (Hgb), and a platelet count. Hematologic tests will be performed at screening or on C1D1 (repeated prior to administration of the study treatment, if greater than 7 days since screening), and then every 2 cycles after C1D1.

7.2.2.5.2 Clinical chemistry/ serum lipid profile

Serum chemistry tests include Albumin, Alkaline phosphatase, ALT, AST, Gamma-glutamyl-transferase (GGT), Lactate dehydrogenase (LDH), Calcium, Magnesium, Phosphorus, Sodium, Potassium, Creatinine, Creatine kinase, Direct Bilirubin, Total Bilirubin, Total Protein, Total Cholesterol, LDL, HDL, Total Triglycerides, Blood Urea Nitrogen (BUN) or Urea, Uric Acid, Glucose(fasting state for at least 12 hours). Serum chemistries must be performed at screening or on C1D1 (repeated prior to administration of the study treatment, if greater than 7 days since screening), and then every 2 cycles after C1D1.

7.2.2.5.3 Electrocardiogram (ECG)

A standard 12 lead ECG will be performed

- At screening and/or baseline
- If clinically indicated

Each ECG tracing should be labeled with the study number, patient initials (where regulations permit), subjectnumber, date, and kept in the source documents at the study site. Clinically significant abnormalities present when the patient signed informed consent should be reported on the Medical History CRF page. Clinically significant findings must be discussed with Novartis prior to enrolling the patient in the study. New (including new or worsened) clinically significant findings occurring after informed consent must be recorded on the Adverse Events CRF page.

Baseline ECG is a standard 12 lead and must be performed within 14 days of first dose for patients enrolled and may be repeated at the investigator's discretion if clinically indicated. Standard triplicate 12 lead ECG recording will be performed after the patient has been resting for approximately 10 min prior to each ECG collection time point.

[redacted]

Additional, unscheduled, safety ECGs may be repeated at the discretion of the investigator at any time during the study as clinically indicated. Unscheduled ECGs with clinically significant findings should be collected in triplicate. Local cardiologist ECG assessment may also be performed at any time during the study at the discretion of the investigator.

7.2.3 Pharmacokinetics

Pharmacokinetic blood sample collection and handling are described in Section 7.2.3.1.

Blood samples for Estradiol (E2) levels will be obtained at screening (or at Day 1 prior to study treatment) and at Cycle 1 Weeks 4 or any later one of the scheduled visits. This will allow evaluation of changes in estradiol levels after 4 weeks of study treatment.

Pre-dose blood samples for everolimus and exemestane trough levels will be collected at steady state at Cycle 1 Week 4 or any one of later scheduled visits. Steady-state is defined as having no dose adjustment/interruption of everolimus and exemestane in the previous 4 days prior to the day of the pre-dose PK sample collection.

On the days of pre-dose PK sample collection, patients should come back to the clinic to get their medications and ingest the medications at the clinic immediately after collection of the pre-dose blood samples. These pre-dose blood samples will allow determination of Cmin for everolimus and exemestane.

Patients who have ingested their medications at home prior to the study visit or have dose adjustment/interruption in the previous 4 days should not have their PK blood samples collected at that study visit, and the collection of the PK blood samples will be postponed to the next scheduled visit.

The collection date and time of all pre-dose PK samples must be documented (both for everolimus and exemestane, separately) in the Pharmacokinetic Blood Collection eCRF pages. The exact date and time of the everolimus and exemestane doses administered on the previous day prior to the visit day for the pre-dose PK sample must be entered on the eCRF. In addition, the exact date and time of the everolimus and exemestane doses administered immediately after the pre-dose PK sample collection on the visit day must be entered on the eCRF.

If the patient vomits within the first 4 hours following study-drug administration on the day of or the day before pharmacokinetic blood sampling, the time (using the 24-hours clock) of vomiting should be recorded on the AE eCRFs, and the collection of the PK blood samples will be postponed to the next scheduled visit.

No additional trial medication should be taken that day in an effort to replace the study drug that has been vomited.

If everolimus administration has been interrupted due to study drug suspected toxicities, then the PK sample collection should be postponed to the next visit.

7.2.3.1 Pharmacokinetic blood sample collection and handling

Blood PK samples will be collected by either direct venipuncture or an indwelling cannula inserted in a forearm vein (2ml of venous blood for everolimus levels, 4 mL for exemestane

[redacted]

for determination in plasma, 5 ml for estradiol levels) according to the following schedules (Table 7-5) described above.

Everolimus

At Cycle 1 Week 4, any day during week 4, immediately before and 2 hours after administration of everolimus on that day, 2 mL of blood will be collected into an EDTA containing tube. Immediately after each tube of blood is drawn, it should be inverted gently several times to ensure the mixing of tube contents (e.g., anticoagulant). Prolonged contact must be avoided with rubber stopper. The whole blood sample will then be transferred to a labeled polypropylene screw cap tube and stored at -20°C or below within 60 minutes of the draw time.

Exemestane

At Cycle 1 Week 4, any day during week 4, immediately before and 2 hours after administration of exemestane, 4 ml of blood will be collected in heparinized tubes, chilled in an ice-water bath and centrifuged at 4°C (1200g, 10 min) within 30 min, to avoid possible degradation of parent drug. Plasma samples will be stored at -20° C or below until analysis and within 60 min of the draw time.

Estradiol (E_2)

At screening or at day 1 before study treatment and at Cycle 1 Week 4, any day during week 4, visit 4 (week 4)before administration of everolimus and exemestane, 5 ml of blood will be collected in heparinized vials. Plasma will be separated by centrifugation within 30 min and stored at - 20 ° C or below until analysis and within 60 min of the draw time.

[redacted]

Table 7-5 PK blood collection schedule

Analyte / Drug	Visit Name	Day	Timepoint	PK Sample number	Dose Reference ID	Blood Vol. (mL)
Estradiol (E_2)	Screening/baseline	-7 to 1	From -7 days before to day 1 at pre-dose (prior to any study drug)	1	1	5mL
Estradiol (E_2)	Cycle 1/week 4	Week 4	Pre-dose (prior to any study drug)	2	2/3*	5mL
Everolimus/ Placebo	Cycle 1/week 4	Week 4	Pre-dose (prior to everolimus administration)	101	101/102*	2ml
Everolimus/ Placebo	Cycle 1/week 4	Week 4	2 hours post everolimus administration	102	101	2ml
Exemestane	Cycle 1/week 4	Week 4	Pre-dose (prior to exemestane administration)	201	201/202*	4mL
Exemestane	Cycle 1/week 4	Week 4	2 hours post exemestane administration	202	201	4mL

- Exact date and time of last treatment administration (everolimus and exemestane separately) must be recorded on the eCRF. Also, exact dates and times of sample collection must be recorded on the Pharmacokinetic Blood Collection eCRF. Example: For everolimus pre-dose 2mL blood sample, the time and the date of the everolimus administration on the everolimus PK blood samples collection day should be recorded. In addition the time and date of the last everolimus administration on the previous day should also be recorded.
- If PK samples cannot be collected from a patient at the scheduled visit (Week 4) for reasons such as the sample would not be at steady-state due to dose interruption or vomiting of the prior dose, the patient should come back at the next scheduled visit to have the PK samples collected.
- The first dose reference ID refers to the first dose of everolimus received after collection of the pre-dose PK sample, while the second dose reference ID is the last dose the subject received prior to the collection of the pre-dose PK sample

Pharmacokinetic sample shipment

All samples must be labeled and frozen upon collection below -20°C and kept frozen thereafter. At the end of each treatment period, all samples must be carefully packed in suitable packing material containing sufficient dry ice to keep them frozen during shipment.

A list of samples, including the date, subject initials and number, and time of sampling has to be sent with the samples. Any missing samples should be flagged on the list.

Analysis of pharmacokinetic samples will be centralized. Details regarding addresses of shipments, carrier recommendations, labeling of the parcels, days of shipments, contact details and other samples logistics will be provided in a separated laboratory manual.

7.2.3.2 Analytical method

Everolimus concentrations in whole blood will be determined by a LC-MS/MS method. The lower limit of quantitation (LLOQ) will be 0.3 ng/mL.

[redacted]

Exemestane will be determined in plasma using a LC-MS/MS method. The method has a lower limit of quantification (LLOQ) of 20 pg/mL.

Estradiol will be determined in plasma using competitive immunoassay. The LLOQ of the assay is equivalent to 2.0 pmol/L estradiol in plasma samples.

7.2.4 Biomarkers

Not applicable.

7.2.5 Resource utilization

Not applicable.

7.2.6 Patient reported outcomes

Not applicable

8 Safety monitoring and reporting

8.1 Adverse events

8.1.1 Definitions and reporting

An adverse event is defined as the appearance of (or worsening of any pre-existing) undesirable sign(s), symptom(s), or medical condition(s) that occur after patient's signed informed consent has been obtained.

Abnormal laboratory values or test results occurring after informed consent constitute adverse events only if they induce clinical signs or symptoms, are considered clinically significant, require therapy (e.g., hematologic abnormality that requires transfusion or hematological stem cell support), or require changes in study medication(s).

Adverse events that begin or worsen after informed consent should be recorded in the Adverse Events eCRF. Conditions that were already present at the time of informed consent should be recorded in the Medical History CRF page of the patient's CRF. Adverse event monitoring should be continued for at least 30 days following the last dose of study treatment. Adverse events (including lab abnormalities that constitute AEs) should be described using a diagnosis whenever possible, rather than individual underlying signs and symptoms. When a clear diagnosis cannot be identified, each sign or symptom should be reported as a separate Adverse Event.

The adverse events of special interest (AESIs) include the following category

- Stomatitis/
- Pre-existing infection (reactivation, aggravation, or exacerbation)
- Cardiac failure

Adverse events will be assessed according to the Common Terminology Criteria for Adverse Events (CTCAE) version 4.03.

[redacted]

If CTCAE grading is not available for an adverse event, the severity of mild, moderate, severe, and life-threatening, death related to the AE corresponding to Grades 1 - 5, respectively, will be used. Information about any deaths (related to an Adverse Event or not) will also be collected though a Death form.

The occurrence of adverse events should be sought by non-directive questioning of the patient (subject) during the screening process after signing informed consent and at each visit during the study. Adverse events also may be detected when they are volunteered by the patient (subject) during the screening process or between visits, or through physical examination, laboratory test, or other assessments. As far as possible, each adverse event should be evaluated to determine:

1. The severity grade (CTCAE Grade 1-5)
2. Its duration (Start and end dates)
3. Its relationship to the study treatment (Reasonable possibility that AE is related: [No; Yes, Yes, investigational treatment; Yes, other study treatment (non-investigational); Yes, both and/or indistinguishable]
4. Action taken with respect to study or investigational treatment (dose increased, dose not changed, dose reduced, drug interrupted, drug withdraw, unknown, not applicable
5. Whether medication or therapy was given (no concomitant medication/non-drug therapy, concomitant medication/non-drug therapy)
6. Whether it is serious, where a serious adverse event (SAE) is defined as in Section 8.2.1 and which seriousness criteria have been met
7. Outcome (not recovered/not resolved, recovered/resolved, recovering/resolving, recovered/resolved with sequalae, fatal, unknown)

If the event worsens the event should be reported a second time in the CRF noting the start date when the event worsens in toxicity. For grade 3 and 4 adverse events only, if improvement to a lower grade is determined a new entry for this event should be reported in the CRF noting the start date when the event improved from having been Grade 3 or Grade 4.

All adverse events should be treated appropriately. If a concomitant medication or non-drug therapy is given, this action should be recorded on the Adverse Event CRF.

Once an adverse event is detected, it should be followed until its resolution or until it is judged to be permanent, and assessment should be made at each visit (or more frequently, if necessary) of any changes in severity, the suspected relationship to the study treatment, the interventions required to treat it, and the outcome.

8.1.2 Laboratory test abnormalities

8.1.2.1 Definitions and reporting

Laboratory abnormalities that constitute an Adverse event in their own right (are considered clinically significant, induce clinical signs or symptoms, require concomitant therapy or require changes in study treatment), should be recorded on the Adverse Events CRF. Whenever possible, a diagnosis, rather than a symptom should be provided (e.g. anemia instead of low hemoglobin). Laboratory abnormalities that meet the criteria for Adverse Events should be followed until they have returned to normal or an adequate explanation of

[redacted]

the abnormality is found. When an abnormal laboratory or test result corresponds to a sign/symptom of an already reported adverse event, it is not necessary to separately record the lab/test result as an additional event.

Laboratory abnormalities, that do not meet the definition of an adverse event, should not be reported as adverse events. A Grade 3 or 4 events (severe) as per CTCAE does not automatically indicate a SAE unless it meets the definition of serious as defined below and/or as per investigator's discretion. A dose hold or medication for the lab abnormality may be required by the protocol in which case the lab abnormality would still, by definition, be an adverse event and must be reported as such.

8.2 Serious adverse events

8.2.1 Definitions

Serious adverse event (SAE) is defined as one of the following:

- Is fatal or life-threatening
- Results in persistent or significant disability/incapacity
- Constitutes a congenital anomaly/birth defect
- Is medically significant, i.e., defined as an event that jeopardizes the patient or may require medical or surgical intervention to prevent one of the outcomes listed above
- Requires inpatient hospitalization or prolongation of existing hospitalization,
- Note that hospitalizations for the following reasons should not be reported as serious adverse events:
  - Routine treatment or monitoring of the studied indication, not associated with any deterioration in condition
  - Elective or pre-planned treatment for a pre-existing condition that is unrelated to the indication under study and has not worsened since signing the informed consent
  - Social reasons and respite care in the absence of any deterioration in the patient's general condition
- Note that treatment on an emergency outpatient basis that does not result in hospital admission and involves an event not fulfilling any of the definitions of a SAE given above is not a serious adverse event

8.2.2 Reporting

To ensure patient safety, every SAE, regardless of suspected causality, occurring after the patient has provided informed consent and until at least 30 days after the patient has stopped study treatment must be reported to Novartis within 24 hours of learning of its occurrence.

Any additional information for the SAE including complications, progression of the initial SAE, and recurrent episodes must be reported as follow-up to the original episode within 24 hours of the investigator receiving the follow-up information. An SAE occurring at a different time interval or otherwise considered completely unrelated to a previously reported one should be reported separately as a new event.

[redacted]

Any SAEs experienced after the 30 day safety evaluation follow-up period should only be reported to Novartis if the investigator suspects a causal relationship to the study treatment.

Information about all SAEs is collected and recorded on the Serious Adverse Event Report Form; all applicable sections of the form must be completed in order to provide a clinically thorough report. The investigator must assess and record the relationship of each SAE to each specific study treatment (if there is more than one study treatment), complete the SAE Report Form in English, and submit the completed form within 24 hours to Novartis. Detailed instructions regarding the SAE submission process and requirements for signatures are to be found in the investigator folder provided to each site.

Follow-up information is submitted in the same way as the original SAE Report. Each re-occurrence, complication, or progression of the original event should be reported as a follow-up to that event regardless of when it occurs. The follow-up information should describe whether the event has resolved or continues, if and how it was treated, whether the blind was broken or not, and whether the patient continued or withdrew from study participation.

If the SAE is not previously documented in the Investigator's Brochure or Package Insert (new occurrence) and is thought to be related to the Novartis study treatment, an oncology Chief Medical Office and Patient Safety (CMO&PS) department associate may urgently require further information from the investigator for Health Authority reporting. Novartis may need to issue an Investigator Notification (IN), to inform all investigators involved in any study with the same drug that this SAE has been reported. Suspected Unexpected Serious Adverse Reactions (SUSARs) will be collected and reported to the competent authorities and relevant ethics committees in accordance with Directive 2001/20/EC or as per national regulatory requirements in participating countries.

8.3 Emergency unblinding of treatment assignment

Emergency unblinding should only be undertaken when it is essential for effective treatment of the patient. Most often, study treatment discontinuation and knowledge of the possible treatment assignments are sufficient to treat a study patient who presents with an emergency condition. Emergency code breaks are performed using the IRT. When the investigator contacts the IRT to unblind a patient, he/she must provide the requested patient identifying information and confirm the necessity to unblind the patient. The investigator will then receive details of the drug treatment for the specified patient and a fax confirming this information. The system will automatically inform the Novartis monitor for the site and the Study Lead that the code has been broken.

It is the investigator's responsibility to ensure that there is a procedure in place to allow access to the IRT in case of emergency. The investigator will inform the patient how to contact his/her backup in cases of emergency when he/she is unavailable. The protocol number, study treatment name if available, patient's numbers, and instructions for contacting the local Novartis CPO (or any entity to which it has delegated responsibility for emergency code breaks) will be provided to the patient in case emergency unblinding is required at a time when the investigator and backup are unavailable. However, if a mechanism is already in place to ensure that the investigator and/or back-up can always be reached in case of emergency then the procedure above is not required.

[redacted]

Study treatment must be discontinued once emergency unblinding has occurred.

8.4 Pregnancies

Not applicable.

8.5 Warnings and precautions

No evidence available at the time of the approval of this study protocol indicated that special warnings or precautions were appropriate, other than those noted in the provided Investigator Brochure. Additional safety information collected between IB updates will be communicated in the form of Investigator Notifications. This information will be included in the patient informed consent and should be discussed with the patient during the study as needed.

8.6 Data Monitoring Committee

Not Applicable

8.7 Steering Committee

A steering committee (SC) will be established comprising investigators participating in the trial, i.e. not being members of the Novartis representatives from the Clinical Trial Team.

The SC will ensure transparent management of the study according to the protocol through recommending and approving modifications as circumstances require. The SC will review protocol amendments as appropriate. The SC will review blinded pooled safety data approximately every 6 months (after the first randomized patient has started study treatment) and provide recommendations to the sponsor regarding the conduct of the study. Together with the clinical trial team, the SC will also develop recommendations for publications of study results including authorship rules. The details of the role of the Steering Committee will be defined in a Steering Committee charter.

9 Data collection and management

9.1 Data confidentiality

Information about study subjects will be kept confidential and managed under the applicable laws and regulations. Those regulations require a signed subject authorization informing the subject of the following:

- What protected health information (PHI) will be collected from subjects in this study
- Who will have access to that information and why
- Who will use or disclose that information
- The rights of a research subject to revoke their authorization for use of their PHI.

In the event that a subject revokes authorization to collect or use PHI, the investigator, by regulation, retains the ability to use all information collected prior to the revocation of subject authorization. For subjects that have revoked authorization to collect or use PHI, attempts

[redacted]

should be made to obtain permission to collect follow-up safety information (e.g. has the subject experienced any new or worsened AEs) at the end of their scheduled study period.

The data collection system for this study uses built-in security features to encrypt all data for transmission in both directions, preventing unauthorized access to confidential participant information. Access to the system will be controlled by a sequence of individually assigned user identification codes and passwords, made available only to authorized personnel who have completed prerequisite training.

Prior to entering key sensitive personally identifiable information (Subject Initials and exact Date of Birth), the system will prompt site to verify that this data is allowed to be collected. If the site indicates that country rules or ethics committee standards do not permit collection of these items, the system will not solicit Subject Initials. Year of birth will be solicited (in the place of exact date of birth) to establish that the subject satisfies protocol age requirements and to enable appropriate age-related normal ranges to be used in assessing laboratory test results.

9.2 Site monitoring

Before study initiation, at a site initiation visit or at an investigator's meeting, Novartis personnel (or designated CRO) will review the protocol and CRFs with the investigators and their staff. During the study, the field monitor will visit the site regularly to check the completeness of patient records, the accuracy of entries on the CRFs, the adherence to the protocol to Good Clinical Practice, the progress of enrollment, and to ensure that study treatment is being stored, dispensed, and accounted for according to specifications. Key study personnel must be available to assist the field monitor during these visits.

The investigator must maintain source documents for each patient in the study, consisting of case and visit notes (hospital or clinic medical records) containing demographic and medical information, laboratory data, electrocardiograms, and the results of any other tests or assessments. All information recorded on CRFs must be traceable to source documents in the patient's file. The investigator must also keep the original signed informed consent form (a signed copy is given to the patient).

The investigator must give the monitor access to all relevant source documents to confirm their consistency with the CRF entries. Novartis monitoring standards require full verification for the presence of informed consent, adherence to the inclusion/exclusion criteria and documentation of SAEs. Additional checks of the consistency of the source data with the CRFs are performed according to the study-specific monitoring plan.

9.3 Data collection

The designated investigator staff will enter the data required by the protocol into the Electronic Case Report Forms (eCRF). The eCRFs have been built using fully validated secure web-enabled software that conforms to 21 CFR Part 11 requirements, Investigator site staff will not be given access to the EDC system until they have been trained. Automatic validation programs check for data discrepancies in the eCRFs and, allow modification or verification of the entered data by the investigator staff.

[redacted]

The Principal Investigator is responsible for assuring that the data entered into eCRF is complete, accurate, and that entry and updates are performed in a timely manner.

Radiological data will be acquired by the sites and interpreted locally. Additionally, radiological data will be transmitted by the sites to the respective CRO designated to undergo quality checks and central review. Details regarding all CRO procedures including collection and shipment of data will be described in the manual provided by the respective CRO.

In addition, data entered into IRT for screening, randomization, discontinuation, subsequent drug assignment and patient identifiers (i.e. date of birth, gender, and patient ID) will be transferred electronically to Novartis as described in the Data Transfer Specification for designated IRT vendor.

9.4 Database management and quality control

Novartis personnel (or designated CRO) will review the data entered by investigational staff for completeness and accuracy. Electronic data queries stating the nature of the problem and requesting clarification will be created for discrepancies and missing values and sent to the investigational site via the EDC system. Designated investigator site staffs are required to respond promptly to queries and to make any necessary changes to the data.

Concomitant treatments and prior medications entered into the database will be coded using the WHO Drug Reference List, which employs the Anatomical Therapeutic Chemical classification system. Medical history/current medical conditions and adverse events will be coded using the Medical dictionary for regulatory activities (MedDRA) terminology.

Data will be processed centrally and the results will be sent electronically to Novartis (or a designated CRO). Data that will be processed centrally includes:

- IRT data including information regarding screening, randomization, subsequent drug assignments and discontinuation
- Central imaging review data (i.e., CT/MRI, Bone scans and photography results)

Randomization codes and data about all study treatments dispensed to the patient and all IRT assigned dosage changes will be tracked using an Interactive Response Technology. The system will be supplied by a vendor(s), who will manage IRT and its database. The data will be sent electronically to Novartis personnel (or designated CRO).

At the conclusion of the study, the occurrence of any emergency code breaks will be determined after return of all code break reports and unused drug supplies to Novartis personnel (or designated CRO). The occurrence of any protocol violations will be determined. After these actions have been completed and the data has been verified to be complete and accurate, the database will be declared locked and the treatment codes will be unblinded and made available for data analysis.

10 Statistical methods and data analysis

It is planned that the data from all centers participating in the trial will be combined, so that an adequate number of patients are available for analysis. Novartis and/or a designated CRO will

[redacted]

perform all the analyses. Any additional data analyses performed independently by any investigator should be submitted to Novartis before publication or presentation.

The primary analysis for safety and efficacy will be conducted when approximately 110 PFS events have been documented.

The additional data for any patients continuing to receive study treatment, as allowed by the protocol, will be further summarized in a report once these patients completed the study (addendum of the clinical study report).

10.1 Analysis sets

10.1.1 Full Analysis Set

The Full Analysis Set (FAS) comprises all patients to whom study treatment has been assigned by randomization. According to the intent to treat principle, patients will be analyzed according to the treatment and strata they have been assigned to during the randomization procedure.

10.1.2 Safety set

The Safety Set consists of all patients who received at least one dose of study treatment. Patients will be analyzed according to treatment actually received, where treatment received is defined as the randomized treatment if the patient took at least one dose of that treatment or the first treatment received if the randomized treatment was never received.

10.1.3 Pharmacokinetic set (PAS)

The PAS consists of all patients with at least one available concentration

10.2 Patient demographics/other baseline characteristics

Baseline demographic and disease characteristics data will be listed and summarized descriptively by treatment group using the FAS.

Categorical data will be presented as frequencies and percentages. For continuous data, mean, standard deviation, median, minimum, and maximum will be presented. Medical history and current medical at baseline will be summarized separately by system organ class and preferred term, by treatment group.

10.3 Treatments (study treatment, concomitant therapies, compliance)

The safety set will be used for the analyses below.

Duration of everolimus, exemestane and placebo treatment, as well as dose intensity (computed as the ratio of actual dose received to actual duration) and relative dose intensity (computed as the ratio of the dose intensity to planned dose received/planned duration) will be listed and summarized using descriptive statistics, by treatment within each treatment arm.

The number of patients with dose changes/interruptions and the reasons will be presented by treatment for each treatment arm; all dosing data will be listed.

[redacted]

Concomitant medications and significant non-drug therapies prior to and after the start of the study treatment will be listed and summarized according to the Anatomical Therapeutic Chemical (ATC) classification system, by treatment arm.

These summaries will include medications starting on or after the start of study treatment or medications starting prior to the start of study treatment and continuing after the start of study treatment. Any prior concomitant medications or significant non-drug therapies starting and ending prior to the start of study treatment will be listed.

10.4 Primary objective

The primary objective in this study is to assess PFS on the combination treatment of everolimus + exemestane and placebo + exemestane in Chinese postmenopausal women with estrogen receptor positive, HER2-negative, locally advanced, recurrent, or metastatic breast cancer after recurrence or progression on prior letrozole or anastrozole.

10.4.1 Variable

The primary endpoint of this study is PFS, defined as the time from the date of randomization to the date of first documented progression or death due to any cause. A patient who has not progressed or died at the date of the analysis cut-off or receives another anticancer therapy will have their PFS censored at the time of the last adequate tumor evaluation before the earlier of the cut-off date or the anticancer therapy date. Disease progression for primary efficacy endpoint derivation will be assessed using the local (treating center's radiologist's) investigator's tumor assessment.

10.4.2 Statistical hypothesis, model, and method of analysis

No hypothesis testing will be performed and the objective of this study is to estimate hazard ratio of everolimus in combination with exemestane compared to exemestane alone.

PFS as determined by local evaluation will be analyzed based on the data from FAS according to the treatment arm and randomization strata to which patients were assigned. The distribution of PFS will be estimated using the Kaplan-Meier method. The median PFS along with 90% CI will be presented by treatment arm. The Kaplan-Meier curves will be displayed and the number of patients at risk at certain time points will be shown on the plot.

A Cox regression model stratified by randomization stratification factors will be used to estimate the hazard ratio (HR) of PFS, along with 90% CI. As this is an estimation based approach, no p-value will be provided.

10.4.3 Handling of missing values/censoring/discontinuations

PFS will be censored if no PFS event is observed before the cut-off date or before the start date of a new anticancer therapy or another investigational treatment, whichever occurs earlier. The censoring date will be the date of last adequate tumor assessment before either of these two dates. If a PFS event is documented after two or more missing assessments or non-adequate tumor assessments, then the date of PFS will be censored at the date of the last adequate tumor assessment. If a PFS event is observed after a single missing or non-adequate tumor assessment, the actual date of event will be used.

[redacted]

10.4.4 Supportive and sensitivity analyses

An un-stratified Cox model may be used to estimate the HR (together with associated 90% confidence interval) as supportive analysis, if appropriate. The primary analysis for PFS will be repeated with data based on central review for assessments of PFS and using the same analytical conventions as the primary analysis but as a secondary supportive analysis. Additional sensitivity analyses might be further specified in statistical analysis plan.

10.5 Secondary objectives

10.5.1 Secondary efficacy objectives

The secondary efficacy objectives in this study are to assess PFS in the two treatment arms as determined by a Blinded Independent Review Committee (BIRC) (Central review should be terminated after the number of events is reached for the primary PFS analysis), to assess the two treatment groups with respect to OS, ORR, deterioration in the ECOG PS, and CBR. Additionally, time to response and duration of response will be summarized between the treatment groups. Additional secondary objectives are to assess the two treatment groups with respect to safety and tolerability.

10.5.1.1 Overall Response Rate (ORR)

ORR is defined as the proportion of patients with best overall response of CR or PR according to RECIST 1. ORR will be calculated based on the FAS according to the ITT principle, using local radiologist's/investigator's tumor assessment. ORR will be estimated and the exact binomial 90% CI will be reported by treatment arm (Clopper and Pearson 1934). The above analyses will also be repeated using data from the independent central review as secondary supportive analysis.

10.5.1.2 Overall survival

Overall survival is defined as the time from date of randomization to date of death due to any cause. If the patient is alive at the date of the analysis cut-off or lost to follow-up, then OS will be censored at the last contact date prior to data cutoff date. The analyses for OS will be based on the data from FAS on an ITT basis, according to the treatment group patients are randomized to at baseline. The distribution function of OS will be estimated using the Kaplan-Meier method. The median OS along with 90% confidence intervals will be presented by two treatment groups, along with proportion of patients alive at 3, 6, 9, 12 and 18 months and the associated 90% confidence intervals. The stratified Cox regression will be used to estimate the HR of OS, along with 90% confidence interval.

10.5.1.3 Clinical benefit rate

CBR is defined as the proportion of patients with best overall response of CR, PR or SD with duration of 24 weeks or longer. CBR will be calculated based on the FAS according to the ITT principle, using local radiologist's/investigator's tumor assessment. CBR will be summarized for the two treatment groups using descriptive statistics along with exact 90% confidence intervals. The above analyses will also be repeated using data from the independent central review as secondary supportive analyses.

[redacted]

10.5.1.4 Time to response

Time to overall response (TTR) of CR or PR is defined as the time from randomization to first documented response (CR or PR, which must be confirmed subsequently) for patients with a confirmed CR or PR. TTR will be summarized in 3-month intervals using descriptive statistics. The analysis will be performed based on investigator assessment and also repeated using data from the independent central review as supportive analyses.

10.5.1.5 Duration of response

Among patients with a confirmed response (PR or CR) per RECIST 1.1 by investigator, DOR is defined as the time from first documented response (PR or CR) to the date of first documented disease progression or death due to any cause. DOR will be summarized using descriptive statistics. The analysis will be performed based on investigator assessment and also repeated using data from the independent central review as supportive analyses.

10.5.1.6 ECOG Performance Status

ECOG PS scale as described in Section 7.2.2.4 will be used to assess physical health of patients. An analysis of the time to definitive deterioration of the ECOG PS by one category of the score from baseline will be performed. Deterioration is considered definitive if no improvements in the ECOG PS status is observed at a subsequent time of measurement during the treatment period following the time point where the deterioration is observed. Death will be considered as worsening of the ECOG PS if it occurs close to the last available assessment, where "close" is defined as twice the planned period between two assessments. Patients who die after more than twice the planned period between two assessments are censored at the date of their last assessment before the cut-off.

Patients receiving any further anticancer therapy prior to definitive worsening will be censored at their date of last assessment prior to start of therapy. Patients that have not worsened at the data cut-off point will be censored at the date of last assessment prior to cut off. Kaplan-Meier method will be used to estimate the distribution function of time to definitive worsening. The estimated treatment-specific median times to definitive worsening will be presented along with a 90% confidence interval for the two treatment arms.

10.5.2 Safety objectives

10.5.2.1 Analysis set and grouping for the analyses

For all safety analyses, the safety set will be used.

The overall observation period will be divided into three mutually exclusive segments:

1. pre-treatment period: from day of patient's informed consent to the day before first dose of study treatment
2. on-treatment period: from day of first dose of study treatment to 30 days after last dose of study treatment
3. post-treatment period: starting at day 31 after last dose of study treatment.

[redacted]

The assessment of safety will be based mainly on the frequency of adverse events and on the number of laboratory values that fall outside of pre-determined ranges. Other safety data (e.g., electrocardiogram, vital signs) will be considered as appropriate.

10.5.2.2 Adverse events (AEs)

The safety summary tables will include only assessments from the on-treatment period, the treatment-emergent AEs. However, all safety data (including those from the pre and post-treatment periods) will be listed and those collected during the pre-treatment and post-treatment period are to be flagged.

All safety data collected in the study will be listed regardless of the study period.

The incidence of treatment-emergent adverse events (new or worsening from baseline) will be summarized by system organ class and/or preferred term, severity (based on CTC grades), type of adverse event and relation to study treatment by treatment group. Serious adverse events and non-serious adverse events during the on-treatment period will be tabulated.

The adverse events of special interest (AESIs) include the following category

- Stomatitis
- Pre-existing infection (reactivation, aggravation, or exacerbation)
- Cardiac failure

The number and percentage of patients with at least one AESI will be summarized by treatment arm.

All AEs, deaths and serious adverse events will be listed; assessments collected outside of the on-treatment period will be flagged.

10.5.2.3 Laboratory abnormalities

Grading of laboratory values will be assigned programmatically as per NCI Common Terminology Criteria for Adverse Events (CTCAE) version 4.03. The calculation of CTCAE grades will be based on the observed laboratory values only, clinical assessments will not be taken into account.

CTCAE Grade 0 will be assigned for all non-missing values not graded as 1 or higher. Grade 5 will not be used.

For laboratory tests where grades are not defined by CTCAE v4.03, results will be categorized as low/normal/high based on laboratory normal ranges.

The following summaries will be generated separately for haematology, and biochemistry tests:

- Listing of all laboratory data with values flagged to show the corresponding CTCAE v4.03 grades if applicable and the classifications relative to the laboratory normal ranges
- Listing of notable laboratory abnormalities (i.e. newly occurring CTCAE grade 3 or 4 laboratory toxicities)

For laboratory tests where grades are defined by CTCAE v4.03

[redacted]

- Worst post-baseline CTCAE grade (regardless of the baseline status). Each patient will be counted only once for the worst grade observed post-baseline.
- Shift tables using CTCAE grades to compare baseline to the worst on-treatment value

For laboratory tests where grades are not defined by CTCAE v4.03,

- Shift tables using the low/normal/high/ (low and high) classification to compare baseline to the worst on-treatment value.

10.5.2.4 Other safety data

Data from other tests (e.g. vital signs) will be listed, notable values will be flagged, and any other information collected will be listed as appropriate.

10.5.2.5 Supportive analyses for secondary objectives

Not applicable

10.5.2.6 Tolerability

Tolerability of study treatment will be assessed by summarizing the number of dose interruptions and dose reductions. Reasons for dose interruption and dose reductions, cumulative dose and dose intensity will be listed by patient.

10.5.3 Pharmacokinetics

Concentrations will be expressed in mass per volume units.

Valid C_{min} is defined as a pre-dose sample collected at steady-state, at 24 +/- 4 hours after the last dose, and collected prior to the next dose.

Steady-state condition for C_{min} is defined as no dose interruption/adjustment in the last 4 days prior to the pre-dose sample, no vomiting within 4 hours following study drug administration on the day of or the day before pharmacokinetic blood sampling.

Valid C_{2h} is defined as a 2 hour post-dose sample collected at steady-state.

Steady-state condition for C_{2h} is defined as no dose interruption/adjustment in the last 4 days prior to the C_{2h} sample, no vomiting within 4 hours following study drug administration on the day of or the day before pharmacokinetic blood sampling.

C_{min} and C_{2h} of everolimus will be both summarized by treatment group, and by actual lead dose, for patients with valid PK samples.

To assess the effect of everolimus on exemestane exposure, C_{min} and C_{2h} of exemestane with and without co-administration of everolimus will be compared separately after log-transformation using an ANOVA model with treatment as a fixed effect. C_{min} and C_{2h} ratios with everolimus to those without everolimus and their 90% CI will be calculated from the fitted models.

To assess the effect of everolimus on estradiol (E_2) level, changes in estradiol levels from baseline to week 4 will be summarized for the patients with valid PK samples. The relationship between everolimus C_{min} and change from baseline in estradiol at Week 4 will

[redacted]

also be analyzed by a model-based approach. This analysis will be repeated adjusted by the concomitant use of moderate/strong CYP3A4/PgP inducer/inhibitor via an appropriate covariate in the model.

C_{min} and C_{2h} will be analyzed based on with or without concomitant use of CYP3A4/PgP moderate/strong inhibitor or inducer to assess their effect on exposure of everolimus

Population PK models will be used to identify and quantify potential covariates that influence everolimus PK. Details of this analysis will be covered in a separate document.

A model-based approach will be used to explore:

- the potential relationships between efficacy (measured by appropriate efficacy endpoints such as PFS) and everolimus exposure based on C_{min}
- the potential relationship between appropriate safety endpoints and everolimus exposure based on C_{min} or C_{2h}
- the potential relationship between efficacy (measured by appropriate efficacy endpoints such as PFS) and changes in Estradiol (E_2) assuming a DDI of everolimus with exemestane

10.6 Sample size calculation

As this is a bridging study for China registration, this study will pursue an estimation strategy rather than formal hypothesis testing.

Based on the results of RAD001Y2301 study, the median PFS for the exemestane arm was 4.1 months, and the everolimus plus exemestane arm improved median PFS in Asian patients by 38%. Assuming the same hazard ratio of 0.62 in Chinese patients, this will result in an increase in median PFS from 4.1 months to 6.6 months for the everolimus plus exemestane arm, under the exponential model.

If the observed hazard ratio is 0.62, the 90% CI of the hazard ratio based on a total of 110 PFS events will be (0.45, 0.85). Assuming enrolment will continue for approximately 18 months at a uniform rate, and about 10% of the patients will be lost to follow-up for PFS evaluation, approximately 160 patients will need to be randomized to the two treatment groups in 1:1 ratio to observe the 110 PFS events at approximately 22 months following the randomization of the first patient in this study.

Below table shows the 90% CIs for various observed hazard ratios.

Table 10-1 90% CIs for various observed hazard ratios

placebo+exemestane (mPFS)	everolimus+exemestane (mPFS)	Hazard ratio	90% CI	
4.1	9.1	0.45	0.33	0.62
4.1	6.6	0.62	0.45	0.85
4.1	4.9	0.84	0.61	1.15

10.7 Power for analysis of key secondary variables

Not applicable.

[redacted]

11 Ethical considerations and administrative procedures

11.1 Regulatory and ethical compliance

This clinical study was designed, shall be implemented and reported in accordance with the ICH Harmonized Tripartite Guidelines for Good Clinical Practice, with applicable local regulations (including European Directive 2001/20/EC and US Code of Federal Regulations Title 21), and with the ethical principles laid down in the Declaration of Helsinki.

11.2 Responsibilities of the investigator and IRB/IEC/REB

The protocol and the proposed informed consent form must be reviewed and approved by a properly constituted Institutional Review Board/Independent Ethics Committee/Research Ethics Board (IRB/IEC/REB) before study start. Prior to study start, the investigator is required to sign a protocol signature page confirming his/her agreement to conduct the study in accordance with these documents and all of the instructions and procedures found in this protocol and to give access to all relevant data and records to Novartis monitors, auditors, Novartis Clinical Quality Assurance representatives, designated agents of Novartis, IRBs/IECs/REBs and regulatory authorities as required.

11.3 Informed consent procedures

Eligible patients may only be included in the study after providing written (witnessed, where required by law or regulation), IRB/IEC/REB-approved informed consent

Informed consent must be obtained before conducting any study-specific procedures (i.e. all of the procedures described in the protocol). The process of obtaining informed consent should be documented in the patient source documents. The date when a subject's Informed Consent was actually obtained will be captured in their CRFs.

Novartis will provide to investigators, in a separate document, a proposed informed consent form (ICF) that is considered appropriate for this study and complies with the ICH GCP guideline and regulatory requirements. Any changes to this ICF suggested by the investigator must be agreed to by Novartis before submission to the IRB/IEC/REB, and a copy of the approved version must be provided to the Novartis monitor after IRB/IEC/REB approval.

Additional consent form

Not applicable.

11.4 Discontinuation of the study

Novartis reserves the right to discontinue this study under the conditions specified in the clinical study agreement. Specific conditions for terminating the study are outlined in Section 4.4.

11.5 Publication of study protocol and results

Novartis assures that the key design elements of this protocol will be posted in a publicly accessible database such as clinicaltrials.gov. In addition, upon study completion and

[redacted]

finalization of the study report the results of this study will be either submitted for publication and/or posted in a publicly accessible database of clinical study results.

11.6 Study documentation, record keeping and retention of documents

Each participating site will maintain appropriate medical and research records for this trial, in compliance with Section 4.9 of the ICH E6 GCP, and regulatory and institutional requirements for the protection of confidentiality of subjects. As part of participating in a Novartis-sponsored study, each site will permit authorized representatives of the sponsor(s) and regulatory agencies to examine (and when required by applicable law, to copy) clinical records for the purposes of quality assurance reviews, audits and evaluation of the study safety and progress.

Source data are all information, original records of clinical findings, observations, or other activities in a clinical trial necessary for the reconstruction and evaluation of the trial. Examples of these original documents and data records include, but are not limited to, hospital records, clinical and office charts, laboratory notes, memoranda, subjects' diaries or evaluation checklists, pharmacy dispensing records, recorded data from automated instruments, copies or transcriptions certified after verification as being accurate and complete, microfiches, photographic negatives, microfilm or magnetic media, x-rays, and subject files and records kept at the pharmacy, at the laboratories, and medico-technical departments involved in the clinical trial.

Data collection is the responsibility of the clinical trial staff at the site under the supervision of the site Principal Investigator. The study case report form (CRF) is the primary data collection instrument for the study. The investigator should ensure the accuracy, completeness, legibility, and timeliness of the data reported in the CRFs and all other required reports. Data reported on the CRF, that are derived from source documents, should be consistent with the source documents or the discrepancies should be explained. All data requested on the CRF must be recorded. Any missing data must be explained. Any change or correction to a paper CRF should be dated, initialed, and explained (if necessary) and should not obscure the original entry. For electronic CRFs an audit trail will be maintained by the system. The investigator should retain records of the changes and corrections to paper CRFs.

The investigator/institution should maintain the trial documents as specified in Essential Documents for the Conduct of a Clinical Trial (ICH E6 Section 8) and as required by applicable regulations and/or guidelines. The investigator/institution should take measures to prevent accidental or premature destruction of these documents.

Essential documents (written and electronic) should be retained for a period of not less than fifteen (15) years from the completion of the Clinical Trial unless Sponsor provides written permission to dispose of them or, requires their retention for an additional period of time because of applicable laws, regulations and/or guidelines.

11.7 Confidentiality of study documents and patient records

The investigator must ensure anonymity of the patients; patients must not be identified by names in any documents submitted to Novartis. Signed informed consent forms and patient enrollment log must be kept strictly confidential to enable patient identification at the site.

11.8 Audits and inspections

Source data/documents must be available to inspections by Novartis or designee or Health Authorities.

11.9 Financial disclosures

Financial disclosures should be provided by study personnel who are directly involved in the treatment or evaluation of patients at the site - prior to study start.

12 Protocol adherence

Investigators ascertain they will apply due diligence to avoid protocol deviations. Under no circumstances should the investigator contact Novartis or its agents, if any, monitoring the study to request approval of a protocol deviation, as no authorized deviations are permitted. If the investigator feels a protocol deviation would improve the conduct of the study this must be considered a protocol amendment, and unless such an amendment is agreed upon by Novartis and approved by the IRB/IEC/REB it cannot be implemented. All significant protocol deviations will be recorded and reported in the CSR.

12.1 Amendments to the protocol

Any change or addition to the protocol can only be made in a written protocol amendment that must be approved by Novartis, Health Authorities where required, and the IRB/IEC/REB. Only amendments that are required for patient safety may be implemented prior to IRB/IEC/REB approval. Notwithstanding the need for approval of formal protocol amendments, the investigator is expected to take any immediate action required for the safety of any patient included in this study, even if this action represents a deviation from the protocol. In such cases, Novartis should be notified of this action and the IRB/IEC at the study site should be informed according to local regulations (e.g. UK requires the notification of urgent safety measures within 3 days) but not later than 10 working days.

[redacted]

13 References (available upon request)

Abraham RT, et al (2007) The Mammalian Target of Rapamycin Signaling Pathway: Twists and Turns in the Road to Cancer Therapy. Clin Cancer Res 13; 3109-3114.

Awada A, et al (2008) The oral mTOR inhibitor RAD001 (everolimus) in combination with letrozole in patients with advanced breast cancer: results of a phase I study with pharmacokinetics. Eur J Cancer. 2008 Jan;44(1):84-91.

Bachelot T, et al (2012) Randomized phase II trial of everolimus in combination with tamoxifen in patients with hormone receptor-positive, human epidermal growth factor receptor 2-negative metastatic breast cancer with prior exposure to aromatase inhibitors: a GINECO study. J Clin Oncol. 2012 Aug 1;30(22):2718-24.

Baselga J, et al (2009) Phase II randomized study of neoadjuvant everolimus plus letrozole compared with placebo plus letrozole in patients with estrogen receptor-positive breast cancer. J Clin Oncol. 2009 Jun 1;27(16):2630-7.

Beslija S, et al (2009) Third consensus on medical treatment of metastatic breast cancer. Ann Oncol. 2009 Nov;20(11):1771-85.

Beuvink I, et al (2005) The mTOR Inhibitor RAD001 Sensitizes Tumor Cells to DNA-Damaged Induced Apoptosis through Inhibition of p21 Translation; Cell; vol 120 747-759.

Bjornsti M-A, et al (2004) The TOR pathway: A target for cancer chemotherapy Nature Reviews Cancer 4:335-48.

Boulay A1, Lane HA (2007) Recent Results Cancer Res. The mammalian target of rapamycin kinase and tumor growth inhibition. 2007;172:99-124.

Burstein HJ, et al (2011) Novel agents and future directions for refractory breast cancer. Semin Oncol. 2011 Jun;38 Suppl 2:S17-24.

Buzdar A (2003) Pharmacology and Pharmacokinetics of the Newer Generation Aromatase Inhibitors. Clinical Cancer Research 9, 468s-472s, (Suppl.).

Campbell RA, et al (2001) Phosphatidylinositol 3- kinase/AKTmediated activation of estrogen receptor alpha: a new model for anti-estrogen resistance. J Biol Chem;276:9817–9824.

Cardoso F, et al (2012) 1st International consensus guidelines for advanced breast cancer (ABC 1). Breast. 2012 Jun;21(3):242-52.

Clopper, et al (1934) The use of confidence or fiducial limits illustrated in the case of the binomial. Biometrika 26: 404-413.

Crowe A, et al (1998) In vitro and in situ absorption of SDZ-RAD using a human intestinal cell line (Caco-2) and a single pass perfusion model in rats: comparison with rapamycin. Pharm Res. 1998 Nov;15(11):1666-72.

Demers LM, Lipton A, Harvey HA, et al (1993) The efficacy of CGS 20267 in suppressing estrogen biosynthesis in patients with advanced stage breast cancer. J Steroid Biochem Mol Biol 44: 687–691.

[redacted]

Efeyan A, Sabatini DM (2010) mTOR and cancer: many loops in one pathway. Curr Opin Cell Biol. 2010 Apr;22(2):169-76.

Eisenhauer EA, et al (2009) New response evaluation criteria in solid tumours: revised RECIST guideline (version 1.1). Eur J Cancer. 2009 Jan;45(2):228-47. doi: 10.1016/j.ejca.2008.10.026.

Ellard SL, et al (2009) Randomized phase II study comparing two schedules of everolimus in patients with recurrent/metastatic breast cancer: NCIC Clinical Trials Group IND.163. J Clin Oncol. 2009 Sep 20;27(27):4536-41.

Frattini M, et al (2005) Phosphatase protein homologue to tensin expression and phosphatidylinositol-3 phosphate kinase mutations in colorectal cancer. Cancer Res. 2005 Dec 1;65(23):11227.

GLOBOCAN 2008: Cancer Incidence and Mortality Worldwide http://www.iarc.fr/en/media-centre/iarcnews/2010/globocan2008.php.

Goel A, et al (2004) Frequent inactivation of PTEN by promoter hypermethylation in microsatellite instability-high sporadic colorectal cancers. Cancer Res. 2004 May 1;64(9):3014-21.

Guo H, et al (2007) Regulation of hepatitis B virus replication by the phosphatidylinositol 3-kinase-akt signal transduction pathway. J Virol. 2007 Sep;81(18):10072-80.

Jemal A, et al (2011) Global cancer statistics; CA Cancer J Clin. 2011 Mar-Apr;61(2):69-90.

Johnston SR (2006) Clinical efforts to combine endocrine agents with targeted therapies against epidermal growth factor receptor/human epidermal growth factor receptor 2 and mammalian target of rapamycin in breast cancer. Clin Cancer Res. 2006 Feb 1;12(3 Pt 2):1061s-1068s.

Kuhn B, et al (2001) Metabolism of sirolimus and its derivative everolimus by cytochrome P450 3A4: insights from docking, molecular dynamics, and quantum chemical calculations. J Med Chem. 2001 Jun 7;44(12):2027-34.

Laplante A, et al (2002) Interaction of immunosuppressive agents rapamycin and its analogue SDZ-RAD with endothelial P-gp. Transplant Proc. 2002 Dec;34(8):3393-5.

Linos E, et al (2008) Effects of reproductive and demographic changes on breast cancer incidence in China: a modeling analysis; J Natl Cancer Inst. 2008 Oct 1;100(19):1352-60.

Miller TW, et al (2010) Hyperactivation of phosphatidylinositol-3 kinase promotes escape from hormone dependence in estrogen receptor-positive human breast cancer. J Clin Invest. 2010 Jul;120(7):2406-13.

Noguchi S, et al (2014) Efficacy of everolimus with exemestane versus exemestane alone in Asian patients with HER2-negative, hormone-receptor-positive breast cancer in BOLERO-2. Breast Cancer. 2014 Nov;21(6):703-14.

Piccart M, et al (2014) Everolimus plus exemestane for hormone-receptor-positive, human epidermal growth factor receptor-2-negative advanced breast cancer: overall survival results from BOLERO-2. Ann Oncol. 2014 Dec;25(12):2357-62.

Plourde PV, Dyroff M, Dowsett M, et al (1995) ARIMIDEX: a new oral, once-a-day aromatase inhibitor. J Steroid Biochem Mol Biol 53: 175–179.

[redacted]

Santen RJ, et al (2005) Adaptive hypersensitivity to estrogen: mechanisms and clinical relevance to aromatase inhibitor therapy in breast cancer treatment. J Steroid Biochem Mol Biol 95:155–165.

Sarbassov DD, Ali SM, Kim D-H, et al (2004) Rictor, a Novel Binding Partner of mTOR, Defines a Rapamycin-Insensitive and Raptor-Independent Pathway that Regulates the Cytoskeleton Current Biol; 14:1296–302.

Schiff R, et al (2004) Cross-talk between estrogen receptor and growth factor pathways as a molecular target for overcoming endocrine resistance. Clin Cancer Res. 2004 Jan 1;10(1 Pt 2):331S-6S.

Tabernero J, et al (2008) Dose- and schedule-dependent inhibition of the mammalian target of rapamycin pathway with everolimus: a phase I tumor pharmacodynamic study in patients with advanced solid tumors. J Clin Oncol. 2008 Apr 1;26(10):1603-10.

Therasse P, et al (2000) New guidelines to evaluate the response to treatment in solid tumors. European Organization for Research and Treatment of Cancer, National Cancer Institute of the United States, National Cancer Institute of Canada. J Natl Cancer Inst. 2000 Feb 2;92(3):205-16.

Tokunaga E, et al (2006) Akt is frequently activated in HER2/neu-positive breast cancers and associated with poor prognosis among hormone-treated patients. Int J Cancer;118:284–289.

Velho S, et al (2005) The prevalence of PIK3CA mutations in gastric and colon cancer. Eur J Cancer. 2005 Jul;41(11):1649-54.

Yamnik RL, et al (2010) mTOR/S6K1 and MAPK/RSK signaling pathways coordinately regulate estrogen receptor alpha serine 167 phosphorylation. FEBS Lett. 2010 Jan 4;584(1):124-8.

Yamnik, et al (2009) S6 kinase 1 regulates estrogen receptor alpha in control of breast cancer cell proliferation.J Biol Chem. 2009 Mar 6;284(10):6361-9.

Yardley DA, et al (2013) Everolimus plus exemestane in postmenopausal patients with HR(+) breast cancer: BOLERO-2 final progression-free survival analysis. Adv Ther. 2013 Oct;30(10):870-84.

Yeo W, et al (2004) Comprehensive analysis of risk factors associating with Hepatitis B virus (HBV) reactivation in cancer patients undergoing cytotoxic chemotherapy. Br J Cancer. 2004 Apr 5;90(7):1306-11.

Yue W, et al (2007) Mechanisms of acquired resistance to endocrine therapy in hormone-dependent breast cancer cells. J Steroid Biochem Mol Biol; 106:102-110.

[redacted]

14 Appendices

14.1 Appendix 1: Guidelines for Response, Duration of Overall Response, TTF, TTP, Progression-Free Survival and Overall Survival (version 3.2, based on RECIST 1.1)

Glossary

CR	Complete response
CRF	Case Report Form
CSR	Clinical Study Report
CT	Computed tomography
DFS	Disease-free survival
eCRF	Electronic Case Report Form
FPFV	First patient first visit
GBM	Glioblastoma multiforme
MRI	Magnetic resonance imaging
LPLV	Last patient last visit
OS	Overall survival
PD	Progressive disease
PFS	Progression-free survival
PR	Partial response
RAP	Reporting and Analysis Plan
RECIST	Response Evaluation Criteria in Solid Tumors
SD	Stable disease
SOD	Sum of Diameter
TTF	Time to treatment failure
TTP	Time to progression
UNK	Unknown

[redacted]

14.1.1 Introduction

The purpose of this document is to provide the working definitions and rules necessary for a consistent and efficient analysis of efficacy for oncology studies in solid tumors. This document is based on the RECIST criteria for tumor responses (Therasse et al 2000) and the revised RECIST 1.1 guidelines (Eisenhauer et al 2009).

The efficacy assessments described in Section 14.1.2 and the definition of best response in Section 14.1.3.1 are based on the RECIST 1.1 criteria but also give more detailed instructions and rules for determination of best response. Section 14.1.3.2 is summarizing the "time to event" variables and rules which are mainly derived from internal discussions and regulatory consultations, as the RECIST criteria do not define these variables in detail. Section 14.1.4 of this guideline describes data handling and programming rules. This section is to be referred to in the SAP (Statistical Analysis Plan) to provide further details needed for programming.

14.1.2 Efficacy assessments

Tumor evaluations are made based on RECIST criteria (Therasse et al 2000), New Guidelines to Evaluate the Response to Treatment in Solid Tumors, Journal of National Cancer Institute, Vol. 92; 205-16 and revised RECIST guidelines (version 1.1) (Eisenhauer et al 2009) European Journal of Cancer; 45:228-247.

14.1.2.1 Definitions

14.1.2.1.1 Disease measurability

In order to evaluate tumors throughout a study, definitions of measurability are required in order to classify lesions appropriately at baseline. In defining measurability, a distinction also needs to be made between nodal lesions (pathological lymph nodes) and non-nodal lesions.

- **Measurable disease** - the presence of at least one measurable nodal or non-nodal lesion. If the measurable disease is restricted to a solitary lesion, its neoplastic nature should be confirmed by cytology/histology.

For patients without measurable disease see Section 14.1.3.2.8.

Measurable lesions (both nodal and non-nodal)

- Measurable non-nodal - As a rule of thumb, the minimum size of a measurable non-nodal target lesion at baseline should be no less than double the slice thickness or 10mm whichever is greater - e.g. the minimum non-nodal lesion size for CT/MRI with 5mm cuts will be 10 mm, for 8 mm contiguous cuts the minimum size will be 16 mm.
- Lytic bone lesions or mixed lytic-blastic lesions with identifiable soft tissue components, that can be evaluated by CT/MRI, can be considered as measurable lesions, if the soft tissue component meets the definition of measurability.
- Measurable nodal lesions (i.e. lymph nodes) - Lymph nodes □15 mm in short axis can be considered for selection as target lesions. Lymph nodes measuring 10 mm and <15 mm are considered non-measurable. Lymph nodes smaller than 10 mm in short axis at baseline, regardless of the slice thickness, are normal and not considered indicative of disease.

[redacted]

- Cystic lesions:
  - Lesions that meet the criteria for radiographically defined simple cysts (i.e., spherical structure with a thin, non-irregular, non-nodular and non-enhancing wall, no septations, and low CT density [water-like] content) should not be considered as malignant lesions (neither measurable nor non-measurable) since they are, by definition, simple cysts.
  - 'Cystic lesions' thought to represent cystic metastases can be considered as measurable lesions, if they meet the definition of measurability described above. However, if noncystic lesions are present in the same patient, these are preferred for selection as target lesions.
- Non-measurable lesions - all other lesions are considered non-measurable, including small lesions (e.g. longest diameter <10 mm with CT/MRI or pathological lymph nodes with 10 to < 15 mm short axis), as well as truly non-measurable lesions e.g., blastic bone lesions, leptomeningeal disease, ascites, pleural/pericardial effusion, inflammatory breast disease, lymphangitis cutis/pulmonis, abdominal masses/abdominal organomegaly identified by physical exam that is not measurable by reproducible imaging techniques.

14.1.2.1.2 Eligibility based on measurable disease

If no measurable lesions are identified at baseline, the patient may be allowed to enter the study in some situations (e.g. in Phase III studies where PFS is the primary endpoint). However, it is recommended that patients be excluded from trials where the main focus is on the Overall Response Rate (ORR). Guidance on how patients with just non-measurable disease at baseline will be evaluated for response and also handled in the statistical analyses is given in Section 14.1.3.2.9.

14.1.2.2 Methods of tumor measurement - general guidelines

In this document, the term "contrast" refers to intravenous (i.v.) contrast. The following considerations are to be made when evaluating the tumor:

- All measurements should be taken and recorded in metric notation (mm), using a ruler or calipers. All baseline evaluations should be performed as closely as possible to the beginning of treatment and never more than 4 weeks before the beginning of the treatment.
- Imaging-based evaluation is preferred to evaluation by clinical examination when both methods have been used to assess the antitumor effect of a treatment.
- For optimal evaluation of patients, the same methods of assessment and technique should be used to characterize each identified and reported lesion at baseline and during follow-up. Contrast-enhanced CT of chest, abdomen and pelvis should preferably be performed using a 5 mm slice thickness with a contiguous reconstruction algorithm. CT/MRI scan slice thickness should not exceed 8 mm cuts using a contiguous reconstruction algorithm. If, at baseline, a patient is known to have a medical contraindication to CT contrast or develops a contraindication during the trial, the following change in imaging modality will be accepted for follow up: a non-contrast CT of chest (MRI not recommended due to respiratory artifacts) plus contrast-enhanced MRI of abdomen and pelvis.

[redacted]

- A change in methodology can be defined as either a change in contrast use (e.g. keeping the same technique, like CT, but switching from with to without contrast use or vice-versa, regardless of the justification for the change) or a major change in technique (e.g. from CT to MRI, or vice-versa), or a change in any other imaging modality. A change from conventional to spiral CT or vice versa will not constitute a major “change in method” for the purposes of response assessment. A change in methodology will result by default in a UNK overall lesion response assessment as per Novartis calculated response. However, another response assessment than the Novartis calculated UNK response may be accepted from the investigator or the central blinded reviewer if a definitive response assessment can be justified, based on the available information.
- FDG-PET: can complement CT scans in assessing progression (particularly possible for ‘new’ disease). New lesions on the basis of FDG-PET imaging can be identified according to the following algorithm:
  - Negative FDG-PET at baseline, with a positive FDG-PET at follow-up is a sign of PD based on a new lesion.
  - No FDG-PET at baseline with a positive FDG-PET at follow-up:
- If new disease is indicated by a positive PET scan but is not confirmed by CT (or some other conventional technique such as MRI) at the same assessment, then follow-up assessments by CT will be needed to determine if there is truly progression occurring at that site. In all cases PD will be the date of confirmation of new disease by CT (or some other conventional technique such as MRI) rather than the date of the positive PET scan. If there is a positive PET scan without any confirmed progression at that site by CT, then aPD cannot be assigned.
  - If the positive FDG-PET at follow-up corresponds to a pre-existing site of disease on CT that is not progressing on the basis of the anatomic images, this is not PD.
  - Chest x-ray: Lesions on chest x-ray are acceptable as measurable lesions when they are clearly defined and surrounded by aerated lung. However, CT is preferable.
  - Physical exams: Evaluation of lesions by physical examination is accepted when lesions are superficial, with at least 10mm size, and can be assessed using calipers.
  - Ultrasound: When the primary endpoint of the study is objective response evaluation, ultrasound (US) should not be used to measure tumor lesions, unless pre-specified by the protocol. It is, however, a possible alternative to clinical measurements of superficial palpable lymph nodes, subcutaneous lesions and thyroid nodules. US might also be useful to confirm the complete disappearance of superficial lesions usually assessed by clinical examination.
  - Endoscopy and laparoscopy: The utilization of endoscopy and laparoscopy for objective tumor evaluation has not yet been fully and widely validated. Their uses in this specific context require sophisticated equipment and a high level of expertise that may only be available in some centers. Therefore, the utilization of such techniques for objective tumor response should be restricted to validation purposes in specialized centers. However, such techniques can be useful in confirming complete pathological response when biopsies are obtained.

[redacted]

- Tumor markers: Tumor markers alone cannot be used to assess response. However, some disease specific and more validated tumor markers (e.g. CA-125 for ovarian cancer, PSA for prostate cancer, alpha-FP, LDH and Beta-hCG for testicular cancer) can be integrated as non-target disease. If markers are initially above the upper normal limit they must normalize for a patient to be considered in complete clinical response when all lesions have disappeared.
- Cytology and histology: Cytology and histology can be used to differentiate between PR and CR in rare cases (i.e., after treatment to differentiate between residual benign lesions and residual malignant lesions in tumor types such as germ cell tumors). Cytologic confirmation of neoplastic nature of any effusion that appears or worsens during treatment is required when the measurable tumor has met the criteria for response or stable disease. Under such circumstances, the cytologic examination of the fluid collected will permit differentiation between response and stable disease (an effusion may be a side effect of the treatment) or progressive disease (if the neoplastic origin of the fluid is confirmed).
- Clinical examination: Clinical lesions will only be considered measurable when they are superficial (i.e., skin nodules and palpable lymph nodes). For the case of skin lesions, documentation by color photography, including a ruler to estimate the size of the lesion, is recommended.

14.1.2.3 Baseline documentation of target and non-target lesions

For the evaluation of lesions at baseline and throughout the study, the lesions are classified at baseline as either target or non-target lesions:

- Target lesions: All measurable lesions (nodal and non-nodal) up to a maximum of five lesions in total (and a maximum of two lesions per organ), representative of all involved organs should be identified as target lesions and recorded and measured at baseline. Target lesions should be selected on the basis of their size (lesions with the longest diameter) and their suitability for accurate repeated measurements (either by imaging techniques or clinically). Each target lesion must be uniquely and sequentially numbered on the CRF (even if it resides in the same organ).

Minimum target lesion size at baseline

- **Non-nodal target:** Non-nodal target lesions identified by methods for which slice thickness is not applicable (e.g. clinical examination, photography) should be at least 10 mm in longest diameter. See Section 14.1.2.1.1.
- **Nodal target:** See Section 14.1.2.1.1.

A sum of diameters (long axis for non-nodal lesions, short axis for nodal) for all target lesions will be calculated and reported as the baseline sum of diameters (SOD). The baseline sum of diameters will be used as reference by which to characterize the objective tumor response. Each target lesion identified at baseline must be followed at each subsequent evaluation and documented on eCRF.

- **Non-target lesions:** All other lesions are considered non-target lesions, i.e. lesions not fulfilling the criteria for target lesions at baseline. Presence or absence or worsening of non-target lesions should be assessed throughout the study; measurements of these lesions

[redacted]

are not required. Multiple non-target lesions involved in the same organ can be assessed as a group and recorded as a single item (i.e. multiple liver metastases). Each non-target lesion identified at baseline must be followed at each subsequent evaluation and documented on eCRF.

14.1.2.4 Follow-up evaluation of target and non-target lesions

To assess tumor response, the sum of diameters for all target lesions will be calculated (at baseline and throughout the study). At each assessment response is evaluated first separately for the target (Table 14-1) and non-target lesions (Table 14-2) identified at baseline. These evaluations are then used to calculate the overall lesion response considering both the target and non-target lesions together (Table 14-3) as well as the presence or absence of new lesions.

14.1.2.4.1 Follow-up and recording of lesions

At each visit and for each lesion the actual date of the scan or procedure which was used for the evaluation of each specific lesion should be recorded. This applies to target and non-target lesions as well as new lesions that are detected. At the assessment visit all of the separate lesion evaluation data are examined by the investigator in order to derive the overall visit response. Therefore all such data applicable to a particular visit should be associated with the same assessment number.

14.1.2.4.1.1 Non-nodal lesions

Following treatment, lesions may have longest diameter measurements smaller than the image reconstruction interval. Lesions smaller than twice the reconstruction interval are subject to substantial “partial volume” effects (i.e., size may be underestimated because of the distance of the cut from the longest diameter; such lesions may appear to have responded or progressed on subsequent examinations, when, in fact, they remain the same size).

If the lesion has completely disappeared, the lesion size should be reported as 0 mm. Measurements of non-nodal target lesions that become 5 mm or less in longest diameter are likely to be non-reproducible. Therefore, it is recommended to report a default value of 5 mm, instead of the actual measurement. This default value is derived from the 5 mm CT slice thickness (but should not be changed with varying CT slice thickness). Actual measurement should be given for all lesions larger than 5 mm in longest diameter irrespective of slice thickness/reconstruction interval.

In other cases where the lesion cannot be reliably measured for reasons other than its size (e.g., borders of the lesion are confounded by neighboring anatomical structures), no measurement should be entered and the lesion cannot be evaluated.

14.1.2.4.1.2 Nodal lesions

A nodal lesion less than 10 mm in size by short axis is considered normal. Lymph nodes are not expected to disappear completely, so a “non-zero size” will always persist.

Measurements of nodal target lesions that become 5 mm or less in short axis are likely to be non-reproducible. Therefore, it is recommended to report a default value of 5 mm, instead of the actual measurement. This default value is derived from the 5 mm CT slice thickness (but

[redacted]

should not be changed with varying CT slice thickness).Actual measurement should be given for all lesions larger than 5 mm in short axis irrespective of slice thickness/reconstruction interval.

However, once a target nodal lesion shrinks to less than 10 mm in its short axis, it will be considered normal for response purpose determination. The lymph node measurements will continue to be recorded to allow the values to be included in the sum of diameters for target lesions, which may be required subsequently for response determination.

14.1.2.4.2 Determination of target lesion response

Table 14-1 Response criteria for target lesions

Response Criteria	Evaluation of target lesions
Complete Response (CR):	Disappearance of all non-nodal target lesions. In addition, any pathological lymph nodes assigned as target lesions must have a reduction in short axis to < 10 mm.[1]
Partial Response (PR):	At least a 30% decrease in the sum of diameter of all target lesions, taking as reference the baseline sum of diameters.
Progressive Disease (PD):	At least a 20% increase in the sum of diameter of all measured target lesions, taking as reference the smallest sum of diameter of all target lesions recorded at or after baseline. In addition to the relative increase of 20%, the sum must also demonstrate an absolute increase of at least 5 mm.[2]
Stable Disease (SD):	Neither sufficient shrinkage to qualify for PR or CR nor an increase in lesions which would qualify for PD.
Unknown (UNK)	Progression has not been documented and one or more target lesions have not been assessed or have been assessed using a different method than baseline.[3]

1. SOD for CR may not be zero when nodal lesions are part of target lesions
2. Following an initial CR, a PD cannot be assigned if all non-nodal target lesions are still not present and all nodal lesions are <10 mm in size. In this case, the target lesion response is CR
3. In exceptional circumstances an UNK response due to change in method could be over-ruled by the investigator or central reviewer using expert judgment based on the available information (see Notes on target lesion response and methodology change in Section 14.1.2.2).

Notes on target lesion response

Reappearance of lesions: If the lesion appears at the same anatomical location where a target lesion had previously disappeared, it is advised that the time point of lesion disappearance (i.e., the “0 mm” recording) be re-evaluated to make sure that the lesion was not actually present and/or not visualized for technical reasons in this previous assessment. If it is not possible to change the 0 value, then the investigator/radiologist has to decide between the following possibilities:

- The lesion is a new lesion, in which case the overall tumor assessment will be considered as progressive disease
- The lesion is clearly a reappearance of a previously disappeared lesion, in which case the size of the lesion has to be entered in the CRF and the tumor assessment will remain based on the sum of tumor measurements as presented in Table 14-1 above (i.e., a PD will be determined if there is at least 20% increase in the sum of diameters of **all** measured target lesions, taking as reference the smallest sum of diameters of all target lesions recorded at or after baseline with at least 5 mm increase in the absolute sum of the diameters). Proper documentation should be available to support this decision. This applies to patients who

[redacted]

have not achieved target response of CR. For patients who have achieved CR, please refer to last bullet in this section.

- For those patients who have only one target lesion at baseline, the reappearance of the target lesion which disappeared previously, even if still small, is considered a PD.
- Missing measurements: In cases where measurements are missing for one or more target lesions it is sometimes still possible to assign PD based on the measurements of the remaining lesions. For example, if the sum of diameters for 5 target lesions at baseline is
- 100 mm at baseline and the sum of diameters for 3 of those lesions at a post-baseline visit is 140 mm (with data for 2 other lesions missing) then a PD should be assigned. However, in other cases where a PD cannot definitely be attributed, the target lesion response would be UNK.
- Nodal lesion decrease to normal size: When nodal disease is included in the sum of target lesions and the nodes decrease to "normal" size they should still have a measurement recorded on scans. This measurement should be reported even when the nodes are normal in order not to overstate progression should it be based on increase in the size of nodes.
- Lesions split: In some circumstances, disease that is measurable as a target lesion at baseline and appears to be one mass can split to become two or more smaller sub-lesions. When this occurs, the diameters (long axis - non-nodal lesion, short axis - nodal lesions) of the two split lesions should be added together and the sum recorded in the diameter field on the case report form under the original lesion number. This value will be included in the sum of diameters when deriving target lesion response. The individual split lesions will not be considered as new lesions, and will not automatically trigger a PD designation.
- Lesions coalesced: Conversely, it is also possible that two or more lesions which were distinctly separate at baseline become confluent at subsequent visits. When this occurs a plane between the original lesions may be maintained that would aid in obtaining diameter measurements of each individual lesion. If the lesions have truly coalesced such that they are no longer separable, the maximal diameters (long axis - non-nodal lesion, short axis - nodal lesions) of the "merged lesion" should be used when calculating the sum of diameters for target lesions. On the case report form, the diameter of the "merged lesion" should be recorded for the size of one of the original lesions while a size of "0"mm should be entered for the remaining lesion numbers which have coalesced.
- The **measurements for nodal lesions**, even if less than 10 mm in size, will contribute to the calculation of target lesion response in the usual way with slight modifications.
- Since lesions less than 10 mm are considered normal, a CR for target lesion response should be assigned when all nodal target lesions shrink to less than 10 mm and all non-nodal target lesions have disappeared.
- Once a CR target lesion response has been assigned a CR will continue to be appropriate (in the absence of missing data) until progression of target lesions.
- Following a CR, a PD can subsequently only be assigned for target lesion response if either a non-nodal target lesion "reappears" or if any single nodal lesion is at least 10 mm and there is at least 20% increase in sum of the diameters of all nodal target lesions relative to nadir with at least 5 mm increase in the absolute sum of the diameters.

[redacted]

- A change in method for the evaluation of one or more lesions will usually lead to an UNK target lesion response unless there is progression indicated by the remaining lesions which have been evaluated by the same method. In exceptional circumstances an investigator or central reviewer might over-rule this assignment to put a non-UNK response using expert judgment based on the available information. E.g. a change to a more sensitive method might indicate some tumor shrinkage of target lesions and definitely rule out progression in which case the investigator might assign an SD target lesion response; however, this should be done with caution and conservatively as the response categories have well defined criteria.

14.1.2.4.3 Determination of non-target lesion response

Table 14-2 Response criteria for non-target lesions

Response Criteria	Evaluation of non-target lesions
Complete Response (CR):	Disappearance of all non-target lesions. In addition, all lymph nodes assigned a non-target lesions must be non-pathological in size (< 10 mm short axis)
Progressive Disease (PD):	Unequivocal progression of existing non-target lesions.[1]
Non-CR/Non-PD:	Neither CR nor PD
Unknown (UNK)	Progression has not been documented and one or more non-target lesions have not been assessed or have been assessed using a different method than baseline[2].

1. The assignment of PD solely based on change in non-target lesions in light of target lesion response of CR, PR or SD should be exceptional. In such circumstances, the opinion of the investigator or central reviewer does prevail
2. It is recommended that the investigator and/or central reviewer should use expert judgment to assign a Non-UNK response wherever possible (see notes section for more details)

Notes on non-target lesion response

- The investigator and/or central reviewer can use expert judgment to assign a non-UNK response wherever possible, even where lesions have not been fully assessed or a different method has been used. In many of these situations it may still be possible to identify equivocal progression (PD) or definitively rule this out (non-CR/Non-PD) based on the available information. In the specific case where a more sensitive method has been used indicating the absence of any non-target lesions, a CR response can also be assigned.
- The response for non-target lesions is **CR** only if all non-target non-nodal lesions which were evaluated at baseline are now all absent and with all non-target nodal lesions returned to normal size (i.e. < 10 mm). If any of the non-target lesions are still present, or there are any abnormal nodal lesions (i.e. ≥ 10 mm) the response can only be '**Non-CR/Non-PD**' unless there is unequivocal progression of the non-target lesions (in which case response is **PD**) or it is not possible to determine whether there is unequivocal progression (in which case response is UNK).
- Unequivocal progression: To achieve "unequivocal progression" on the basis of non-target disease there must be an overall level of substantial worsening in non-target disease such that, even in presence of CR, PR or SD in target disease, the overall tumor burden has increased sufficiently to merit discontinuation of therapy. A modest "increase" in the size of one or more non-target lesions is usually not sufficient to qualify for unequivocal progression status. The designation of overall progression solely on the basis of change in

[redacted]

non-target disease in the face of CR, PR or SD of target disease is therefore expected to be rare. In order for a PD to be assigned on the basis of non-target lesions, the increase in the extent of the disease must be substantial even in cases where there is no measurable

- disease at baseline. If there is unequivocal progression of non-target lesion(s), then at least one of the non-target lesions must be assigned a status of "Worsened". Where possible, similar rules to those described in Section 14.1.2.4.4 for assigning PD following a CR for the non-target lesion response in the presence of non-target lesions nodal lesions should be applied.

14.1.2.4.4 New lesions

The appearance of a new lesion is always associated with Progressive Disease (PD) and has to be recorded as a new lesion in the New Lesion CRF page.

- If a new lesion is **equivocal**, for example because of its small size, continued therapy and follow-up evaluation will clarify if it represents truly new disease. If repeat scans confirm there is definitely a new lesion, then progression should be declared using the date of the first observation of the lesion
- If new disease is observed in a region which was **not scanned at baseline** or where the particular baseline scan is not available for some reason, then this should be considered as a PD. The one exception to this is when there are no baseline scans at all available for a patient in which case the response should be UNK, as for any of this patient's assessment (see Section 14.1.2.5).
- A **lymph node is considered as a "new lesion"** and, therefore, indicative of progressive disease if the short axis increases in size to ≥ 10 mm for the first time in the study plus 5 mm absolute increase.
  FDG-PET: can complement CT scans in assessing progression (particularly possible for 'new' disease). See Section 14.1.2.2.

14.1.2.5 Evaluation of overall lesion response

The evaluation of overall lesion response at each assessment is a composite of the target lesion response, non-target lesion response and presence of new lesions as shown below in Table 14-3.

[redacted]

Table 14-3 Overall lesion response at each assessment

Target lesions	Non-target lesions	New Lesions	Overall lesion response
CR	CR	No	CR[1]
CR	Non-CR/Non-PD[3]	No	PR
CR, PR, SD	UNK	No	UNK
PR	Non-PD and not UNK	No	PR[1]
SD	Non-PD and not UNK	No	SD[1, 2]
UNK	Non-PD or UNK	No	UNK[1]
PD	Any	Yes or No	PD
Any	PD	Yes or No	PD
Any	Any	Yes	PD

1. This overall lesion response also applies when there are no non-target lesions identified at baseline.
2. Once confirmed PR was achieved, all these assessments are considered PR.
3. As defined in Section 14.1.2.4.

If there are no baseline scans available at all, then the overall lesion response at each assessment should be considered Unknown (UNK).

In some circumstances it may be difficult to distinguish residual disease from normal tissue. When the evaluation of complete response depends on this determination, it is recommended that the residual lesion be investigated (fine needle aspirate/biopsy) to confirm the CR.

14.1.3 Efficacy definitions

The following definitions primarily relate to patients who have measurable disease at baseline. Section 14.1.3.2.9 outlines the special considerations that need to be given to patients with no measurable disease at baseline in order to apply the same concepts.

14.1.3.1 Best overall response

The best overall response is the best response recorded from the start of the treatment until disease progression/recurrence (taking as reference for PD the smallest measurements recorded since the treatment started). In general, the patient's best response assignment will depend on the achievement of both measurement and confirmation criteria.

The best overall response will usually be determined from response assessments undertaken while on treatment. However, if any assessments occur after treatment withdrawal the protocol should specifically describe if these will be included in the determination of best overall response and/or whether these additional assessments will be required for sensitivity or supportive analyses. As a default, any assessments taken more than 30 days after the last dose of study treatment will not be included in the best overall response derivation. If any alternative cancer therapy is taken while on study any subsequent assessments would ordinarily be excluded from the best overall response determination. If response assessments taken after withdrawal from study treatment and/or alternative therapy are to be included in the main endpoint determination, then this should be described and justified in the protocol.

Where a study requires confirmation of response (PR or CR), changes in tumor measurements must be confirmed by repeat assessments that should be performed not less than 4 weeks after the criteria for response are first met.

[redacted]

Longer intervals may also be appropriate. However, this must be clearly stated in the protocol. The main goal of confirmation of objective response is to avoid overestimating the response rate observed. In cases where confirmation of response is not feasible, it should be made clear when reporting the outcome of such studies that the responses are not confirmed.

- For non-randomized trials where response is the primary endpoint, confirmation is needed.
- For trials intended to support accelerated approval, confirmation is needed
- For all other trials, confirmation of response may be considered optional.

The best overall response for each patient is determined from the sequence of overall (lesion) responses according to the following rules:

- CR = at least two determinations of CR at least 4 weeks apart before progression where confirmation required or one determination of CR prior to progression where confirmation not required
- PR = at least two determinations of PR or better at least 4 weeks apart before progression (and not qualifying for a CR) where confirmation required or one determination of PR prior to progression where confirmation not required
- SD = at least one SD assessment (or better) > 6 weeks after randomization/start of treatment (and not qualifying for CR or PR).
- PD = progression ≤ 12 weeks after randomization/ start of treatment (and not qualifying for CR, PR or SD).
- UNK = all other cases (i.e. not qualifying for confirmed CR or PR and without SD after more than 6 weeks or early progression within the first 12 weeks)

The time durations specified in the SD/PD/UNK definitions above are defaults based on a 6 week tumor assessment frequency. However these may be modified for specific indications which are more or less aggressive. In addition, it is envisaged that the time duration may also take into account assessment windows. E.g. if the assessment occurs every 6 weeks with a time window of +/- 7 days, a BOR of SD would require a SD or better response longer than 5 weeks after randomization/start of treatment.

Overall lesion responses of CR must stay the same until progression sets in, with the exception of a UNK status. A patient who had a CR cannot subsequently have a lower status other than a PD, e.g. PR or SD, as this would imply a progression based on one or more lesions reappearing, in which case the status would become a PD.

Once an overall lesion response of PR is observed (which may have to be a confirmed PR depending on the study) this assignment must stay the same or improve over time until progression sets in, with the exception of an UNK status. However, in studies where confirmation of response is required, if a patient has a single PR (≥30% reduction of tumor burden compared to baseline) at one assessment, followed by a <30% reduction from baseline at the next assessment (but not ≥20% increase from previous smallest sum), the objective status at that assessment should be SD. Once a confirmed PR was seen, the overall lesion response should be considered PR (or UNK) until progression is documented or the lesions totally disappear in which case a CR assignment is applicable. In studies where confirmation of response is not required after a single PR the overall lesion response should still be

[redacted]

considered PR (or UNK) until progression is documented or the lesion totally disappears in which case a CR assignment is applicable.

Example: In a case where confirmation of response is required the sum of lesion diameters is 200 mm at baseline and then 140 mm - 150 mm - 140 mm - 160 mm - 160 mm at the subsequent visits. Assuming that non-target lesions did not progress, the overall lesion response would be PR - SD - PR - PR - PR. The second assessment with 140 mm confirms the PR for this patient. All subsequent assessments are considered PR even if tumor measurements decrease only by 20% compared to baseline (200 mm to 160 mm) at the following assessments.

If the patient progressed but continues study treatment, further assessments are not considered for the determination of best overall response.

Note: these cases may be described as a separate finding in the CSR but not included in the overall response or disease control rates.

The best overall response for a patient is always calculated, based on the sequence of overall lesion responses. However, the overall lesion response at a given assessment may be provided from different sources:

- Investigator overall lesion response
- Central Blinded Review overall lesion response
- Novartis calculated overall lesion response (based on measurements from either Investigator or Central Review)

The primary analysis of the best overall response will be based on the sequence of investigator/central blinded review/calculated (investigator)/calculated (central) overall lesion responses.

Based on the patients' best overall response during the study, the following rates are then calculated:

Overall response rate (ORR) is the proportion of patients with a best overall response of CR or PR. This is also referred to as 'Objective response rate' in some protocols or publications.

Disease control rate (DCR) is the proportion of patients with a best overall response of CR or PR or SD. The objective of this endpoint is to summarize patients with signs of "activity" defined as either shrinkage of tumor (regardless of duration) or slowing down of tumor growth.

Clinical benefit rate (CBR) is the proportion of patients with a best overall response of CR or PR, or an overall lesion response of SD or Non-CR/Non-PD which lasts for a minimum time duration (with a default of at least 24 weeks in breast cancer studies). This endpoint measures signs of activity taking into account duration of disease stabilization.

Another approach is to summarize the progression rate at a certain time point after baseline. In this case, the following definition is used:

Early progression rate (EPR) is the proportion of patients with progressive disease within 8 weeks of the start of treatment.

[redacted]

The protocol should define populations for which these will be calculated. The timepoint for EPR is study specific. EPR is used for the multinomial designs of Dent and Zee (2001) and counts all patients who at the specified assessment (in this example the assessment would be at 8 weeks ± window) do not have an overall lesion response of SD, PR or CR. Patients with an unknown (UNK) assessment at that time point and no PD before, will not be counted as early progressors in the analysis but may be included in the denominator of the EPR rate, depending on the analysis population used. Similarly when examining overall response and disease control, patients with a best overall response assessment of unknown (UNK) will not be regarded as "responders" but may be included in the denominator for ORR and DCR calculation depending on the analysis population (e.g. populations based on an ITT approach).

14.1.3.2 Time to event variables

The protocol should state which of the following variables is used in that study.

14.1.3.2.1 Progression-free survival

Usually in all Oncology studies, patients are followed for tumor progression after discontinuation of study medication for reasons other than progression or death. If this is not used, e.g. in Phase I or II studies, this should be clearly stated in the protocol. Note that randomized trials (preferably blinded) are recommended where PFS is to be the primary endpoint.

Progression-free survival (PFS) is the time from date of randomization/start of treatment to the date of event defined as the first documented progression or death due to any cause. If a patient has not had an event, progression-free survival is censored at the date of last adequate tumor assessment.

PFS rate at x weeks is an additional measure used to quantify PFS endpoint. It is recommended that a Kaplan Meier estimate is used to assess this endpoint.

14.1.3.2.2 Overall survival

All patients should be followed until death or until patient has had adequate follow-up time as specified in the protocol whichever comes first. The follow-up data should contain the date the patient was last seen alive / last known date patient alive, the date of death and the reason of death ("Study indication" or "Other").

Overall survival (OS) is defined as the time from date of randomization/start of treatment to date of death due to any cause. If a patient is not known to have died, survival will be censored at the date of last known date patient alive.

14.1.3.2.3 Time to progression

Some studies might consider only death related to underlying cancer as an event which indicates progression. In this case the variable "Time to progression" might be used. TTP is defined as PFS except for death unrelated to underlying cancer.

Time to progression (TTP) is the time from date of randomization/start of treatment to the date of event defined as the first documented progression or death due to underlying cancer. If

[redacted]

a patient has not had an event, time to progression is censored at the date of last adequate tumor assessment.

14.1.3.2.4 PFS2

A recent EMA guidance (EMA 2012) recommends a substitute end point intermediate to PFS and OS called PFS2, a surrogate for OS when OS cannot be measured reliably, which assesses the impact of the experimental therapy on next-line treatment. The main purpose of this endpoint is to assess long term maintenance strategies, particularly of resensitizing agents and where it is necessary to examine the overall "field of influence".

PFS2, which could be termed PFS deferred, PFS delayed, tandem PFS, or PFS version 2.0, is the time from date of randomization/start of treatment to the date of event defined as the first documented progression on next-line treatment or death from any cause. The censoring rules for this endpoint will incorporate the same principles as those considered for PFS in this document, and in addition may involve other considerations which will need to be detailed in the protocol.

Please note that data collection for the PFS2 is limited to the date of progression and not specific read of the tumor assessments.

It is strongly recommended that the teams consult regulatory agencies for scientific advice given the limited experience with the use of this endpoint in regulatory setting in light of methodological issues w.r.t. censoring foreseen.

14.1.3.2.5 Time to treatment failure

This endpoint is often appropriate in studies of advanced disease where early discontinuation is typically related to intolerance of the study drug. In some protocols, time to treatment failure may be considered as a sensitivity analysis for time to progression. The list of discontinuation reasons to be considered or not as treatment failure may be adapted according to the specificities of the study or the disease.

Time to treatment failure (TTF) is the time from date of randomization/start of treatment to the earliest of date of progression, date of death due to any cause, or date of discontinuation due to reasons other than 'Protocol violation' or 'Administrative problems'. The time to treatment failure for patients who did not experience treatment failure will be censored at last adequate tumor assessment.

14.1.3.2.6 Duration of response

The analysis of the following variables should be performed with much caution when restricted to responders since treatment bias could have been introduced. There have been reports where a treatment with a significantly higher response rate had a significantly shorter duration of response but where this probably primarily reflected selection bias which is explained as follows: It is postulated that there are two groups of patients: a good risk group and a poor risk group. Good risk patients tend to get into response readily (and relatively quickly) and tend to remain in response after they have a response. Poor risk patients tend to be difficult to achieve a response, may have a longer time to respond, and tend to relapse quickly when they do respond. Potent agents induce a response in both good risk and poor

[redacted]

risk patients. Less potent agents induce a response mainly in good risk patients only. This is described in more detail by Morgan (1988).

It is recommended that an analysis of all patients (both responders and non-responders) be performed whether or not a "responders only" descriptive analysis is presented. An analysis of responders should only be performed to provide descriptive statistics and even then interpreted with caution by evaluating the results in the context of the observed response rates… If an inferential comparison between treatments is required this should only be performed on all patients (i.e. not restricting to "responders" only) using appropriate statistical methods such as the techniques described in Ellis et al (2008). It should also be stated in the protocol if duration of response is to be calculated in addition for unconfirmed response.

For summary statistics on "responders" only the following definitions are appropriate. (Specific definitions for an all-patient analysis of these endpoints are not appropriate since the status of patients throughout the study is usually taken into account in the analysis).

Duration of overall response (CR or PR): For patients with a CR or PR (which may have to be confirmed the start date is the date of first documented response (CR or PR) and the end date and censoring is defined the same as that for time to progression.

The following two durations might be calculated in addition for a large Phase III study in which a reasonable number of responders is seen.

Duration of overall complete response (CR): For patients with a CR (which may have to be confirmed) the start date is the date of first documented CR and the end date and censoring is defined the same as that for time to progression.

Duration of stable disease (CR/PR/SD): For patients with a CR or PR (which may have to be confirmed) or SD the start and end date as well as censoring is defined the same as that for time to progression.

14.1.3.2.7 Time to response

Time to overall response (CR or PR) is the time between date of randomization/start of treatment until first documented response (CR or PR). The response may need to be confirmed depending on the type of study and its importance. Where the response needs to be confirmed then time to response is the time to the first CR or PR observed.

Although an analysis on the full population is preferred a descriptive analysis may be performed on the "responders" subset only, in which case the results should be interpreted with caution and in the context of the overall response rates, since the same kind of selection bias may be introduced as described for duration of response in Section 14.1.3.2.6. It is recommended that an analysis of all patients (both responders and non-responders) be performed whether or not a "responders only" descriptive analysis is presented. Where an inferential statistical comparison is required, then all patients should definitely be included in the analysis to ensure the statistical test is valid. For analysis including all patients, patients who did not achieve a response (which may have to be a confirmed response) will be censored using one of the following options.

- at maximum follow-up (i.e. FPFV to LPLV used for the analysis) for patients who had a PFS event (i.e. progressed or died due to any cause). In this case the PFS event is the

[redacted]

worst possible outcome as it means the patient cannot subsequently respond. Since the statistical analysis usually makes use of the ranking of times to response it is sufficient to assign the worst possible censoring time which could be observed in the study which is equal to the maximum follow-up time (i.e. time from FPFV to LPLV)

- at last adequate tumor assessment date otherwise. In this case patients have not yet progressed so they theoretically still have a chance of responding

Time to overall complete response (CR) is the time between dates of randomization/start of treatment until first documented CR. Similar analysis considerations including (if appropriate) censoring rules apply for this endpoint described for the time to overall response endpoint.

14.1.3.2.8 Definition of start and end dates for time to event variables

Assessment date

For each assessment (i.e. evaluation number), the **assessment date** is calculated as the latest of all measurement dates (e.g. X-ray, CT-scan) if the overall lesion response at that assessment is CR/PR/SD/UNK. Otherwise - if overall lesion response is progression - the assessment date is calculated as the earliest date of all measurement dates at that evaluation number.

In the calculation of the assessment date for time to event variables, any unscheduled assessment should be treated similarly to other evaluations.

Start dates

For all “time to event” variables, other than duration of response, the randomization/ date of treatment start will be used as the start date.

For the calculation of duration of response the following start date should be used:

- Date of first documented response is the assessment date of the first overall lesion response of CR (for duration of overall complete response) or CR / PR (for duration of overall response) respectively, when this status is later confirmed.

End dates

The end dates which are used to calculate ‘time to event’ variables are defined as follows:

- Date of death (during treatment as recorded on the treatment completion page or during follow-up as recorded on the study evaluation completion page or the survival follow-up page).
- Date of progression is the first assessment date at which the overall lesion response was recorded as progressive disease.
- Date of last adequate tumor assessment is the date the last tumor assessment with overall lesion response of CR, PR or SD which was made before an event or a censoring reason occurred. In this case the last tumor evaluation date at that assessment is used. If no post-baseline assessments are available (before an event or a censoring reason occurred) the date of randomization/start of treatment is used.

[redacted]

- Date of next scheduled assessment is the date of the last adequate tumor assessment plus the protocol specified time interval for assessments. This date may be used if back-dating is considered when the event occurred beyond the acceptable time window for the next tumor assessment as per protocol (see Section 14.1.3.2.9).

Example (if protocol defined schedule of assessments is 3 months): tumor assessments at baseline - 3 months - 6 months - missing - missing - PD. Date of next scheduled assessment would then correspond to 9 months.

- Date of discontinuation is the date of the end of treatment visit.
- Date of last contact is defined as the last date the patient was known to be alive. This corresponds to the latest date for either the visit date, lab sample date or tumor assessment date. If available, the last known date patient alive from the survival follow-up page is used. If no survival follow-up is available, the date of discontinuation is used as last contact date.
- Date of secondary anti-cancer therapy is defined as the start date of any additional (secondary) antineoplastic therapy or surgery.

14.1.3.2.9 Handling of patients with non-measurable disease only at baseline

It is possible that patients with only non-measurable disease present at baseline are entered into the study, either because of a protocol violation or by design (e.g. in Phase III studies with PFS as the primary endpoint). In such cases the handling of the response data requires special consideration with respect to inclusion in any analysis of endpoints based on the overall response evaluations.

It is recommended that any patients with only non-measurable disease at baseline should be included in the main (ITT) analysis of each of these endpoints.

Although the text of the definitions described in the previous sections primarily relates to patients with measurable disease at baseline, patients without measurable disease should also be incorporated in an appropriate manner. The overall response for patients with non-measurable disease is derived slightly differently according to Table 14-4.

Table 14-4 Overall lesion response at each assessment: patients with non-target disease only

Non-target lesions	New Lesions	Overall lesion response
CR	No	CR
Non-CR/Non-PD[1]	No	Non-CR/non-PD
UNK	No	UNK
PD	Yes or No	PD
Any	Yes	PD

[1] As defined in Section 14.1.2.4.

In general, the **non-CR/non-PD response** for these patients is considered equivalent to an SD response in endpoint determination. In summary tables for best overall response patients with only non-measurable disease may be highlighted in an appropriate fashion e.g. in particular by displaying the specific numbers with the non-CR/non-PD category.

[redacted]

In considering how to incorporate data from these patients into the analysis the importance to each endpoint of being able to identify a PR and/or to determine the occurrence and timing of progression needs to be taken into account.

For ORR it is recommended that the main (ITT) analysis includes data from patients with only non-measurable disease at baseline, handling patients with a best response of CR as “responders” with respect to ORR and all other patients as “non-responders”.

For PFS, it is again recommended that the main ITT analyses on these endpoints include all patients with only non-measurable disease at baseline, with possible sensitivity analyses which exclude these particular patients. Endpoints such as PFS which are reliant on the determination and/or timing of progression can incorporate data from patients with only non-measurable disease.

14.1.3.2.10 Sensitivity analyses

This section outlines the possible event and censoring dates for progression, as well as addresses the issues of missing tumor assessments during the study. For instance, if one or more assessment visits are missed prior to the progression event, to what date should the progression event be assigned? And should progression event be ignored if it occurred after a long period of a patient being lost to follow-up? It is important that the protocol and RAP specify the primary analysis in detail with respect to the definition of event and censoring dates and also include a description of one or more sensitivity analyses to be performed.

Based on definitions outlined in Section 14.1.3.2.8, and using the draft FDA guideline on endpoints (Clinical Trial Endpoints for the Approval of Cancer Drugs and Biologics-April 2005) as a reference, the following analyses can be considered:

[redacted]

Table 14-5 Options for event dates used in PFS, TTP, duration of response

Situation		Options for end-date (progression or censoring)[1] **(1) = default unless specified differently in the protocol or RAP**	Outcome
A	No baseline assessment	(1) Date of randomization/start of treatment[3]	Censored
B	Progression at or before next scheduled assessment	(1) Date of progression<br>(2) Date of next scheduled assessment[2]	Progressed<br>Progressed
C1	Progression or death after **exactly one** missing assessment	(1) Date of progression (or death)<br>(2) Date of next scheduled assessment[2]	Progressed<br>Progressed
C2	Progression or death after **two or more** missing assessments	(1) Date of last adequate assessment[2]<br>(2) Date of next scheduled assessment[2]<br>(3) Date of progression (or death)	Censored<br>Progressed<br>Progressed
D	No progression	(1) Date of last adequate assessment	Censored
E	Treatment discontinuation due to 'Disease progression' without documented progression, i.e. clinical progression based on investigator claim	(1) Ignore clinical progression and follow situations above<br>(2) Date of discontinuation (visit date at which clinical progression was determined)	As per above situations<br>Progressed
F	New anticancer therapy given	(1) Ignore the new anticancer therapy and follow situations above (ITT approach)<br>(2) Date of last adequate assessment prior to new anticancer therapy<br>(3) Date of secondary anti-cancer therapy<br>(4) Date of secondary anti-cancer therapy	As per above situations<br>Censored<br>Censored<br>Event
G	Deaths due to reason other than deterioration of 'Study indication'	(1) Date of last adequate assessment	Censored (only TTP and duration of response)

1. =Definitions can be found in Section 14.1.3.2.8.

2. =After the last adequate tumor assessment. "Date of next scheduled assessment" is defined in Section 14.1.3.2.8.

3. =The rare exception to this is if the patient dies no later than the time of the second scheduled assessment as defined in the protocol in which case this is a PFS event at the date of death.

The primary analysis and the sensitivity analyses must be specified in the protocol. Clearly define if and why options (1) are not used for situations C, E and (if applicable) F.

Situations C (C1 and C2): Progression or death after one or more missing assessments: The primary analysis is usually using options (1) for situations C1 and C2, i.e.

- (C1) taking the actual progression or death date, in the case of only one missing assessment.
- (C2) censoring at the date of the last adequate assessment, in the case of two or more consecutive missing assessments.

In the case of two or missing assessments (situation C2), option (3) may be considered jointly with option (1) in situation C1 as sensitivity analysis. A variant of this sensitivity analysis consists of backdating the date of event to the next scheduled assessment as proposed with option (2) in situations C1 and C2.

[redacted]

Situation E: Treatment discontinuation due to 'Disease progression' without documented progression: By default, option (1) is used for situation E as patients without documented PD should be followed for progression after discontinuation of treatment. However, option (2) may be used as sensitivity analysis. If progression is claimed based on clinical deterioration instead of tumor assessment by e.g. CT-scan, option (2) may be used for indications with high early progression rate or difficulties to assess the tumor due to clinical deterioration.

Situation F: New cancer therapy given: the handling of this situation must be specified in detail in the protocol. However, option (1) (ITT) is the recommended approach; events documented after the initiation of new cancer therapy will be considered for the primary analysis i.e. progressions and deaths documented after the initiation of new cancer therapy would be included as events. This will require continued follow-up for progression after the start of the new cancer therapy. In such cases, it is recommended that an additional sensitivity analysis be performed by censoring at last adequate assessment prior to initiation of new cancer therapy.

Option (2), i.e. censoring at last adequate assessment may be used as a sensitivity analysis. If a high censoring rate due to start of new cancer therapy is expected, a window of approximately 8 weeks performed after the start of new cancer therapy can be used to calculate the date of the event or censoring. This should be clearly specified in the analysis plan.

In some specific settings, local treatments (e.g. radiation/surgery) may not be considered as cancer therapies for assessment of event/censoring in PFS/TTP/DoR analysis. For example, palliative radiotherapy given in the trial for analgesic purposes or for lytic lesions at risk of fracture will not be considered as cancer therapy for the assessment of BOR and PFS analyses. The protocol should clearly state the local treatments which are not considered as antineoplastic therapies in the PFS/TTP/DoR analysis.

The protocol should state that tumor assessments will be performed every x weeks until radiological progression irrespective of initiation of new antineoplastic therapy. It is strongly recommended that a tumor assessment is performed before the patient is switched to a new cancer therapy.

Additional suggestions for sensitivity analyses

Other suggestions for additional sensitivity analyses may include analyses to check for potential bias in follow-up schedules for tumor assessments, e.g. by assigning the dates for censoring and events only at scheduled visit dates. The latter could be handled by replacing in Table 14-5 the "Date of last adequate assessment" by the "Date of previous scheduled assessment (from baseline)", with the following definition:

- **Date of previous scheduled assessment (from baseline)** is the date when a tumor assessment would have taken place, if the protocol assessment scheme was strictly followed from baseline, immediately before or on the date of the last adequate tumor assessment.

In addition, analyses could be repeated using the Investigators' assessments of response rather than the calculated response. The need for these types of sensitivity analyses will depend on

[redacted]

the individual requirements for the specific study and disease area and have to be specified in the protocol or RAP documentation.

14.1.4 Data handling and programming rules

The following section should be used as guidance for development of the protocol, data handling procedures or programming requirements (e.g. on incomplete dates).

14.1.4.1 Study/project specific decisions

For each study (or project) various issues need to be addressed and specified in the protocol or RAP documentation. Any deviations from protocol must be discussed and defined at the latest in the RAP documentation.

The proposed primary analysis and potential sensitivity analyses should be discussed and agreed with the health authorities and documented in the protocol (or at the latest in the RAP documentation before database lock).

14.1.4.2 End of treatment phase completion

Patients may voluntarily withdraw from the study treatment or may be taken off the study treatment at the discretion of the investigator at any time. For patients who are lost to follow-up, the investigator or designee should show "due diligence" by documenting in the source documents steps taken to contact the patient, e.g., dates of telephone calls, registered letters, etc.

The end of treatment visit and its associated assessments should occur within 7 days of the last study treatment.

Patients may discontinue study treatment for any of the following reasons:

- Adverse event(s)
- Lost to follow-up
- Physician decision
- Pregnancy
- Protocol deviation
- Technical problems
- Subject/guardian decision
- Progressive disease
- Study terminated by the sponsor
- Non-compliant with study treatment
- No longer requires treatment
- Treatment duration completed as per protocol (optional, to be used if only a fixed number of cycles is given)

Death is a reason which “**must**” lead to discontinuation of patient from trial.

[redacted]

14.1.4.3 End of post-treatment follow-up (study phase completion)

End of post-treatment follow-up visit will be completed after discontinuation of study treatment and post-treatment evaluations but prior to collecting survival follow-up.

Patients may provide study phase completion information for one of the following reasons:

- Adverse event
- Lost to follow-up
- Physician decision
- Pregnancy
- Protocol deviation
- Technical problems
- Subject/guardian decision
- Death
- Progressive disease
- Study terminated by the sponsor

14.1.4.4 Medical validation of programmed overall lesion response

In order to be as objective as possible the RECIST programmed calculated response assessment is very strict regarding measurement methods (i.e. any assessment with more or less sensitive method than the one used to assess the lesion at baseline is considered UNK) and not available evaluations (i.e. if any target or non-target lesion was not evaluated the whole overall lesion response is UNK unless remaining lesions qualified for PD). This contrasts with the slightly more flexible guidance given to local investigators (and to the central reviewers) to use expert judgment in determining response in these type of situations, and therefore as a consequence discrepancies between the different sources of response assessment often arise. To ensure the quality of response assessments from the local site and/or the central reviewer, the responses may be re-evaluated by clinicians (based on local investigator data recorded in eCRF or based on central reviewer data entered in the database) at Novartis or external experts. In addition, data review reports will be available to identify assessments for which the investigators' or central reader's opinion does not match the programmed calculated response based on RECIST criteria. This may be queried for clarification. However, the investigator or central reader's response assessment will never be overruled.

If Novartis elect to invalidate an overall lesion response as evaluated by the investigator or central reader upon internal or external review of the data, the calculated overall lesion response at that specific assessment is to be kept in a dataset. This must be clearly documented in the RAP documentation and agreed before database lock. This dataset should be created and stored as part of the 'raw' data.

Any discontinuation due to 'Disease progression' without documentation of progression by RECIST criteria should be carefully reviewed. Only patients with documented deterioration of symptoms indicative of progression of disease should have this reason for discontinuation of treatment or study evaluation.

[redacted]

14.1.4.5 Programming rules

The following should be used for programming of efficacy results:

14.1.4.5.1 Calculation of 'time to event' variables

Time to event = end date - start date + 1 (in days)

When no post-baseline tumor assessments are available, the date of randomization/start of treatment will be used as end date (duration = 1 day) when time is to be censored at last tumor assessment, i.e. time to event variables can never be negative.

14.1.4.5.2 Incomplete assessment dates

All investigation dates (e.g. X-ray, CT scan) must be completed with day, month and year.

If one or more investigation dates are incomplete but other investigation dates are available, this/these incomplete date(s) are not considered for calculation of the assessment date (and assessment date is calculated as outlined in Section 14.1.3.2.8). If all measurement dates have no day recorded, the 1st of the month is used.

If the month is not completed, for any of the investigations, the respective assessment will be considered to be at the date which is exactly between previous and following assessment. If a previous and following assessment is not available, this assessment will not be used for any calculation.

14.1.4.5.3 Incomplete dates for last known date patient alive or death

All dates must be completed with day, month and year. If the day is missing, the 15th of the month will be used for incomplete death dates or dates of last contact.

14.1.4.5.4 Non-target lesion response

If no non-target lesions are identified at baseline (and therefore not followed throughout the study), the non-target lesion response at each assessment will be considered 'not applicable (NA)'.

14.1.4.5.5 Study/project specific programming

The standard analysis programs need to be adapted for each study/project.

14.1.4.5.6 Censoring reason

In order to summarize the various reasons for censoring, the following categories will be calculated for each time to event variable based on the treatment completion page, the study evaluation completion page and the survival page.

For survival the following censoring reasons are possible:

- Alive
- Lost to follow-up

For PFS and TTP (and therefore duration of responses) the following censoring reasons are possible:

[redacted]

- Ongoing without event
- Lost to follow-up
- Withdrew consent
- Adequate assessment no longer available*
- Event documented after two or more missing tumor assessments (optional, see Table 14-5)
- Death due to reason other than underlying cancer *(only used for TTP and duration of response)*
- Initiation of new anti-cancer therapy

* Adequate assessment is defined in Section 14.1.3.2.8. This reason is applicable when adequate evaluations are missing for a specified period prior to data cut-off (or prior to any other censoring reason) corresponding to the unavailability of two or more planned tumor assessments prior to the cut-off date. The following clarifications concerning this reason should also be noted:

- This may be when there has been a definite decision to stop evaluation (e.g. reason="Sponsor decision" on study evaluation completion page), when patients are not followed for progression after treatment completion or when only UNK assessments are available just prior to data cut-off).
- The reason "Adequate assessment no longer available" also prevails in situations when another censoring reason (e.g. withdrawal of consent, loss to follow-up or alternative anti-cancer therapy) has occurred more than the specified period following the last adequate assessment.
- This reason will also be used to censor in case of no baseline assessment.

14.1.5 References (available upon request)

Dent S, Zee (2001) application of a new multinomial phase II stopping rule using response and early progression, J Clin Oncol; 19: 785-791.

Eisenhauer E, et al (2009) New response evaluation criteria in solid tumors: revised RECIST guideline (version 1.1). European Journal of Cancer, Vol.45: 228-47.

Ellis S, et al (2008) Analysis of duration of response in oncology trials. Contemp Clin Trials 2008; 29: 456-465.

EMA Guidance: 2012 Guideline on the evaluation of anticancer medicinal products in man.

FDA Guidelines: 2005 Clinical Trial Endpoints for the Approval of Cancer Drugs and Biologics, April 2005.

FDA Guidelines: 2007 Clinical Trial Endpoints for the Approval of Cancer Drugs and Biologics, May 2007.

Morgan TM (1988) Analysis of duration of response: a problem of oncology trials. Cont Clin Trials; 9: 11-18.

Therasse P, Arbuck S, Eisenhauer E, et al (2000) New Guidelines to Evaluate the Response to Treatment in Solid Tumors, Journal of National Cancer Institute, Vol. 92; 205-16.

[redacted]
